# Ion pair extractant selective for LiCl and LiBr†

**DOI:** 10.1039/d4sc03760j

**Published:** 2024-08-12

**Authors:** Nam Jung Heo, Ju Hyun Oh, Aimin Li, Kyounghoon Lee, Qing He, Jonathan L. Sessler, Sung Kuk Kim

**Affiliations:** a Department of Chemistry, Research Institute of Natural Sciences, Gyeongsang National University Jinju 52828 Korea sungkukkim@gnu.ac.kr; b State Key Laboratory of Chemo/Biosensing and Chemometrics, College of Chemistry and Chemical Engineering, Hunan University Changsha 410082 P. R. China; c Department of Chemistry Education, Research Institute of Natural Sciences, Gyeongsang National University Jinju 52828 Korea; d Department of Chemistry, The University of Texas at Austin 105 E. 24th Street-Stop A5300 Austin Texas 78712-1224 USA sessler@cm.utexas.edu

## Abstract

Improved methods for achieving the selective extraction of lithium salts from lithium sources, including rocky ores, salt-lake brines, and end-of-life lithium-ion batteries, could help address projected increases in the demand for lithium. Here, we report an ion pair receptor (2) capable of extracting LiCl and LiBr into an organic receiving phase both from the solid state and from aqueous solutions. Ion pair receptor 2 consists of a calix[4]pyrrole framework, which acts as an anion binding site, linked to a phenanthroline cation binding motif *via* ether linkages. Receptor 2 binds MgBr_2_ and CaCl_2_ with high selectivity over the corresponding lithium salts in a nonpolar aprotic solvent. The preference for Mg^2+^ and Ca^2+^ salts is reversed in polar protic media, allowing receptor 2 to complex LiCl and LiBr with high selectivity and affinity in organic media containing methanol or water. The effectiveness of receptor 2 as an extractant for LiCl and LiBr under liquid–liquid extraction (LLE) conditions was found to be enhanced by the presence of other potentially competitive salts in the aqueous source phase.

## Abstract 2

An ion pair receptor is reported that is capable of extracting LiCl and LiBr selectively from aqueous phases; the extraction efficiency is enhanced by the presence of other competitive salts.

## Graphical abstract

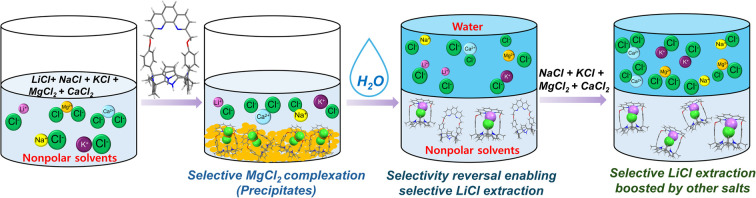

## Introduction

The lithium ion (Li^+^) supports key features of modern life; it is an essential component in lithium ion batteries (LIBs), ceramics, and lubricating greases, and is used as a pharmaceutical agent in treating depressive illness.^1–6^ Currently rechargeable LIBs account for approximately 80% of the end use of lithium.^7^ According to the U.S. Geological Survey (USGS), global consumption of lithium rose from approximately 95 000 tons in 2021 to 134 000 tons in 2022.^7^ While the global demand for lithium is increasing, the available lithium reserves remain limited. In addition, selective separation of the lithium ion from its sources requires an energy- and labor-intensive process, and can be time-consuming.^8,9^ In principle, lithium may be separated from rocky ores, salt lakes, brines, and sea water.^8–10^ When rocky ores or clays are used as the lithium source, roasting at a high temperature (1100 °C) is followed by baking at 250 °C in acid.^8^ Undesired salts are then removed *via* several energy- and water-intensive steps that are a source of environmental concern.^11–13^ The ocean is the largest source of lithium; however, selective extraction of lithium from seawater is challenging because of the very low concentration of the lithium cation amounting to only 0.1–0.2 ppm while other potentially competitive ions exist at much higher concentrations.^14–19^ At present, salt lake brines supply the majority of the commercial lithium. However, the extraction of lithium from brine reservoirs typically requires evaporation of residual water over a period of months to years.^20–26^ A huge amount of water is also necessary to remove unwanted ions and contaminants. Because of the shortcomings of conventional extraction processes, efforts are being devoted increasingly to so-called direct lithium extraction (DLE) methods. In this context, the use of porous lithium sorbents and ion exchange materials has attracted attention because of their relative simplicity and potential to operate at relatively low levels of environmental stress.^27,28^ Unfortunately, most DLE methods developed thus far require additional processing steps to free the lithium ions from the lithium adsorbing materials.^27,28^ Therefore, so-called liquid–liquid extraction (LLE) and solid–liquid extraction (SLE), wherein an extractant capable of selectively complexing lithium salts is used to promote DLE, continue to attract interest. In principle, these approaches could enable the direct and selective extraction of lithium salts from mixed salt solid phases or brines. Nevertheless, it remains challenging to design and construct an extractant possessing the ability to extract selectively lithium salts due to the small size of the lithium cation and its relatively high hydration energy (*Δ*_hyd_G* = −475 kJ mol^−1^), as well as the interference of other competing cations, such as Na^+^, K^+^, Mg^2+^, and Ca^2+^.^29–31^ For instance, Mg^2+^ not only has a similar ionic radius (72 pm for Mg^2+^*vs.* 69 pm for Li^+^), it also has a higher net charge, and is typically present at ≥8× the concentration of Li^+^ in most salt lake brines.^23,29^ The likely presence of Mg^2+^ and other potential interferants underscores the challenge associated with designing Li^+^-selective extractants. One way to meet this challenge could involve the use of ion pair receptors. Here we report a new phenanthroline-strapped calix[4]pyrrole (2) that acts as a selective receptor for LiCl and LiBr in polar media and which promotes lithium salt extraction under LLE conditions.

**Fig. 1:**
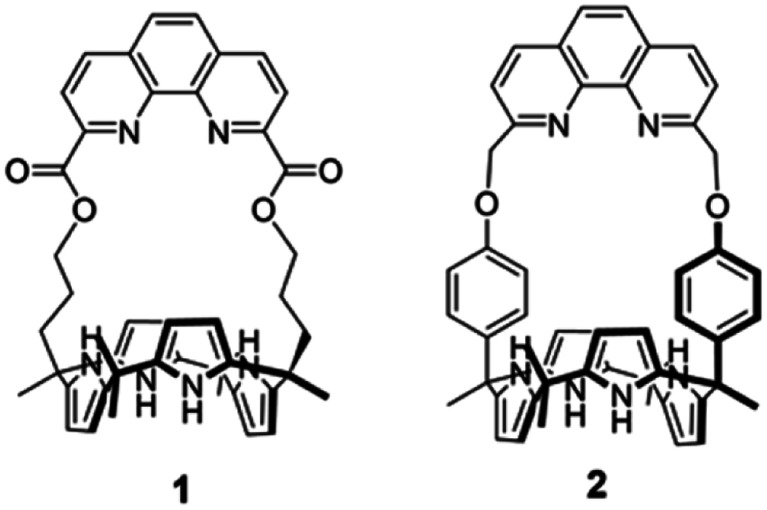
Chemical structures of ion pair receptors 1 and 2.

Ion pair receptors are systems with an ability to complex concurrently both a cation and an anion. Appropriately designed ion pair receptors display enhanced selectivity and affinity for target ions relative to cation or anion receptors, systems that bind either a cation or an anion, but not both.^32–36^ Although numerous ion pair receptors capable of binding various alkali metal salts have been reported to date, only a very small number of receptors were found to bind lithium salts with sufficient affinity and selectivity to permit effective SLE or LLE of Li^+^ salts.^37–39^ Unfortunately, even in the most favorable cases the actual lithium extraction efficiencies proved very low. For instance, we reported ion pair receptors based on calix[4]pyrroles strapped with pyridine-fused multi-aromatic rings containing a methoxybenzene group. These heteroditopic ion pair receptors proved capable of extracting LiCl or LiNO_3_ from the solid state into nitrobenzene-*d*_5_ or dichloromethane-*d*_2_ under solid–liquid extraction conditions provided the salts were present in excess.^37,39^ The phenanthroline-strapped calix[4]pyrrole (1) having ester likers was found to extract LiCl into chloroform from an aqueous solution containing near-saturated quantities of LiCl, but not at lower source phase concentrations.^39^ In the case of receptor 1, the presence of the electron-withdrawing ester groups directly linked to the phenanthroline cation binding site was thought to reduce the inherent Li^+^ affinity accounting for the limits on its extraction ability. As inferred from a solid state X-ray crystal structure and solution phase ^1^H NMR spectral studies, the ester carbonyl oxygen atoms present in receptor 1 do not participate in complexation with lithium.^39^ We thus considered it likely that an analogue of 1 that incorporated oxygen donor sites within the tethering subunits that link the phenanthroline strap to the calix[4]pyrrole core would prove more effective as an ion pair receptor for lithium salts, such as LiCl and LiBr. With such considerations in mind, we designed a new ion pair receptor (2) wherein the ester groups present in 1 are replaced by phenoxy ether linkages (Fig. 1). The relatively rigid nature of the phenoxy linkers was also expected to provide for increased structural preorganization within the receptor framework leading to improved LiCl and LiBr recognition. As detailed below, receptor 2 binds LiCl and LiBr with high selectivity and with an affinity (*K*_a_ > 10^5^ M^−1^ for LiCl in 10% methanol in chloroform-*d*) that exceeds that of receptor 1 (*K*_a_ = 180 ± 7), as well as all other lithium ion receptors of which we are aware. Receptor 2 also acts as an effective LLE extractant promoting the transfer of LiCl and LiBr from aqueous solutions into dichloromethane or nitrobenzene receiving phases.

## Results and discussion

We previously reported that the ion pair receptor 1, a phenanthroline-strapped calix[4]pyrrole with ester linkers, was able to extract LiCl from LiCl-saturated aqueous solutions into a chloroform layer.^39^ However, receptor 1 failed to extract LiCl from aqueous solutions containing relatively low concentrations of LiCl. Moreover, receptor 1 was not examined for its LiCl selectivity in the presence of MgX_2_, CaX_2_, NaX, or KX (X = Cl and Br), which coexist in most lithium sources, under LLE conditions.^39^ As noted above, receptor 2, wherein relatively rigid phenoxy groups serve to link the phenanthroline cation and calix[4]pyrrole anion recognition subunits, was prepared in an effort to address these shortcomings.

**Scheme 1:**
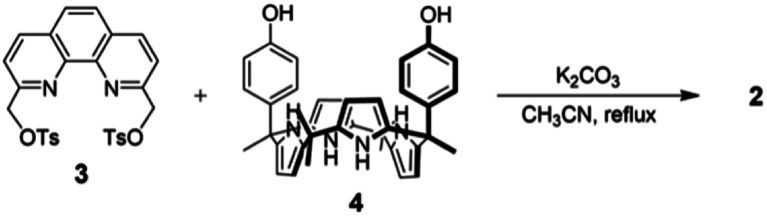
Synthesis of receptor 2.

The synthesis of receptor 2 is summarized in Scheme 1. Briefly, phenanthroline ditosylate 3 and *cis*-bisphenolic calix[4]pyrrole 4 were prepared following known literature procedures (Scheme 1).^40,41^ Reaction of compound 3 with calix[4]pyrrole 4 in the presence of K_2_CO_3_ as a base in acetonitrile gave the desired ion pair receptor 2 in 11% yield. Receptor 2 was characterized by means of ^1^H and ^13^C NMR spectroscopies and high resolution QTOF (quadrupole time of flight) mass spectrometry, as well as a single crystal X-ray diffraction analysis of its LiBr complex.

Initially, we examined the ability of receptor 2 to bind various alkali and alkaline earth metal chloride salts, including LiCl, NaCl, KCl, MgCl_2_, and CaCl_2_ in CH_3_OH/CDCl_3_ (1 : 9, v/v) using ^1^H NMR spectroscopy. This specific solvent system was chosen with consideration of the solubility of both the receptor and the test salts in mind. Upon exposure of receptor 2 to excess LiCl (*ca.* 100 equiv.), noticeable chemical shift changes were observed in the proton signals of both the calix[4]pyrrole subunit and the phenanthroline group, findings leading us to conclude that receptor 2 forms an ion pair complex with LiCl (Fig. S1 and S2, see the ESI† for details). By contrast, no chemical shift movements were seen in the presence of NaCl and KCl, which was taken as evidence for receptor 2 being incapable of complexing NaCl and KCl. In contrast, in the presence of MgCl_2_ (*ca.* 100 equiv.), roughly 16% of receptor 2 forms a complex with MgCl_2_ under these conditions (CH_3_OH/CDCl_3_; 1 : 9, v/v), which leads us to suggest that the affinity of receptor 2 for MgCl_2_ is very low (*K*_a_ < 5 M^−1^) (Fig. S1†).^42,43^ In analogy to what was seen with LiCl, Mg^2+^ is presumed to be bound to the phenanthroline unit while one of two Cl^−^ anions is bound to the calix[4]pyrrole moiety *via* hydrogen bonds (Fig. S1 and S2; see the ESI† for details). Distinct chemical shift changes were also observed in the ^1^H NMR spectrum when receptor 2 was treated with CaCl_2_ in CH_3_OH/CDCl_3_ (1 : 9, v/v) (Fig. S1†). These ^1^H NMR spectral changes are consistent with the Ca^2+^ cation only, and not the Cl^−^ anion, being complexed within receptor 2 (Fig. S2†). A follow up ^1^H NMR spectral titration with CaCl_2_ provided further support for this binding mode (Fig. S3; see the ESI† for details). This non-ion pair binding mode stands in contrast to what was seen in the cases of LiCl and MgCl_2_ (Fig. S2†). Taken in concert, these findings are thought to reflect a cation recognition site that is near-optimal for Li^+^ cation complexation, somewhat small for fully effective Na^+^ and K^+^ recognition, and essentially able to accommodate only Ca^2+^ without Cl^−^ being co-bound.

We also investigated the capability of receptor 2 to complex the bromide salts of alkali and alkaline earth metal cations, including LiBr, NaBr, KBr, MgBr_2_ and CaBr_2_, in 10% methanol in chloroform-*d*. In contrast to what was seen with the corresponding chloride salts, upon exposure of receptor 2 to the respective bromide salts, only LiBr gave rise to chemical shift movements attributable to ion pair complexation (Fig. S4†). This finding is taken as evidence that receptor 2 is capable of binding LiBr selectively over other test bromide salts. We also examined the binding selectivity of receptor 2 for other lithium halide salts, *i.e.*, LiF, LiCl, LiBr, and LiI, in CH_3_OH/CDCl_3_ (1 : 9, v/v). Addition of LiCl and LiBr produced chemical shift changes for the signals of receptor 2 in the ^1^H NMR spectra ascribable to co-binding of the lithium cations and the halide anions while LiF caused no appreciable chemical shift changes (Fig. S5†). When receptor 2 was exposed to LiI, only the lithium cation was bound to the phenanthroline moiety without the iodide anion being co-complexed by the calix[4]pyrrole subunit (Fig. S5; see the ESI† for details).

To quantify the ability of receptor 2 to bind cations and anions as well as various test ion pairs, we performed ^1^H NMR spectroscopic titrations in CH_3_OH/CDCl_3_ (1 : 9, v/v). For instance, upon the titration of receptor 2 with LiCl, the resulting ^1^H NMR spectral changes support the conclusion receptor 2 binds LiCl quantitatively with a 1 : 1 binding stoichiometry *via* a binding-release equilibrium that is slow on the NMR time scale (Fig. S6; see the ESI† for details). The association constant for LiCl approximated from this titration experiment was found to be *K*_a_ > 10^5^ M^−1^. This value is at least three orders of magnitude larger than what was found in the case of receptor 1 (Table 1 and Fig. S7†).^42^

**Table 1:** Association constants (*K*_a_, M^−1^)^a^ corresponding to the interaction of receptor 2 with the lithium cation and selected halide anion salts as determined in CH_3_OH/CDCl_3_ (1 : 9, v/v)

Host^b^	Guest	*K*_a_ (M^−1^)
2	Cl^−^	No binding
2	Br^−^	No binding
2	Li^+^	<5^c^
1	LiCl	180 ± 7^c^
2	LiCl	>10^5^
1	LiBr	24 ± 1^c^
2	LiBr	63 ± 3^c^
2 + Li^+^	Cl^−^	>10^5^
2 + Li^+^	Br^−^	148 ± 5^c^
2 + Cl^−^	Li^+^	>10^5^
2 + Br^−^	Li^+^	95 ± 3^c^

a Values were obtained from ^1^H NMR spectroscopic titrations of 2.

b Unless otherwise indicated, the anions and lithium cations were used in the forms of their respective tetrabutylammonium and perchlorate salts.

c The *K*_a_ value was approximated using BindFit v0.5 available from https://app.supramolecular.org/bindfit.

Receptor 2 was also found to bind LiBr, albeit with low affinity relative to LiCl and *via* an equilibrium process that is fast on the NMR timescale (Fig. S8, see the ESI† for details). The corresponding LiBr association constant was calculated to be *K*_a_ = 63 ± 3 M^−1^.^43^ The relatively low affinity for LiBr is presumably because the Br^−^ anion is too large to be co-complexed with the Li^+^ cation effectively within the receptor cavity. However, this association constant for LiBr is 2.6× larger than that of receptor 1 (Table 1 and Fig. S9†). The association constants for MgCl_2_ and CaCl_2_ were likewise determined to be *K*_a_ < 5 M^−1^ for both salts in 10% CH_3_OH in CDCl_3_ (Fig. S1 and S3†).^43^ Again, this finding is ascribed to a mismatch with the binding cavity present in receptor 2 as well as to larger solvation energies of the divalent cations (*vide infra*).

We also investigated the interactions of receptor 2 with Cl^−^, Br^−^, and Li^+^ with non-coordinating counter ions in 10% CH_3_OH in CDCl_3_. When receptor 2 was treated with excess Cl^−^ and Br^−^ (as their TBA^+^ (tetrabutylammonium) salts), no appreciable chemical shift changes were observed in the ^1^H NMR spectrum, a finding taken as evidence that receptor 2 fails to bind these halide anion salts in this protic solvent system (Fig. S10 and S11†). In contrast, in the presence of excess Li^+^ (as its ClO_4_^−^ (perchlorate anion) salt), the CH proton signal (H_a_) of the phenanthroline subunit of receptor 2 underwent a slight downfield shift (Δ*δ* = 0.04 ppm). Although modest, this change is thought to reflect Li^+^ cation complexation by the phenanthroline group (Fig. S10†). The association constant of receptor 2 for LiClO_4_ was calculated from this ^1^H NMR spectral titration to be <5 M^−1^ (Fig. S12†).^43^ On the basis of these studies, we conclude that receptor 2 binds the LiCl and LiBr ion pairs far more effectively than the individual ions, Cl^−^, and Br^−^, and Li^+^, when the latter were tested using a non-coordinating counter ion. These findings are rationalized in terms of the ion pair complexes with LiCl and LiBr being stabilized by electrostatic attractions between the co-bound anion and cation.

**Fig. 2:**
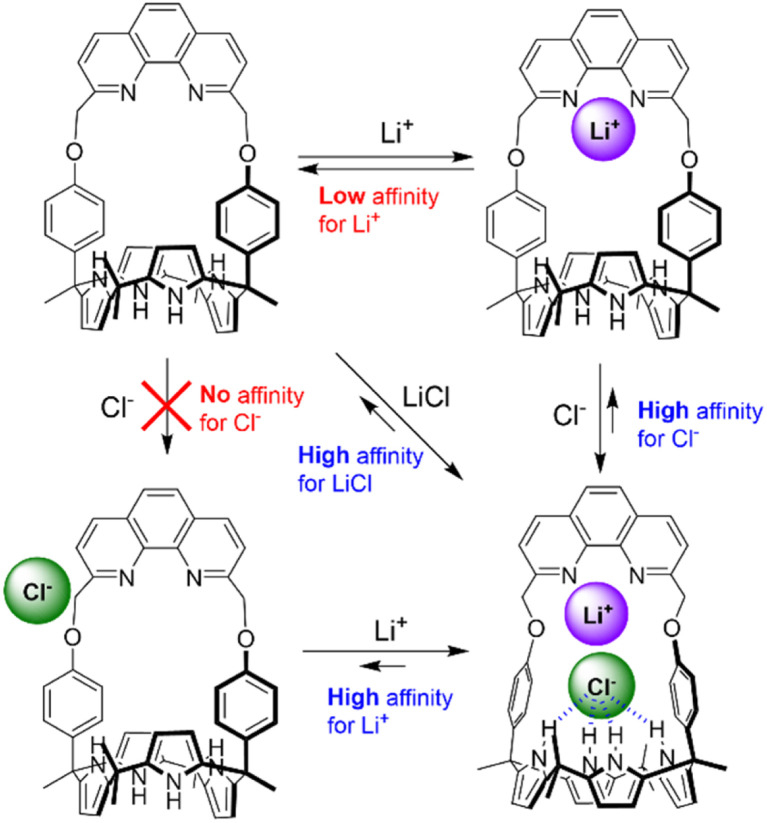
Proposed ion binding modes of receptor 2 in the presence of separately Li^+^ and Cl^−^ (added with a non-coordinating counter ion) and in the presence of both Li^+^ and Cl^−^ in CH_3_OH/CDCl_3_ (1 : 9, v/v) as inferred from ^1^H NMR spectroscopic studies.

Consistent with the above conclusion, the ability of receptor 2 to bind the Cl^−^ and Br^−^ anions was markedly improved in the presence of the Li^+^ cation and *vice versa* (Fig. 2). For instance, when receptor 2 was titrated with Cl^−^ in the presence of Li^+^ in 10% CH_3_OH in CDCl_3_, a new set of proton signals attributable to the LiCl complex of receptor 2 emerged with saturation being achieved upon the addition of 1.0 equiv. of Cl^−^ (Fig. S13†). The association constant of receptor 2 for Cl^−^ in the presence of Li^+^ was found to be >10^5^ M^−1^ on the basis of this ^1^H NMR spectral titration experiment.^42^ Receptor 2 was also found to bind the Br^−^ anion in the presence of the Li^+^ cation (*ca.* 50 equiv.) with an association constant of *K*_a_ = 148 ± 5 M^−1^ (Fig. S14†).^43^ This value is considerably larger than what was seen in its absence (no affinity for Br^−^; *vide supra*). We thus conclude that co-complexation of Cl^−^ and Br^−^ with Li^+^ significantly enhances the affinity of 2 for these two halide anions, which are otherwise incapable of binding to the receptor (Fig. 2).

The affinity of receptor 2 for Li^+^ was also found to be significantly enhanced in the presence of Cl^−^ and Br^−^. On the basis of the ^1^H NMR spectral titration of receptor 2 with LiClO_4_ in the presence of TBACl (5.0 equiv.), the association constant of receptor 2 for Li^+^ was estimated to be >10^5^ M^−1^ (Fig. S15; see the ESI† for details), a value that is higher than that measured in the absence of Cl^−^ by >100 000-fold (Table 1).^42^ In contrast, the affinity of receptor 2 for Li^+^ was enhanced in the presence of Br^−^ by only approx. 20-fold (Fig. S16† and Table 1). We thus conclude that cooperativity between the cation and anion binding sites within receptor 2 plays a crucial role in regulating the ion binding affinity of the receptor for appropriately chosen lithium halide ion pairs, with the effect being particularly dramatic in the case of LiCl, but also noteworthy for LiBr.

**Fig. 3:**
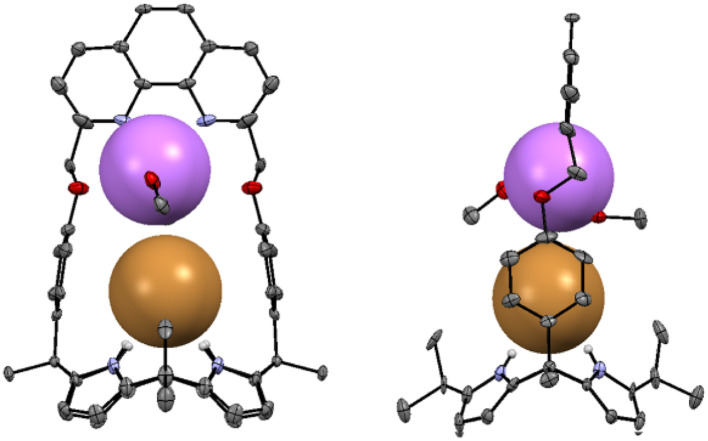
Two different views of the X-ray crystal structure of the 2·LiBr. Thermal ellipsoids are scaled to the 50% probability level. Most hydrogen atoms are omitted for clarity.

Further evidence that receptor 2 forms an ion pair complex with LiBr came from a single crystal X-ray diffraction analysis. Single crystals of the complex 2·LiBr appropriate for an X-ray diffraction analysis were grown by allowing a mixture of CHCl_3_ and CH_3_OH containing 2 and an excess LiBr to evaporate slowly. The resulting crystal structure revealed that the Br^−^ anion is bound to the calix[4]pyrrole moiety *via* hydrogen bonds with N–H⋯Br^−^ distances of 2.50–2.51 Å while the Li^+^ cation is coordinated not only to the phenanthroline nitrogen atoms but also to the ether oxygen atoms with N⋯Li^+^ distances of 1.91 Å and 2.34 Å and O⋯Li^+^ distances of 2.627 Å and 3.269 Å, respectively (Fig. 3). The Li^+^ cation is further stabilized by the coordination of two methanol molecules. The Li^+^ cation was also found to interact directly with the co-bound Br^−^ anion at a distance of 4.02 Å.

We also tested receptor 2 for its ability to solubilize and extract the solid LiCl and LiBr into dichloromethane-*d*_2_ (CD_2_Cl_2_). When receptor 2 along with LiCl or LiBr (*ca.* 100 equiv.) was subjected to sonication for 1 hour in CD_2_Cl_2_, distinct ^1^H NMR spectral changes attributable to the formation of ion pair complexes 2·LiCl and 2·LiBr were seen (Fig. S17†). These ion pair complexes proved soluble in CD_2_Cl_2_ enabling receptor 2 to extract LiCl and LiBr into this organic phase under solid–liquid extraction conditions. When an equimolar ratio of LiCl and LiBr (*ca.* 100 equiv. each) was used, receptor 2 was found to complex both LiCl and LiBr at a nearly 1 : 1 ratio in CD_2_Cl_2_. This proved true in spite of the greater affinity displayed for LiCl over LiBr in CH_3_OH/CDCl_3_; 1/9, v/v (Table 1). This seeming disparity is rationalized in terms of the relatively small lattice energy of LiBr in CD_2_Cl_2_. On the other hand, release of the lithium cation from 2·LiCl could be triggered in CD_2_Cl_2_*via* the addition of the fluoride anion (as its tetrabutylammonium (TBA^+^) salt). This treatment enables recovery of Li^+^ in the form of its insoluble LiF salt by filtration (Fig. S18†). The process could be followed by ^1^H NMR spectroscopy (Fig. S18; see the ESI† for details).

The selectivity of 2 for the chloride and bromide salts of alkali and alkaline earth cations was also evaluated under SLE conditions using dichloromethane-*d*_2_ as the receiving phase. Receptor 2 is soluble in this solvent, whereas the test salts are insoluble. In this aprotic solvent, receptor 2 exhibited different binding behavior as compared to 10% CH_3_OH in CDCl_3_. Upon treatment of receptor 2 with approx. 500 equiv. each of LiCl, NaCl, KCl, MgCl_2_, and CaCl_2_ in CD_2_Cl_2_ (separate studies), only LiCl induced chemical shift changes in the spectrum that could be readily interpreted in terms of LiCl complexation (Fig. S19†). In contrast, NaCl, KCl, and CaCl_2_ gave rise to no appreciable chemical shift changes in the ^1^H NMR spectrum of 2. Meanwhile, treatment with MgCl_2_ produced an orange precipitate (Fig. S19†). This latter finding is interpreted in terms of receptor 2 forming an ion pair complex with MgCl_2_ that is insoluble in CD_2_Cl_2_. We next monitored the changes in the ^1^H NMR spectrum of 2 when treated with a mixture of LiCl, NaCl, KCl, MgCl_2_, and CaCl_2_ salts (*ca.* 100 equiv. each) in CD_2_Cl_2_. Under these conditions, an orange precipitate was formed with no discernable proton signals being seen in the ^1^H NMR spectrum of the residual CD_2_Cl_2_ layer. Given the analogy to what was seen when receptor 2 was treated with MgCl_2_ alone, we interpret these findings in terms of receptor 2 capturing MgCl_2_ in preference to the other chloride salts with this nominal selectivity being driven in part by solubility considerations (Fig. S20†).

In case of the corresponding bromide salts, receptor 2 was found to extract LiBr and MgBr_2_ in the solid state into CD_2_Cl_2_ but *via* different apparent binding modes. For instance, when exposed to LiBr in CD_2_Cl_2_, receptor 2 exhibited ^1^H NMR spectral changes consistent with the formation of 2·LiBr where the Li^+^ and Br^−^ are bound to the phenanthroline nitrogen atoms and the calix[4]pyrrole NH protons, respectively (Fig. S20†). In contrast, exposure of 2 to MgBr_2_ led the signal assignable to the calix[4]pyrrole NH protons to undergo a relatively small downfield shift (Δ*δ* = 0.26 ppm for MgBr_2_*vs.* Δ*δ* = 3.31 ppm for LiBr, respectively) while the proton signals of the phenanthroline and the phenoxy CH hydrogens were seen to undergo noticeable downfield shifts (Fig. S20†). These ^1^H NMR spectral changes provide support for the notion that only the Mg^2+^ cation is bound to the receptor with the two Br^−^ counter anions being located outside the receptor cavity. In contrast, treatment of receptor 2 with CaBr_2_ led to formation of a white solid with no observable proton signals appearing in the ^1^H NMR spectrum of 2. Again, this finding is consistent with the formation of a strong, insoluble complex with CaBr_2_ (Fig. S20†). A similar phenomenon took place when receptor 2 in CD_2_Cl_2_ was treated with an equimolar mixture of LiBr, NaBr, KBr, MgBr_2_, and CaBr_2_ (*ca.* 100 equiv. each) (Fig. S20†). We thus conclude that receptor 2 complexes CaBr_2_ with high selectivity among the various test bromide salts.

**Fig. 4:**
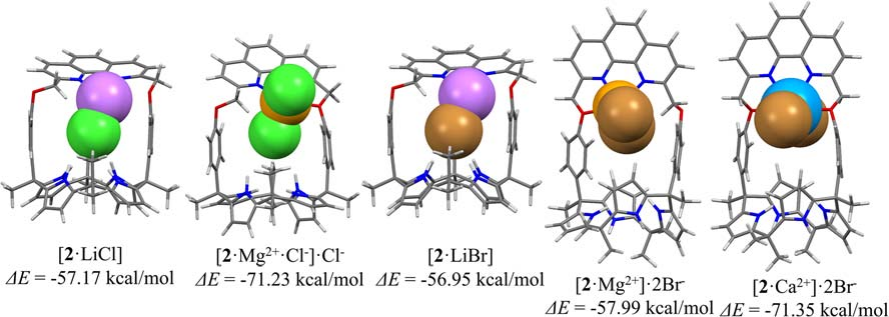
Optimized structures of the complexes of receptor 2 with LiCl, MgCl_2_, LiBr, MgBr_2_, and CaBr_2_ as calculated in the gas phase and the corresponding computed complexation energies for the interaction of receptor 2 with the ion pairs in question.

**Fig. 5:**
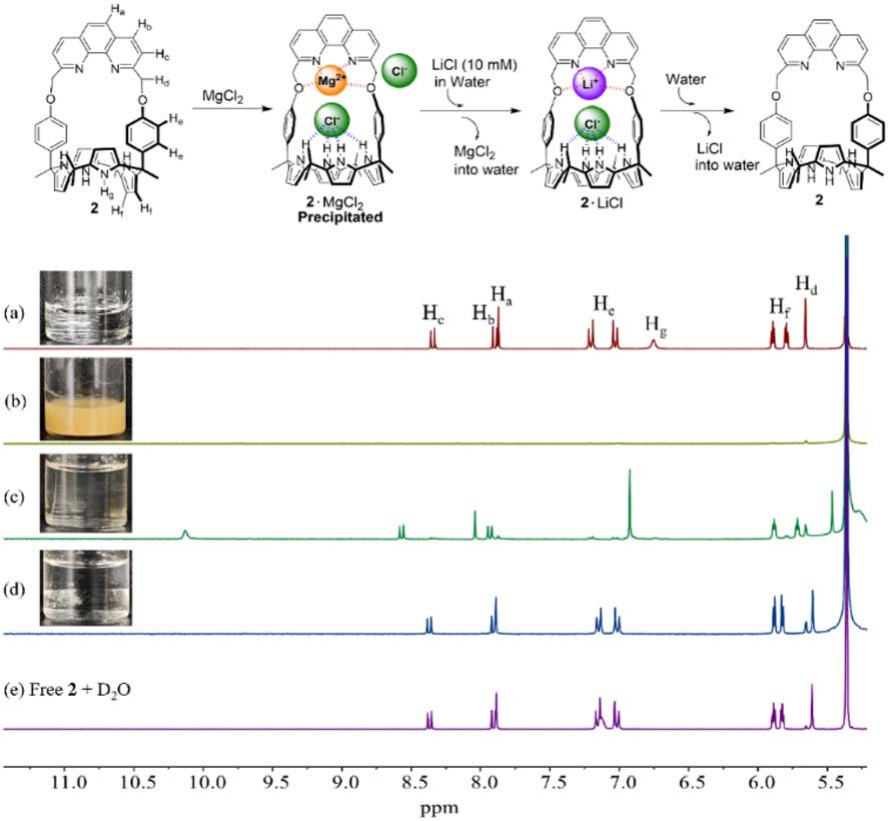
Partial ^1^H NMR spectra of CD_2_Cl_2_ solutions of (a) 2 (3 mM) only, (b) 2 + MgCl_2_ (500 equiv.), (c) solution (b) after contacting with 10 M LiCl aqueous solution, (d) solution (c) after removing an aqueous phase and then contacting with an ion-free aqueous D_2_O solution, and (e) a CD_2_Cl_2_ solution of free 2 after contacting with an ion-free aqueous D_2_O solution. The asterisk (*) denotes the residual CH_2_Cl_2_ peak in the NMR solvent.

The high selectivity of receptor 2 for MgCl_2_ over LiCl and for CaBr_2_ over LiBr and MgBr_2_ observed in CD_2_Cl_2_ was rationalized in terms of the associated binding energies and geometries of the resulting complexes obtained *via* density functional theory (DFT) calculations carried out in the gas phase at the X3LYP/6-31g* level (Fig. 4; see the ESI† for details). For instance, the complexation energies of receptor 2 for LiCl and MgCl_2_ were computed to be −57.17 kcal mol^−1^ and −71.23 kcal mol^−1^, respectively. In case of the optimized structure of the MgCl_2_ complex, one of two chloride anions was bound to the calix[4]pyrrole subunit of receptor 2 interacting directly with the magnesium cation coordinated by the phenanthroline nitrogen atoms as well as one ether oxygen atom (Fig. 4; *cf.* Fig. S1 and S2†). In contrast, the other chloride anion was located outside the receptor cavity forming a contact ion pair with the magnesium cation. The complexation energy of receptor 2 for MgCl_2_ proved larger than that for LiCl by −14.06 and could account for the selective binding of the receptor for MgCl_2_ over LiCl; however, solubility considerations are ignored in this analysis. The stabilization energies of receptor 2 upon complexing various bromide salts were similarly calculated to be −56.95 kcal mol^−1^ for LiBr, −57.99 kcal mol^−1^ for MgBr_2_, and −71.35 kcal mol^−1^ for CaBr_2_, respectively (Fig. 5). These computed values could account for the selectivity seen for CaBr_2_ over LiBr or MgBr_2_; however, as above, effects such as solvation and solubility are not considered.

The selectivity of receptor 2 seen in 10% CH_3_OH in CDCl_3_ is considered to reflect solvation effects. Based on their respective hydration energies (*Δ*_hyd_G* = −1830 kJ mol^−1^ for Mg^2+^, *Δ*_hyd_G* = −1505 kJ mol^−1^ for Ca^2+^, and *Δ*_hyd_G* = −475 kJ mol^−1^ for Li^+^), Mg^2+^ and Ca^2+^ are presumed to be more strongly solvated by methanol molecules than Li^+^.^29^ This strong solvation is thought to reduce the binding interactions with receptor 2 leading to the observed selective binding of LiCl and LiBr in CH_3_OH/CDCl_3_ (1/9, v/v). Support for this presumption came from a ^1^H NMR spectroscopic analysis. For instance, when receptor 2 was treated with an equimolar mixture of LiCl and MgCl_2_ (*ca.* 100 equiv. each) in CD_2_Cl_2_, orange solids corresponding to 2·MgCl_2_ precipitated out with no proton signals of the receptor appearing in the ^1^H NMR spectrum of the residual solvent phase (Fig. S21†). Adding methanol (10% by volume relative to CD_2_Cl_2_) to the sample caused the precipitates to dissolve and produced a spectrum analogous to that of 2·LiCl (Fig. S21†). The reversal in selectivity seen for receptor 2 from MgCl_2_ to LiCl upon moving to a more polar medium is rationalized in terms of MgCl_2_ being more strongly solvated than LiCl by methanol. Similarly, the apparent high selectivity of receptor 2 for CaBr_2_ over LiBr in CD_2_Cl_2_ was also reversed when methanol (5% by volume) was added to a CD_2_Cl_2_ solution containing precipitated 2·CaBr_2_ (Fig. S22†).

The solvation effects on the receptor selectivity were further supported by two-phase liquid–liquid extraction experiments using CD_2_Cl_2_ as the organic receiving phase and an aqueous D_2_O solution as the lithium salt source. For instance, when CD_2_Cl_2_ solutions containing precipitates of the respective 2·MgCl_2_ and 2·CaBr_2_ complexes were contacted with aqueous solutions of LiCl (10 M) and LiBr (13 M), respectively, complete dissolution ensued (Fig. 5 and S23†). The ^1^H NMR spectra of the organic phases exhibited proton signals consistent with those of the LiCl and LiBr complexes, respectively (Fig. 5 and S23†). These findings lead us to suggest that MgCl_2_ and CaBr_2_ are released from receptor 2 in the organic phase into the aqueous phases while LiCl and LiBr initially present in the aqueous phase form complexes with receptor 2 that are soluble in the organic phase. This permits the selective extraction of these two lithium salts from an aqueous source phase into a CD_2_Cl_2_ receiving phase. When the resulting organic phases containing the respective LiCl and LiBr complexes of receptor 2 were further contacted with ion-free D_2_O, release of LiCl and LiBr into the aqueous D_2_O phase occurs. This produces receptor 2 in its ion-free form (Fig. 5 and S23†).

In order to obtain further insights into the ability of receptor 2 to extract the above metal chloride and bromide salts from an aqueous phase, we carried out liquid–liquid extraction experiments using CD_2_Cl_2_/D_2_O. For instance, when an CD_2_Cl_2_ layer containing receptor 2 (3 mM) was contacted with aqueous solution layers containing excess LiCl, NaCl, KCl, MgCl_2_, and CaCl_2_, respectively, only in the case of LiCl were chemical shift changes seen in the ^1^H NMR spectrum of the organic layer consistent with effective extraction (Fig. S24†). The resulting ^1^H NMR spectrum was almost identical to that seen upon complexation of receptor 2 with LiCl (*cf.* Fig. S19†). This finding was taken as evidence that receptor 2 is able to extract LiCl with high selectivity from an aqueous source phase into a CD_2_Cl_2_ organic layer. Upon exposure of a CD_2_Cl_2_ solution of receptor 2 (3 mM) to aqueous solutions containing various respective bromide salts, as above, only the lithium salt (LiBr) induced chemical shift changes attributable to LiBr complex formation (Fig. S25†).

To evaluate further the extraction capacity of receptor 2 for LiCl and LiBr, we determined the percentages of the receptor loaded with LiCl and LiBr in the CD_2_Cl_2_ organic phase after contacting with aqueous solutions containing different concentrations of LiCl and LiBr, respectively. When receptor 2 was contacted with an aqueous D_2_O solution containing 5 M of LiCl, two sets of proton signals were visible in the ^1^H NMR spectrum of the organic phase (Fig. S26†). Based on integrations, *ca.* 24% of the receptor was presumed to participate in the LiCl extraction (Fig. S26†). In contrast, the LiCl loading percentage of the receptor from an aqueous solution containing 10 M of LiCl was calculated to be *ca.* ≈ 100% (Fig. S26†). Further evidence for the ability of receptor 2 to extract LiCl came from a high resolution ESI mass spectrometric analysis. A major peak at *m*/*z* = 795.4105, a value corresponding to [M + Li]^+^, was seen (Fig. S27†). On the other hand, the corresponding receptor loading levels for LiBr were estimated to be *ca.* (70 ± 10)% and *ca.* 100% when a CD_2_Cl_2_ solution containing receptor 2 (3 mM) was contacted with aqueous solutions containing 10 M and 15 M concentrations of LiBr, respectively (Fig. S28†). These findings stand in sharp contrast to what was seen with receptor 1 that fails to extract LiCl or LiBr under the same LLE conditions (Fig. S29 and S30†). Both the LiCl and LiBr complexes of receptor 2 could be separated off and washed with D_2_O to release the bound salts into an aqueous D_2_O layer (Fig. 5 and S23†) while freeing up receptor 2 for possible reuse.

The extraction efficiency of receptor 2 for LiCl and LiBr could be improved by employing relatively polar nitrobenzene-*d*_5_ as the organic receiving phase instead of CD_2_Cl_2_. For instance, after a nitrobenzene-*d*_5_ solution containing receptor 2 was contacted with an aqueous source phase containing an excess of LiCl, NaCl, KCl, MgCl_2_, and CaCl_2_, it was found that 100% of the receptor in the organic phase existed in the form of the LiCl complex (Fig. S31†). In the case of NaCl and KCl, less than 30% of the receptor was loaded with these salts in the organic phase while no evidence for MgCl_2_ and CaCl_2_ extraction by receptor 2 was found (Fig. S31†). In contrast, when a nitrobenzene-*d*_5_ organic layer of receptor 2 (3 mM) was contacted with an aqueous layer containing all five test chloride anion salts (LiCl, NaCl, KCl, MgCl_2_, and CaCl_2_) at a concentration of ≈5.0 M each, the resulting ^1^H NMR spectrum of the nitrobenzene layer was consistent with that recorded after receptor 2 was treated with LiCl only (Fig. S31†). This finding was taken as evidence that receptor 2 is capable of extracting LiCl with high selectivity from a mixed salt aqueous solution. In analogy to what was seen in the case of the metal chloride salts, receptor 2 was found to extract LiBr selectively from an aqueous solution over other test bromide salts. For instance, upon subjecting the nitrobenzene-*d*_5_ layer containing receptor 2 to contact with a D_2_O layer containing an excess amount of LiBr, NaBr, KBr, MgBr_2_, and CaBr_2_, respectively, only LiBr gave rise to a ^1^H NMR spectrum of the organic layer consistent with formation of an ion pair complex (Fig. S32†).

**Fig. 6:**
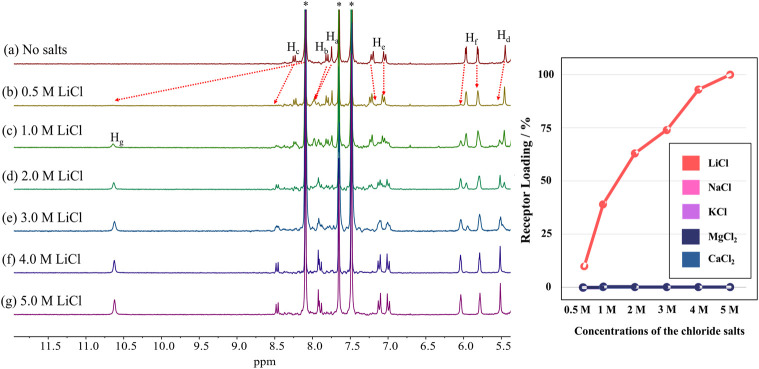
Left: partial ^1^H NMR spectra of nitrobenzene-*d*_5_ layers of 2 (3 mM) recorded upon exposure to aqueous D_2_O phases at concentrations of 0.0 M, 0.5 M, 1 M, 2 M, 3 M, 4 M, and 5 M of LiCl, respectively. Percentages of the receptor loaded with LiCl are estimated to be <10%, 39%, 63%, 74%, 93% and 100% respectively. The asterisks denote the residual nitrobenzene peak in the NMR solvent. Right: plots showing loading percentages of receptor 2 for the metal chloride salts after liquid–liquid extraction.

We also examined the capacity of receptor 2 to extract LiCl and LiBr into nitrobenzene-*d*_5_ from an aqueous D_2_O solution containing various concentrations of LiCl and LiBr, respectively. For instance, when a nitrobenzene-*d*_5_ phase containing receptor 2 (3 mM) was contacted with aqueous D_2_O layers containing 0.5–5.0 M of LiCl, the resulting ^1^H NMR spectra of the nitrobenzene-*d*_5_ phase exhibited two distinguishable sets of proton signals that could be assigned to the ion-free form and the LiCl complex of receptor 2, respectively. The LiCl loading percentages of the receptor in the organic layer were determined to be <10%, 39%, 63%, 74%, 93%, and 100% when the nitrobenzene-*d*_5_ phases containing the receptor were contacted with D_2_O solutions containing 0.5 M, 1.0 M, 2.0 M, 3.0 M, 4.0 M, and 5.0 M of LiCl, respectively (Fig. 6). The LiCl loading levels for receptor 2 were compared to those achieved by receptor 1. For instance, the LiCl loading percentage of receptor 1 from a 10 M LiCl aqueous solution was 15%.^39^ In the case of LiBr, when nitrobenzene-*d*_5_ solutions of receptor 2 were treated with D_2_O aqueous solutions containing LiBr at concentrations of 3.0 M, 5.0 M, and 8.0 M, respectively, 21%, 53%, and 100% of the receptor molecules formed a LiBr complex within the organic phase (Fig. S33†).

**Fig. 7:**
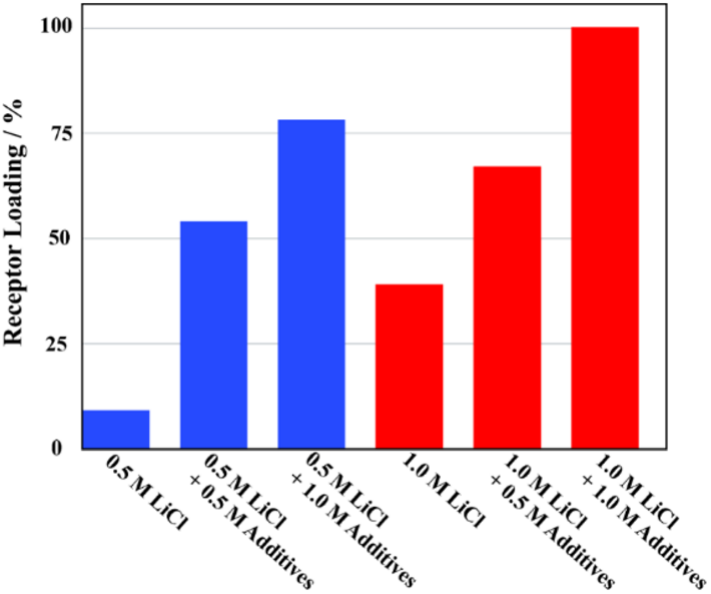
LiCl loading percentages for receptor 2 after nitrobenzene-*d*_5_ organic phases were contacted with aqueous D_2_O phases containing 0.5 M and 1.0 M LiCl in the absence and presence of (concurrently) NaCl, KCl, MgCl_2_, and CaCl_2_ at concentrations of 0.5 M and 1.0 M in each additive.

The ability of receptor 2 to extract LiCl selectively was evaluated under liquid–liquid extraction conditions using aqueous solutions containing other potentially competitive chloride salts including NaCl, KCl, MgCl_2_, and CaCl_2_. In the case of the individual salts, receptor 2 failed to extract chloride salts other than LiCl under otherwise identical LLE conditions when aqueous solutions containing 5.0 M of the metal cation chloride anion salts in question were tested (Fig. 6). This selectivity was retained when mixtures of salts were tested. However, unexpectedly, the extraction efficiency of the receptor for LiCl was boosted in the presence of the other test chloride salts. For instance, the proton integration ratios corresponding to the 2·LiCl complex in the ^1^H NMR spectra of the nitrobenzene-*d*_5_ phases increased as the concentration of the other chloride salts increased (Fig. 7 and S34†). By way of a specific example, when a nitrobenzene-*d*_5_ phase containing receptor 2 (3 mM) was contacted with an aqueous solution containing 0.5 M of LiCl in the absence of the other salts, <10% of the receptor was loaded with LiCl in the organic layer (Fig. 7 and S34†). By contrast, the LiCl loading percentages of receptor 2 were enhanced up to 54% and 78% when the aqueous LiCl source phase (0.5 M) consisted of a mixture of NaCl, KCl, MgCl_2_, and CaCl_2_ at concentrations of 0.5 M each and 1.0 M each, respectively. This loading increased to nearly 100% when each of the salts, including LiCl, was present at 1.0 M (Fig. 7 and S32†). Similar results were observed in liquid–liquid extraction studies involving dichloromethane-*d*_2_ as the receiving phase. For instance, upon contacting an organic dichloromethane-*d*_2_ phase containing receptor 2 with an aqueous D_2_O phase containing 5.0 M LiCl, 24% of the receptor in the organic phase was loaded with LiCl. In the presence of excess NaCl and NaBr along with 5.0 M LiCl in the aqueous phase, the LiCl loading percentages improved to 36% and 42%, respectively (Fig. S35 and S36†). These findings, which appear analogous to a classic salting-out effect, lead us to suggest that the ion extraction efficiency of other LLE extractants could be improved by employing aqueous source phases containing non-competing salts that are intrinsically insoluble in the organic receiving phase.

## Conclusions

The new ion pair receptor 2 reported here acts as a selective extractant for LiCl and LiBr under solid–liquid and liquid–liquid extraction conditions. Ion pair receptor 2 is composed of a phenanthroline unit of a cation binding site connected *via* ether linkages to a calix[4]pyrrole anion binding site. In dichloromethane-*d*_2_, receptor 2 proved able to complex LiCl, LiBr, MgCl_2_, MgBr_2_, and CaBr_2_. When treated with the salts in question (*i.e.*, LiX, NaX, KX, MgX_2_, and CaX_2_ where X = Cl or Br), receptor 2 forms insoluble complexes selectively with MgCl_2_ and CaBr_2_. This operational selectivity is reversed in more polar protic solvents, such as water or methanol. For instance, upon the *in situ* addition of methanol to CD_2_Cl_2_ solutions containing the presumed MgCl_2_ and CaBr_2_ complexes of receptor 2 along with the other chloride and bromide salts, the bound MgCl_2_ and CaBr_2_ are released from the receptor giving rise to the corresponding LiCl and LiBr complexes. This finding is ascribed to the Mg^2+^ and Ca^2+^ cations being strongly solvated by polar protic media as compared to the Li^+^ cation. In a moderately polar medium consisting of 10% CH_3_OH in CDCl_3_, receptor 2 binds LiCl and LiBr ion with significantly higher affinity and selectivity than it does other competitive chloride and bromide salts. Receptor 2 was also found to able to extract LiCl and LiBr efficiently into an organic phase from aqueous sources under two-phase liquid–liquid extraction conditions. This efficiency could be further boosted by adding other chloride salts, including NaCl, KCl, MgCl_2_, and CaCl_2_ to the aqueous source phase. We thus suggest that receptor 2 may have a role to play in lithium cation separation scenarios.

## Author contributions

SKK and JLS conceived and supervised the project. NJH synthesized the compounds. NJH and JHO performed ion binding studies using ^1^H NMR spectroscopy. NJH and KL carried out the X-ray diffraction analysis. AL and QH carried out DFT calculations. SKK and JLS wrote the manuscript. All authors contributed to the editing of the manuscript.

## Conflicts of interest

There are no conflicts to declare.

## Supplementary Material

SC-015-D4SC03760J-s001

SC-015-D4SC03760J-s002

## Data Availability

All data, including synthetic details, ^1^H NMR spectroscopic analyses, DFT calculations, and a single crystal X-ray diffraction analysis of 2·LiBr (CCDC 2356046), are available in the ESI.† All data are included either in the main text, the ESI,† or (X-ray work only) uploaded with the Cambridge Crystallographic Data Centre.
