# Correction: Ion pair extractant selective for LiCl and LiBr

**DOI:** 10.1039/d4sc90238f

**Published:** 2024-12-04

**Authors:** Nam Jung Heo, Ju Hyun Oh, Aimin Li, Kyounghoon Lee, Qing He, Jonathan L. Sessler, Sung Kuk Kim

**Affiliations:** a Department of Chemistry, Research Institute of Natural Sciences, Gyeongsang National University Jinju 52828 Korea sungkukkim@gnu.ac.kr; b State Key Laboratory of Chemo/Biosensing and Chemometrics, College of Chemistry and Chemical Engineering, Hunan University Changsha 410082 P. R. China; c Department of Chemistry Education, Research Institute of Natural Sciences, Gyeongsang National University Jinju 52828 Korea; d Department of Chemistry, The University of Texas at Austin 105 E. 24th Street-Stop A5300 Austin Texas 78712-1224 USA sessler@cm.utexas.edu

## Abstract

Correction for ‘Ion pair extractant selective for LiCl and LiBr’ by Nam Jung Heo *et al.*, *Chem. Sci.*, 2024, **15**, 13958–13965, https://doi.org/10.1039/D4SC03760J.

The authors regret that funding details were incorrect in the acknowledgements section of the original article. The corrected acknowledgements section for this article is shown below.

**Acknowledgements**

This work was supported by the National Research Foundation of Korea (NRF) funded by the Korea Government (MIST) (NRF 2021R1A2C1012140 to S. K. K.). The work in Austin was supported by the Department of Energy Office of Basic Energy Sciences (grant no. DE-SC0024393 to J. L. S.). Further support from the Robert A. Welch Foundation (F-0018 to J. L. S.) is also acknowledged.

The Royal Society of Chemistry apologises for these errors and any consequent inconvenience to authors and readers.

