# Supplementary material for: Ion pair extractant selective for LiCl and LiBr
Source: Chem Sci. 2024 Aug 12;15(34):13958–65. doi: 10.1039/d4sc03760j (PMC11317791; doi:10.1039/d4sc03760j)
Supplement: SC-015-D4SC03760J-s001 [file SC-015-D4SC03760J-s001.pdf]

Supplementary Information (SI) for Chemical Science.
This journal is © The Royal Society of Chemistry 2024

Supporting Information for:

Ion Pair Extractant Selective for LiCl and LiBr

Nam Jung Heo,[a] Ju Hyun Oh,[a] Aimin Li,[b] Kyounghoon Lee,[c] Qing He,[b] Jonathan L. Sessler,[*,d] and Sung Kuk Kim[*,a]

[a]Department of Chemistry and Research Institute of Natural Sciences, Gyeongsang National University, Jinju, 52828, Korea, [b]State Key Laboratory of Chemo/Biosensing and Chemometrics, College of Chemistry and Chemical Engineering, Hunan University, Ghangsha 410082, P. R. China, [c]Department of Chemistry Education and Research Institute of Natural Sciences, Gyeongsang National University, Jinju, 52828, Korea and [d]Department of Chemistry, The University of Texas at Austin, 105 E. 24th Street-Stop A5300, Austin, Texas 78712-1224, USA

1. General experimental and synthetic details S2-S3
2. Determination of association constants S4
3. ^{1}H NMR spectroscopic analyses of the binding and extraction of ions and ion pairs by receptor **2** S5-S38
4. NMR spectra and HRMS data for receptor **2** S39-S41
5. X-ray experimental for receptor **1** S42-S43
6. Geometrical coordinates of the optimized structures S44-S66
7. References S67

General experimental and synthetic details

Solvents and reagents used for the synthetic work were purchased from Aldrich, TCI, or Alfa Aesar and used without further purification. NMR spectra were recorded on a Bruker Advance-300 MHz instrument. NMR spectra were referenced to residual solvent peaks. The spectroscopic solvents were purchased from either Cambridge Isotope Laboratories or Aldrich. Quadrupole time-of-flight (QTOF) mass spectra were recorded on a VG ZAB-2E instrument or a VG AutoSpec apparatus. TLC analyses were carried out using Sorbent Technologies silica gel (200 mm) sheets. Column chromatography was performed on Sorbent Technologies silica gel 60 (40–63 mm). Theoretical calculations evaluating the stability of various putative structures were carried out with the Gaussian 09 suite[1] of programs using the X3LYP density functional.[2] Structural optimization was performed using a 6–31G* basis set. Complexation energies were corrected for basis set superposition error (BSSE) using the counterpoise correction method.[3,4]

Liquid-liquid extraction of the salts using the ion pair receptors

To 5 mL vials containing 2 mL of dichloromethane-d_2 or nitrobenzene-d_5 solutions of the receptors (3 mM) vials were added 2 mL of aqueous solutions of the salts in question. The resulting two-phase solutions were vigorously shaken for 10 min and then allowed to stand for 1~2 hours until the two phases are clearly separated. The aqueous phase was removed, and 1 ml of the organic phase was transferred to an NMR tubes The organic solution was then monitored by ^{1}H NMR spectroscopy. The integrations corresponding to the ion free forms and the salt complex forms of the receptor, respectively, were compared to determine the loading percentages of the receptor with salts.

Receptor 2: Compound **3** (1.36 g, 2.48 mmol),[5] compound **4** (1.45 g, 2.48 mmol)[6] and K_2CO_3 (1.71 g, 12.37 mmol) were dissolved in 80 mL of acetonitrile and heated to reflux under a nitrogen atmosphere. After the resulting reaction mixture was stirred at reflux for 24 hours, the volatiles were removed *in vacuo*. To the resulting brown solid, CH_2Cl_2 (100 mL) was added and the organic layer was separated off and washed three times with 200 mL of water. The organic layer was dried over anhydrous $MgSO_4$ and the solvent was evaporated *in vacuo* to give a brown solid. Column chromatography over silica gel (eluent: acetone/dichloromethane (1:70)) gave 0.22 g (11.25% yield) of **2** as a white solid. ^{1}H NMR (300 MHz, chloroform-*d*) δ 8.28 (d, *J* = 8.3 Hz, 2H), 7.88 (d, *J* = 8.3 Hz, 2H), 7.81 (s, 2H), 7.21 – 7.10 (m, 4H), 7.05 – 6.95 (m, 4H), 6.63 (s, 4H), 5.93 – 5.85 (m, 4H), 5.78 (dd, *J* = 3.4, 2.8 Hz, 4H), 5.66 (s, 4H), 1.89 (s, 6H), 1.42 (s, 6H), 1.23 (s, 6H). ^{13}C NMR (75 MHz, chloroform-*d*) δ 157.9, 145.3, 138.5, 137.3, 136.9, 128.3, 126.6, 122.84, 115.6, 104.2, 73.7, 44.0, 35.3, 29.9, 29.5. HRMS (QTOF) *m/z* 788.3839 $[M]^+$ calcd for $C_{52}H_{48}N_6O_2$, found 788.3917.

Determination of association constants for host-guest association/dissociation equilibria that are slow on the NMR time scale using ^{1}H NMR spectral titrations

Equilibrium: $A + B \underset{}{\overset{K_a}{\rightleftharpoons}} AB$

Equilibrium constant: $K_a = \frac{[AB]}{[A][B]} = \frac{[AB]}{(c(A)-[AB])(c(B)-[AB])}$ (1)

c(A) and *c*(B) are the initial concentrations of A and B, and [A], [B] and [AB] are the equilibrium concentrations of the three species.

A and B is in slow exchange with the complex AB on the ^{1}H NMR time scale.

Two signals for one specific proton on A can be seen in the spectrum, corresponding to complexed and uncomplexed forms of A:

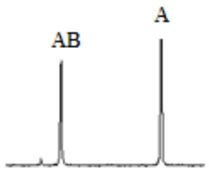

Single-point Methods

K_a is determined from the integrals of complexed and uncomplexed A. If *I*(A) denotes the integral of a signal for one specific proton of A and *I*(AB) the integral for the same proton in the complex, the concentration of AB at equilibrium is shown by eq 2. The equilibrium expression (eq 3) is obtained after substituting into eq (1):

$$[AB]=\frac{I(AB)}{I(A)+I(AB)}c(A) \quad (2)$$

$$K_a = \frac{I(AB)}{I(A)\left(c(B)-\frac{I(AB)}{I(A)+I(AB)}c(A)\right)} \quad (3)$$

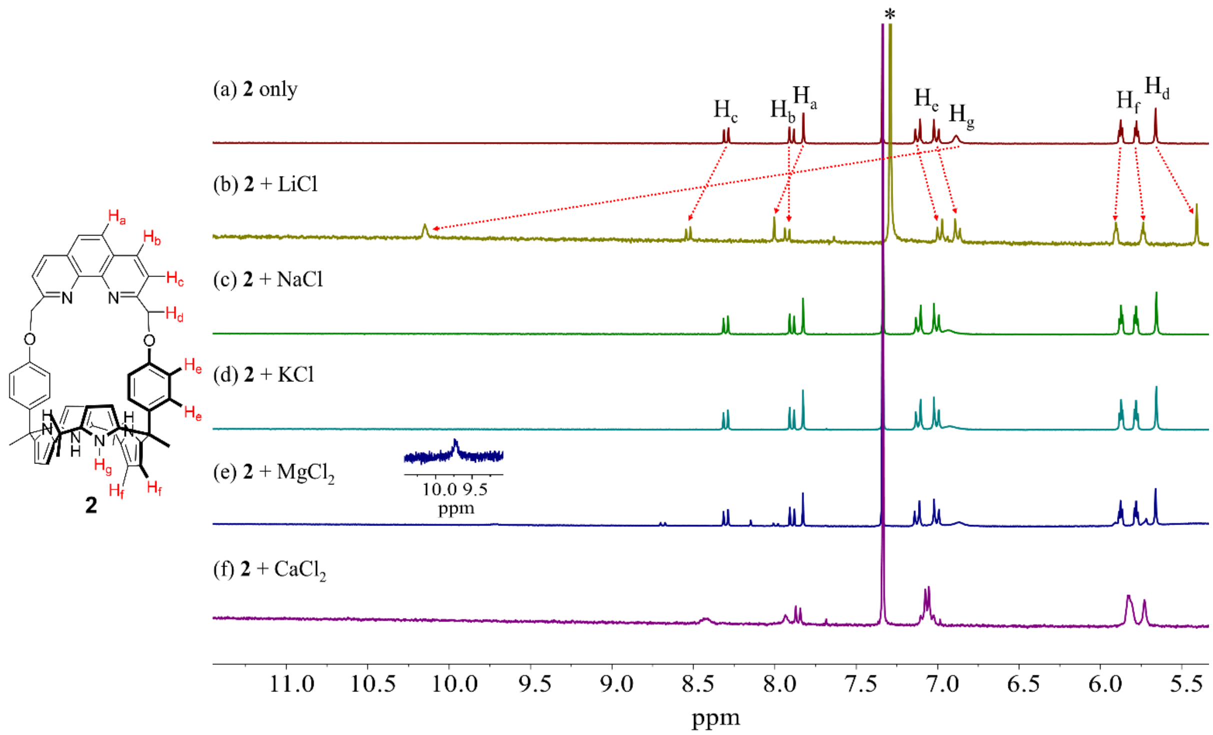

Figure S1. Partial ^{1}H NMR spectra of (a) **2** (3 mM) only, (b) **2** + excess LiCl, (c) **2** + excess NaCl, (d) **2** + excess KCl, (e) **2** + excess $MgCl_2$, and (f) **2** + excess $CaCl_2$ in $CH_3OH/CDCl_3$ (1:9, v/v). The asterisk (*) denotes the residual $CHCl_3$ peak in the NMR solvent.

Upon exposure of receptor **2** to excess LiCl (ca. 100 equiv), the proton signals (H_a, H_b, and H_c) in the ^{1}H NMR spectrum corresponding to the phenanthroline unit underwent noticeable downfield shifts presumably due to the complexation of the lithium cation with the phenanthroline nitrogen atoms. A more significant downfield shift ($\Delta\delta$ = 3.23 ppm) was seen for the NH proton signal of the calix[4]pyrrole unit, a finding attributed to the formation of hydrogen bonds between the NH protons and the chloride anion. These chemical shift changes are distinct from those taking place when either the lithium cation (as its perchlorate (ClO_4^-) salt) or the chloride anion (as its tetrabutylammonium (TBA^+) salt) was added to receptor **2** (*vide infra*). These findings led us to conclude that receptor **2** forms an ion pair complex with LiCl. By contrast, no appreciable chemical shift changes were observed in the ^{1}H NMR spectra in the presence of NaCl or KCl, a finding interpreted in terms of receptor **2** failing to complex these salts. In contrast, upon the addition of excess $MgCl_2$ (ca. 100 equiv) to receptor **2**, two distinguishable sets of proton signals appeared that corresponded to the ion-free receptor and its $MgCl_2$ complex, respectively. In analogy to what was seen with LiCl, Mg^{2+} is presumed to

be bound to the phenanthroline unit while one of two Cl^- anions is bound to the calix[4]pyrrole moiety via hydrogen bonds (Figure 2). Based on the integration ratios of the proton signals of the ion-free receptor and the $MgCl_2$ complex, roughly 16% of receptor **2** forms a complex with $MgCl_2$ under these conditions ($CH_3OH/CDCl_3$; 1:9, v/v). Distinct chemical shift changes were also observed in the ^1H NMR spectrum when receptor **2** was treated with $CaCl_2$ in $CH_3OH/CDCl_3$ (1:9, v/v). For instance, the proton peaks corresponding to the phenanthroline unit experienced relatively small downfield shifts as compared to LiCl and $MgCl_2$ while the pyrrolic NH proton signal disappeared. A follow up ^1H NMR spectral titration with $CaCl_2$ revealed that the pyrrolic NH proton signal of receptor **2** was only slightly downfield shifted upon addition of up to 5.6 equiv of $CaCl_2$ before it disappeared in the presence of > 9.6 equiv of $CaCl_2$ (Figure S2). These ^1H NMR spectral changes are consistent with the Ca^{2+} cation only, and not the Cl^- anion, being complexed within receptor **2**.

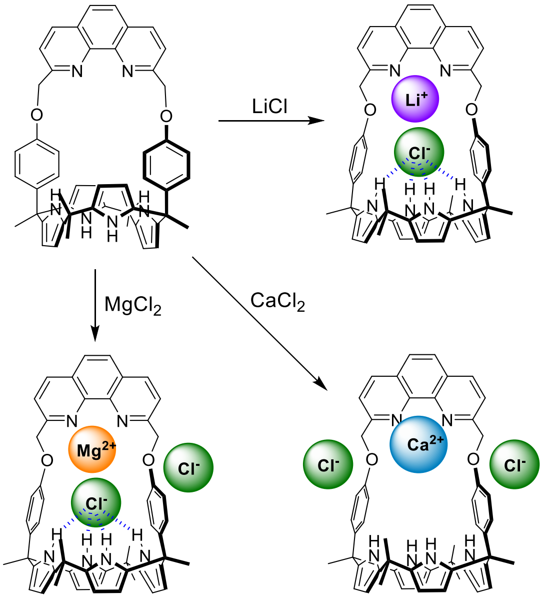

Figure S2. Binding modes of receptor **2** for LiCl, $MgCl_2$, and $CaCl_2$ in 10% CH_3OH in $CDCl_3$ as inferred from ^{1}H NMR spectroscopic analyses.

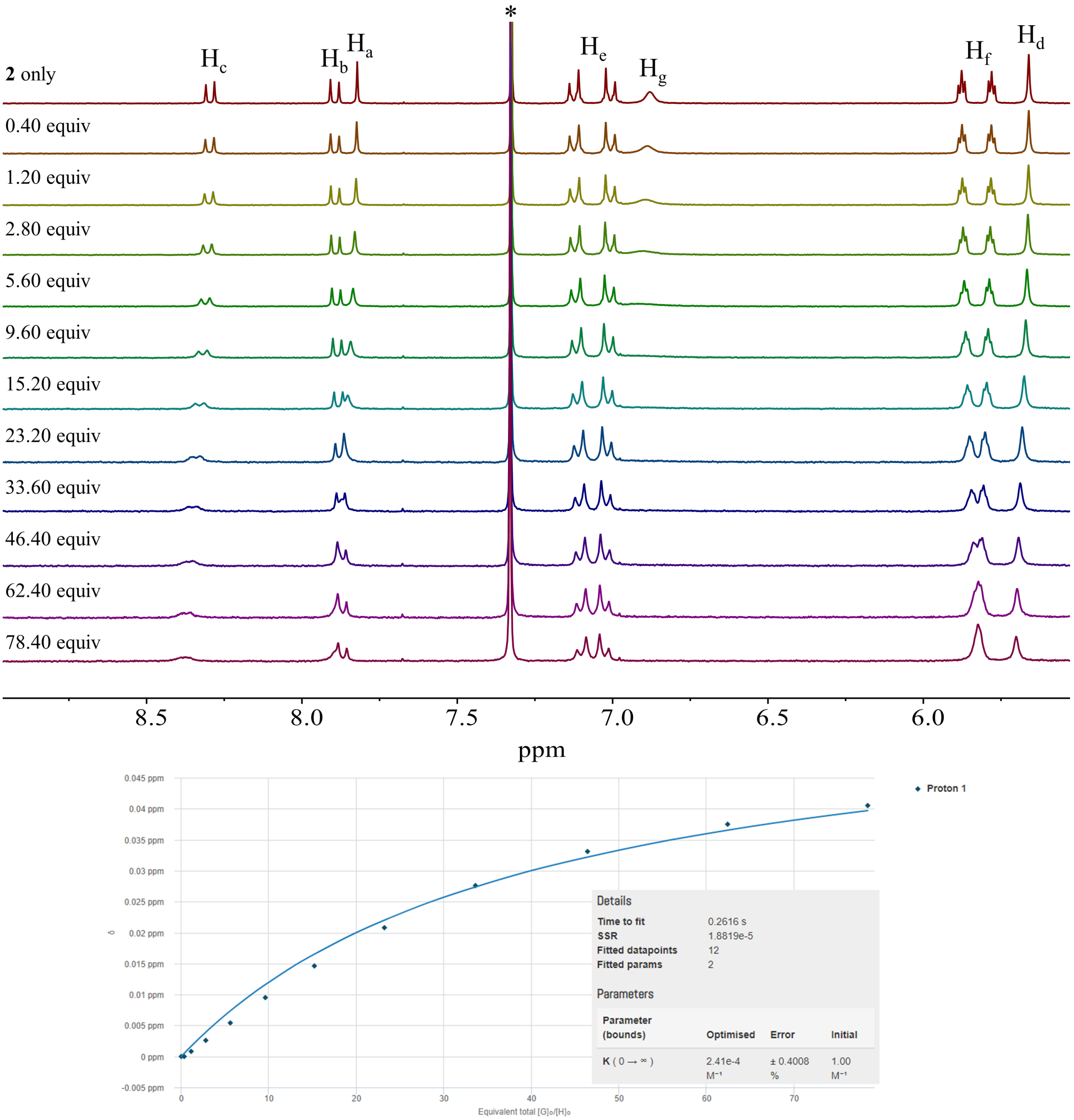

Figure S3. ^{1}H NMR spectra recorded during the titration of **2** (3 mM) with $CaCl_2$ in $CH_3OH/CDCl_3$ (1:9, v/v). The asterisk (*) denotes the residual $CHCl_3$ peak in the NMR solvent.

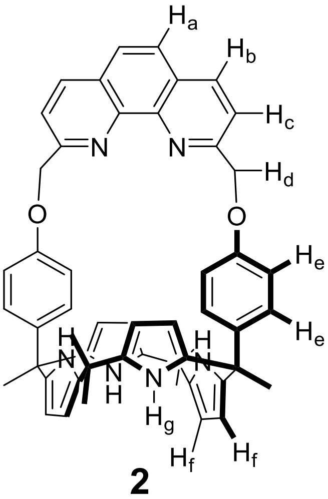

2

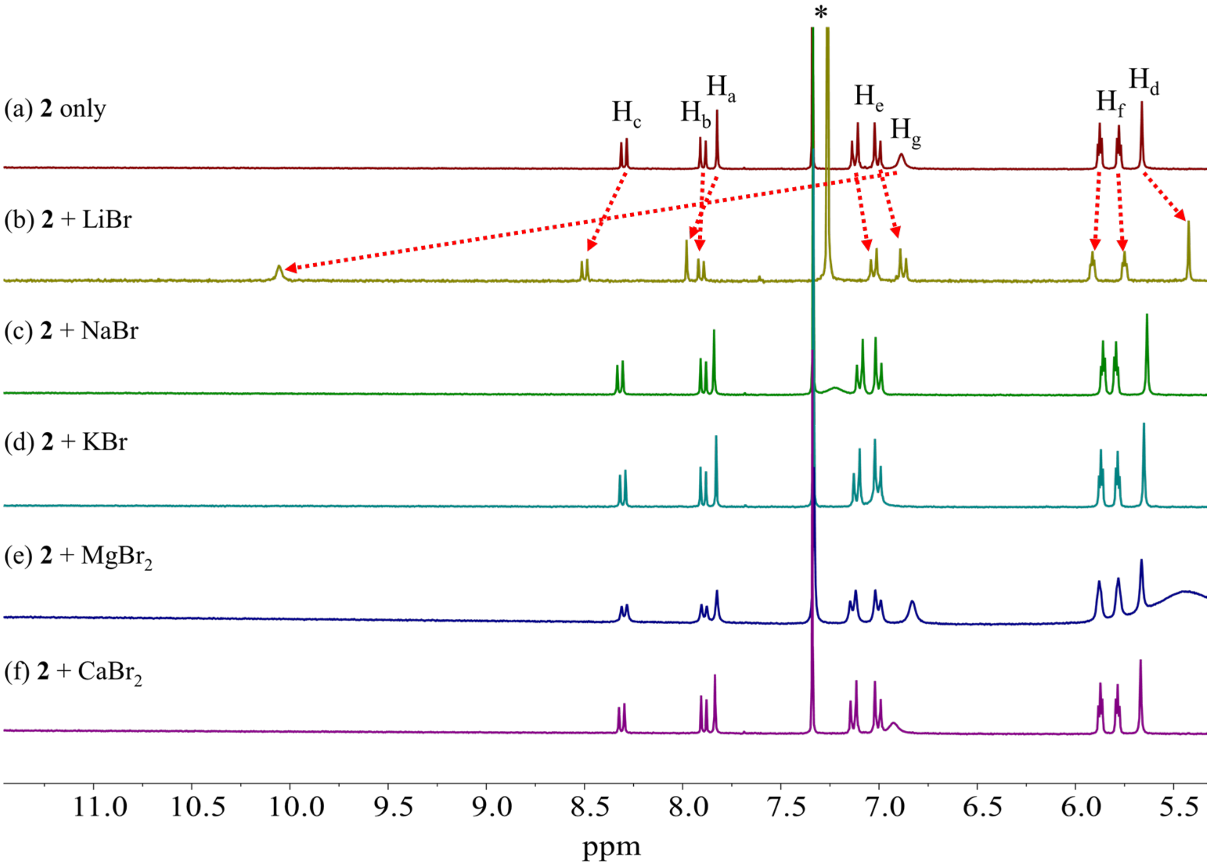

Figure S4. Partial ^{1}H NMR spectra of (a) **2** (3 mM) only, (b) **2** + excess LiBr, (c) **2** + excess NaBr, (d) **2** + excess KBr, (e) **2** + excess $MgBr_2$, and (f) **2** + excess $CaBr_2$ in $CH_3OH/CDCl_3$ (1:9, v/v). The asterisk (*) denotes the residual $CHCl_3$ peak in the NMR solvent.

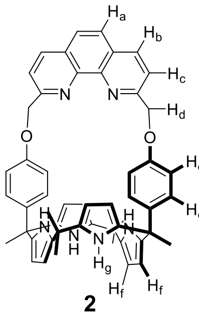

2

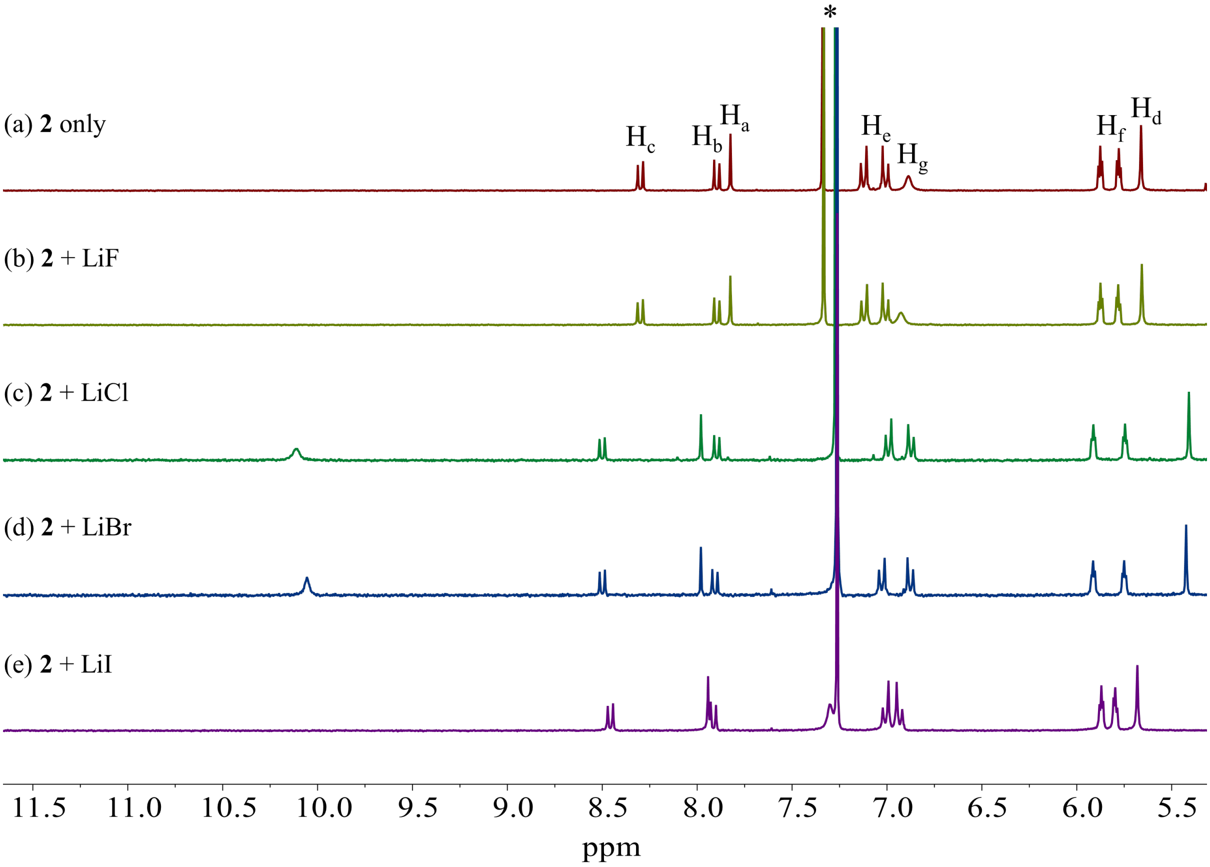

Figure S5. Partial ^{1}H NMR spectra of (a) **2** (3 mM) only, (b) **2** + excess LiF, (c) **2** + excess LiCl, (d) **2** + excess LiBr, and (e) **2** + excess LiI in $CH_3OH/CDCl_3$ (1:9, v/v). The asterisk (*) denotes the residual $CHCl_3$ peak in the NMR solvent.

When exposed to LiI, the pyrrolic NH proton of receptor **2** underwent a very small downfield shift as compared to treatment with LiCl and LiBr. By contrast, the proton peaks assignable to the phenanthroline CH protons were shifted to lower field in analogy to what was seen upon exposure to LiCl and LiBr. These findings are interpreted in terms of the lithium cation binding to the phenanthroline moiety without the iodide anion being co-complexed by the calix[4]pyrrole subunit.

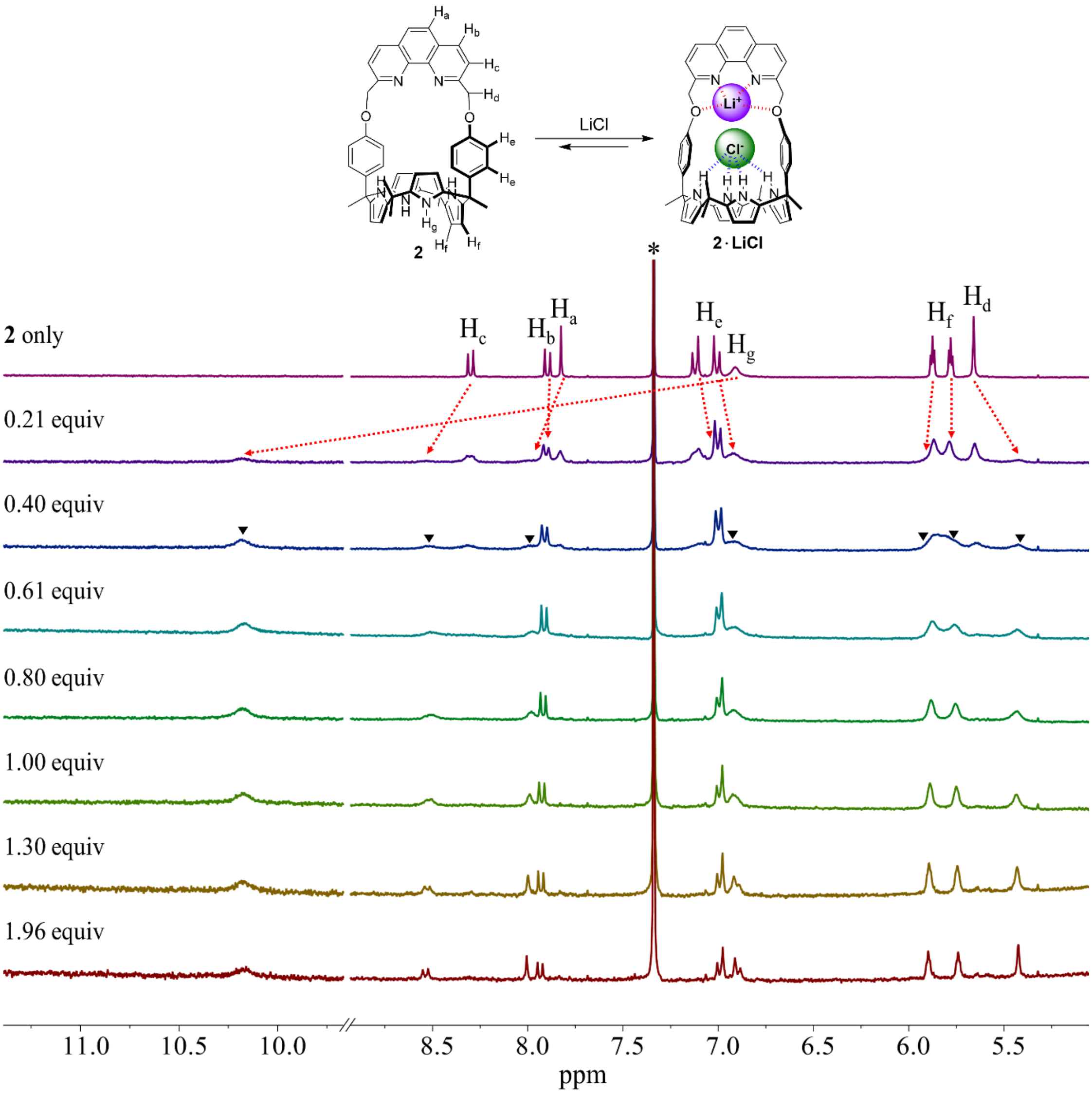

Figure S6. ^{1}H NMR spectra recorded during the titration of **2** (3 mM) with LiCl in $CH_3OH/CDCl_3$ (1:9, v/v). The peaks marked as ▼ represent the proton signals corresponding to the LiCl complex of **2** (**2**•LiCl). The asterisk (*) denotes the residual $CHCl_3$ peak in the NMR solvent.

When receptor **2** was subjected to titration with LiCl in 10% CH_3OH in $CDCl_3$, two distinguishable sets of proton signals emerged in the ^{1}H NMR spectrum before saturation was achieved upon addition of 1.0 equiv of LiCl. These sets of proton signals correspond to the ion-free form of receptor **2** and its LiCl complex form, respectively. These ^{1}H NMR spectral changes support the conclusion that receptor **2** binds LiCl quantitatively with a 1:1 binding stoichiometry via a binding-release equilibrium that is slow on the NMR time scale.

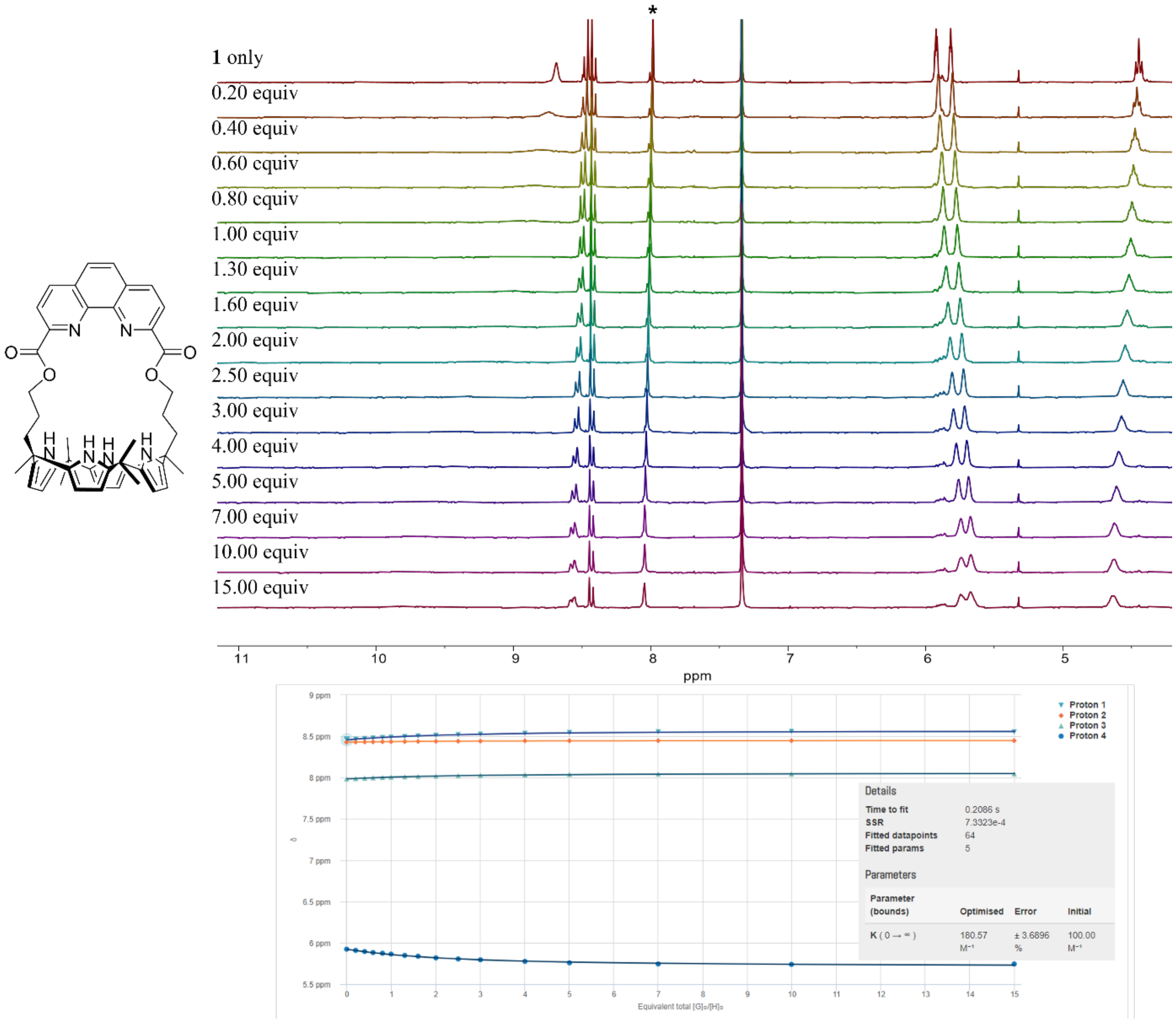

Figure S7. ^{1}H NMR spectra recorded during the titration of **1** (3mM) with LiCl in $CD_3OD/CDCl_3$ (1:9, v/v). The asterisk (*) demotes the residual $CHCl_3$ peak in the NMR solvent.

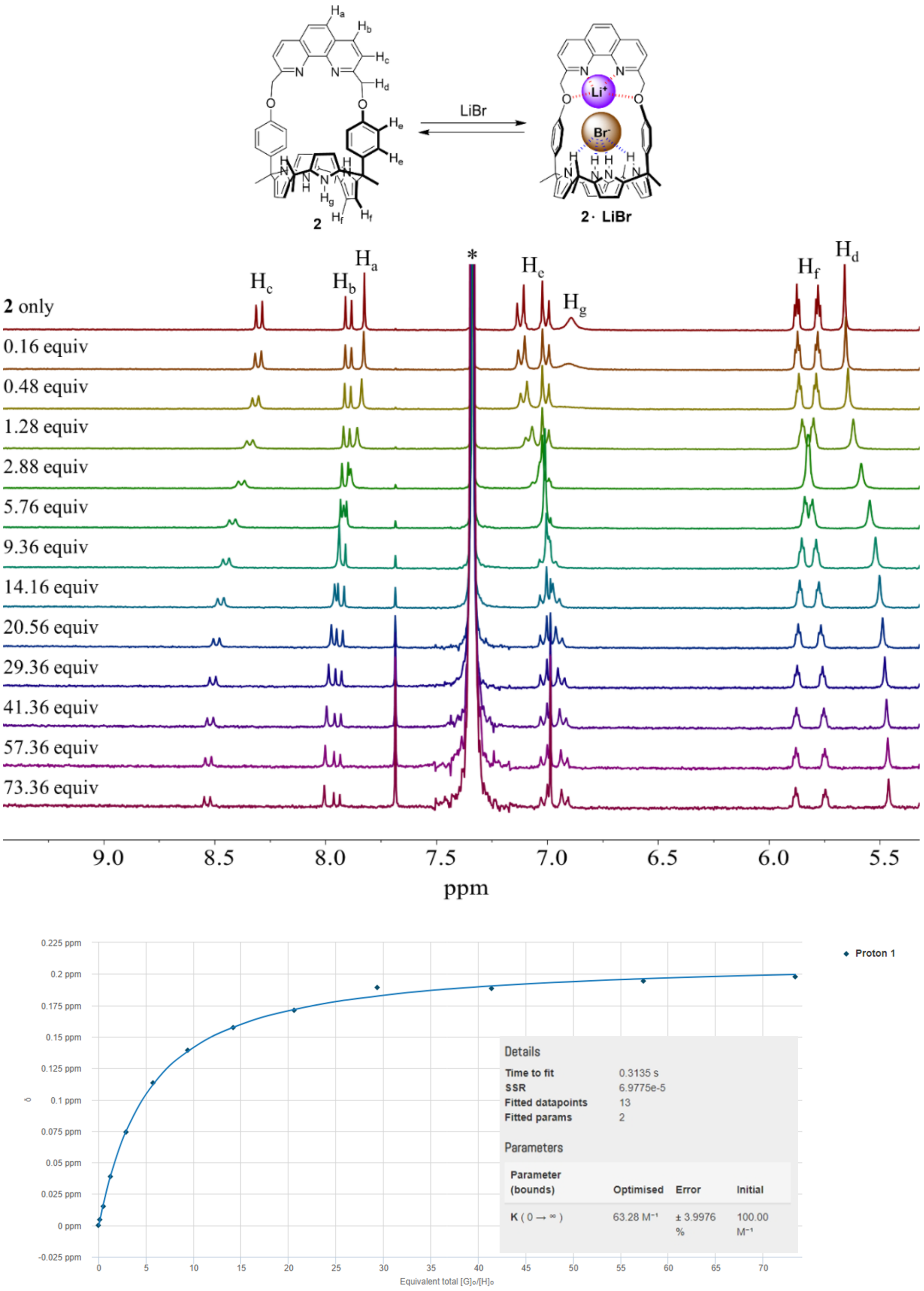

Figure S8. ^{1}H NMR spectra recorded during the titration of **2** (3 mM) with LiBr in $CH_3OH/CDCl_3$ (1:9, v/v). The asterisk (*) denotes the residual $CHCl_3$ peak in the NMR solvent.

Different chemical shift changes in the receptor proton signals took place when receptor **2** was titrated with LiBr in10% CH_3OH in $CDCl_3$. The proton signals of the receptor appeared to be gradually shifted towards either downfield or upfield and saturation was accomplished upon addition of 73 equiv of LiBr. These spectral changes are consistent with receptor **2** binding

LiBr with low affinity relative to LiCl and via an equilibrium process that is fast on the NMR timescale.

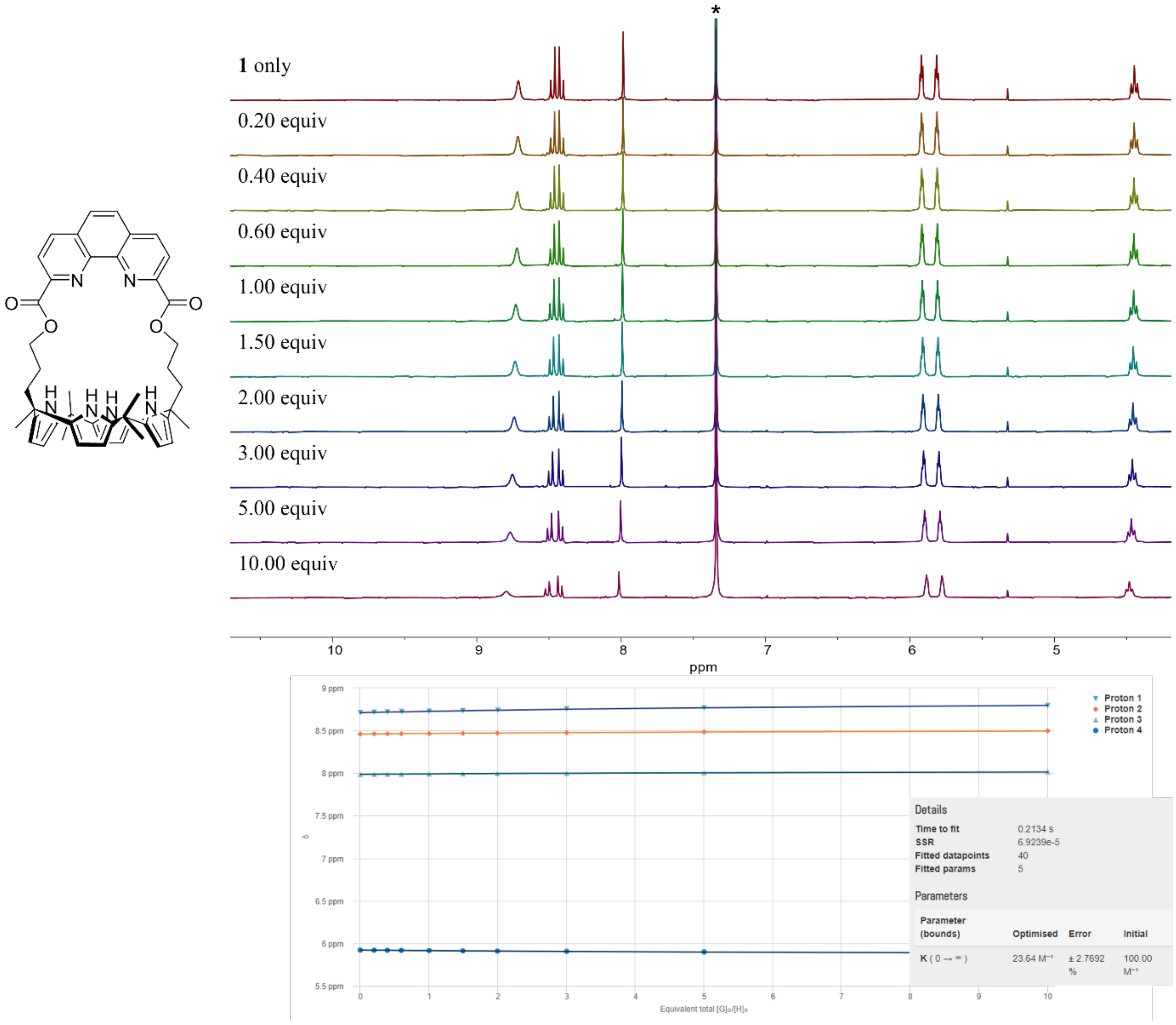

Figure S9. ^{1}H NMR spectra recorded during the titration of **1** (3mM) with LiBr in $CD_3OD/CDCl_3$ (1:9, v/v). The asterisk (*) demotes the residual $CHCl_3$ peak in the NMR solvent.

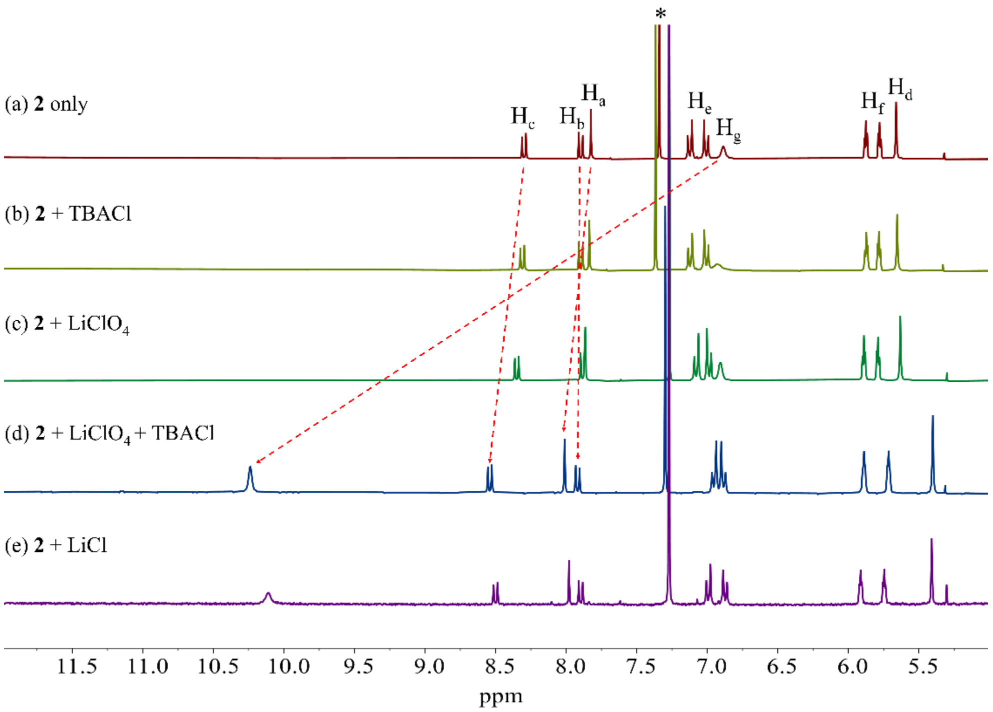

Figure S10. Partial ¹H NMR spectra of (a) **2** (3 mM) only, (b) **2** + excess TBACl, (c) **2** + excess $LiClO_4$, (d) **2** + excess $LiClO_4$ + excess TBACl, and (e) **2** + excess LiCl in $CH_3OH/CDCl_3$ (1:9, v/v). The asterisk (*) denotes the residual $CHCl_3$ peak in the NMR solvent.

The addition of Cl^- and Br^- (as their TBA^+ salts) to receptor **2** in the presence of Li^+ (as its ClO_4^- salt) induced significant chemical shift changes in the receptor proton signals giving rise to ¹H NMR spectra closely analogous to those obtained when receptor **2** was treated with LiCl and LiBr, respectively. We thus conclude that Cl^- and Br^- are bound to the receptor in the presence of the Li^+ cation in 10% CH_3OH in $CDCl_3$ forming LiCl and LiBr ion pair complexes, respectively (Figures S9 and S10). This stands in sharp contrast to what is observed in the absence of a Li^+ cation source.

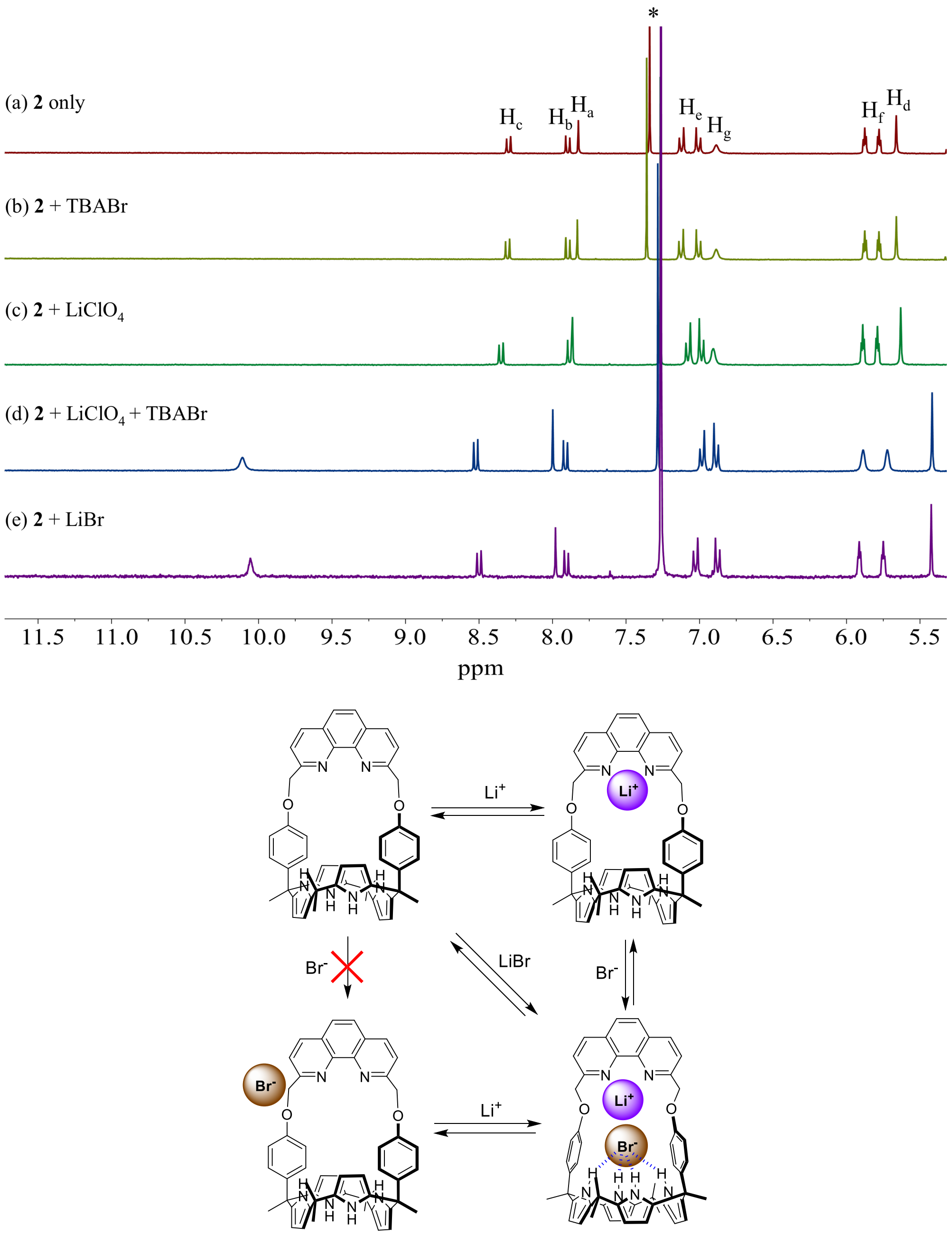

Figure S11. Top: Partial ^{1}H NMR spectra of (a) **2** (3 mM) only, (b) **2** + excess TBABr, (c) **2** + excess $LiClO_4$, (d) **2** + excess $LiClO_4$ + excess TBABr, and (e) **2** + excess LiBr in $CH_3OH/CDCl_3$ (1:9, v/v). The asterisk (*) denotes the residual $CHCl_3$ peak in the NMR solvent. Bottom: Proposed ion binding modes in the presence of Li^+, Br^-, and both Li^+ and Br^-, respectively, in $CH_3OH/CDCl_3$ (1:9, v/v) as inferred from ^{1}H NMR spectroscopic studies.

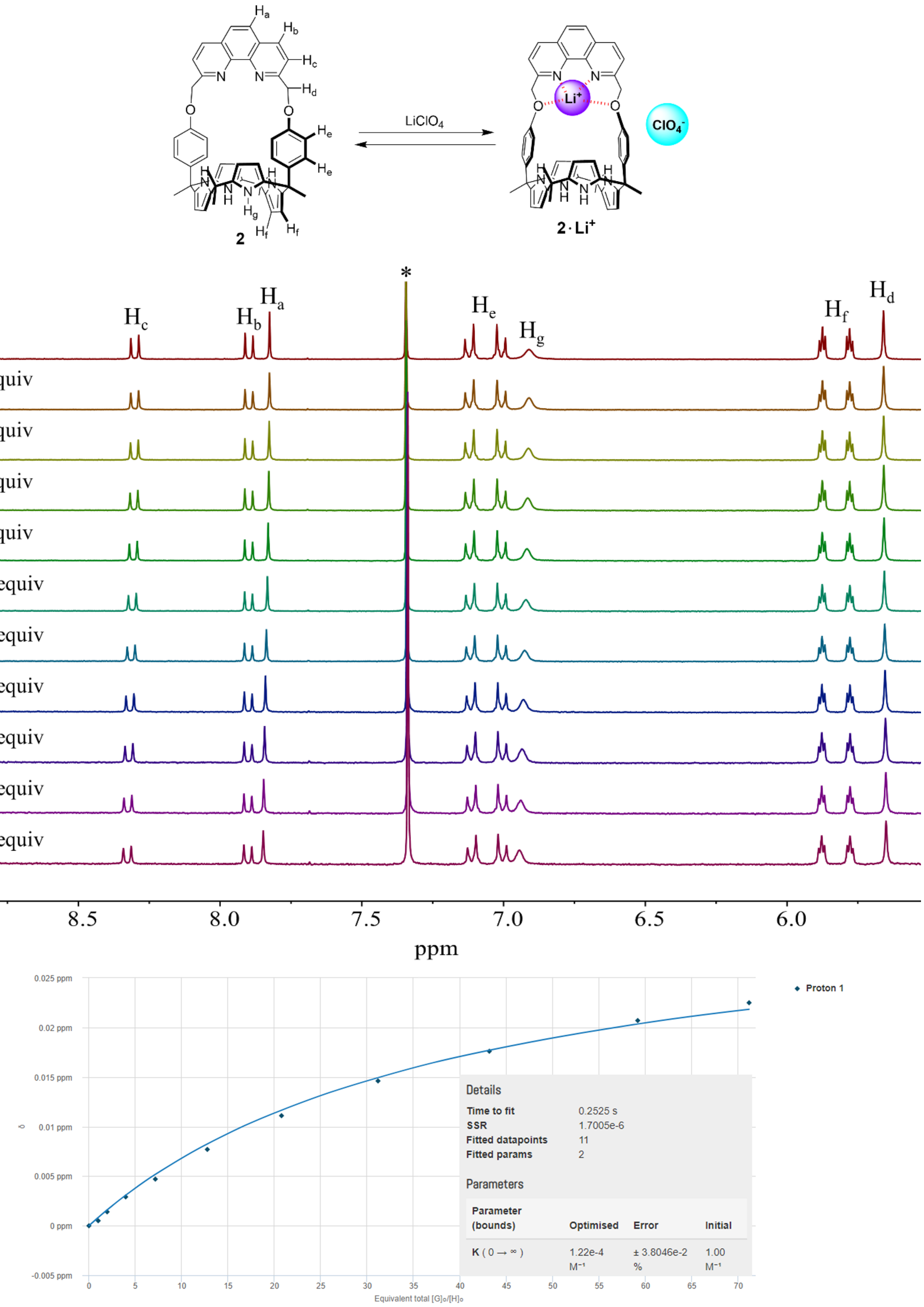

Figure S12. ^{1}H NMR spectra recorded during the titration of **2** (3 mM) with $LiClO_4$ in $CH_3OH/CDCl_3$ (1:9, v/v). The asterisk (*) denotes the residual $CHCl_3$ peak in the NMR solvent.

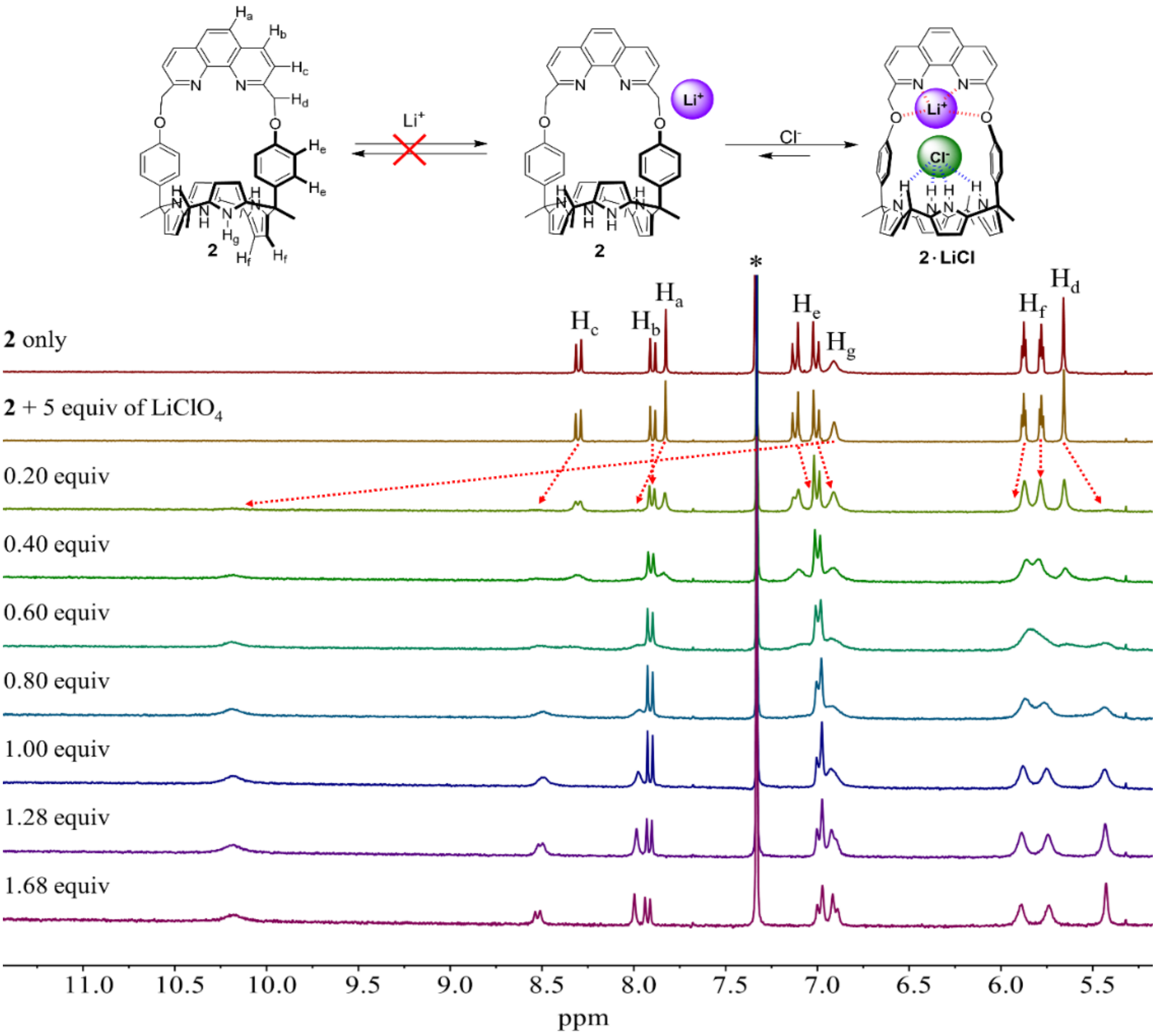

Figure S13. ^{1}H NMR spectra recorded during the titration of **2** with TBACl in the presence of 5.0 equiv of $LiClO_4$ in $CH_3OH/CDCl_3$ (1:9, v/v). The asterisk (*) denotes the residual $CHCl_3$ peak in the NMR solvent.

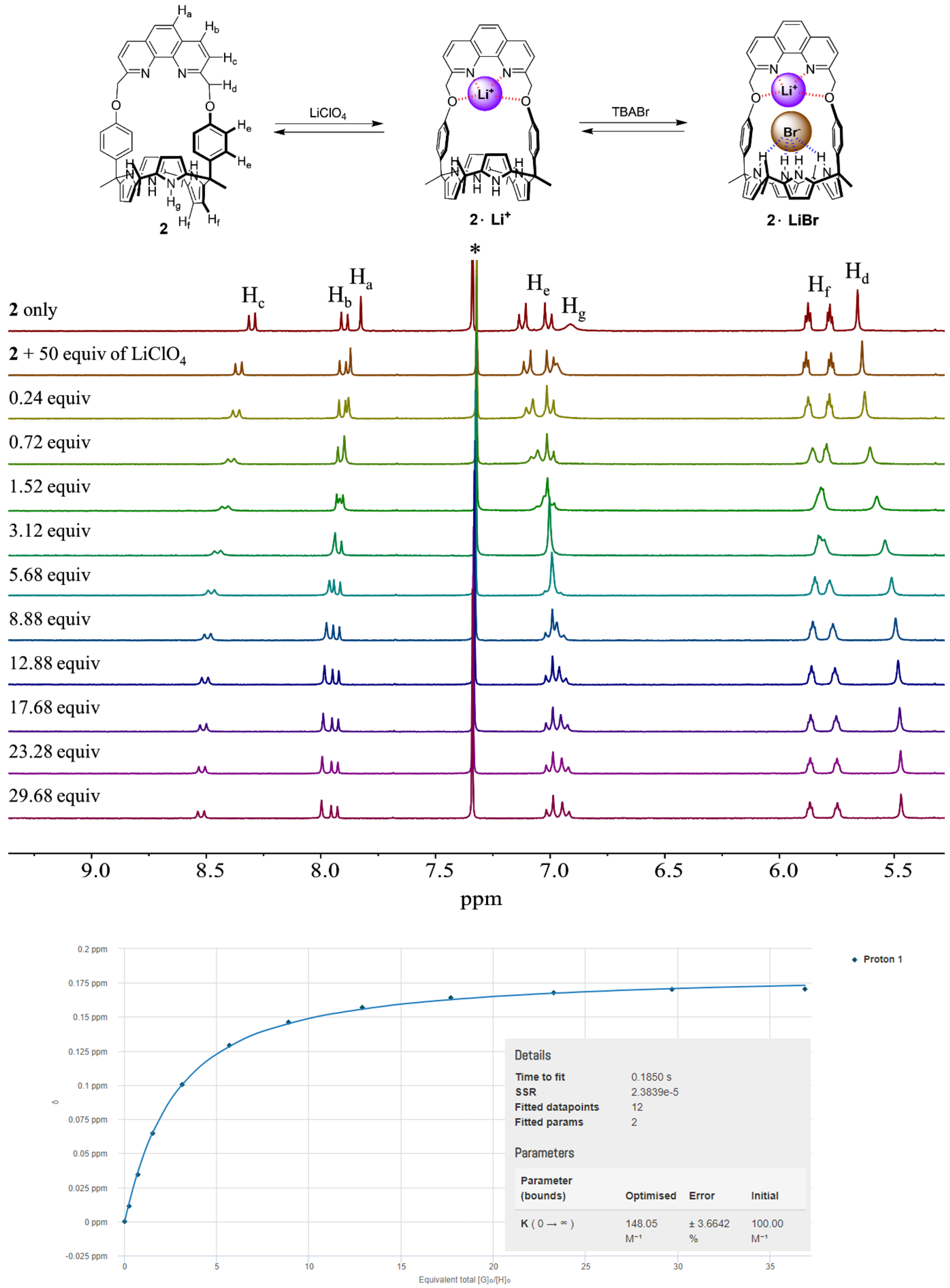

Figure S14. ^{1}H NMR spectra recorded during the titration of **2** +50 equiv of $LiClO_4$ with TBABr in $CH_3OH/CDCl_3$ (1:9, v/v). The asterisk (*) denotes the residual $CHCl_3$ peak in the NMR solvent.

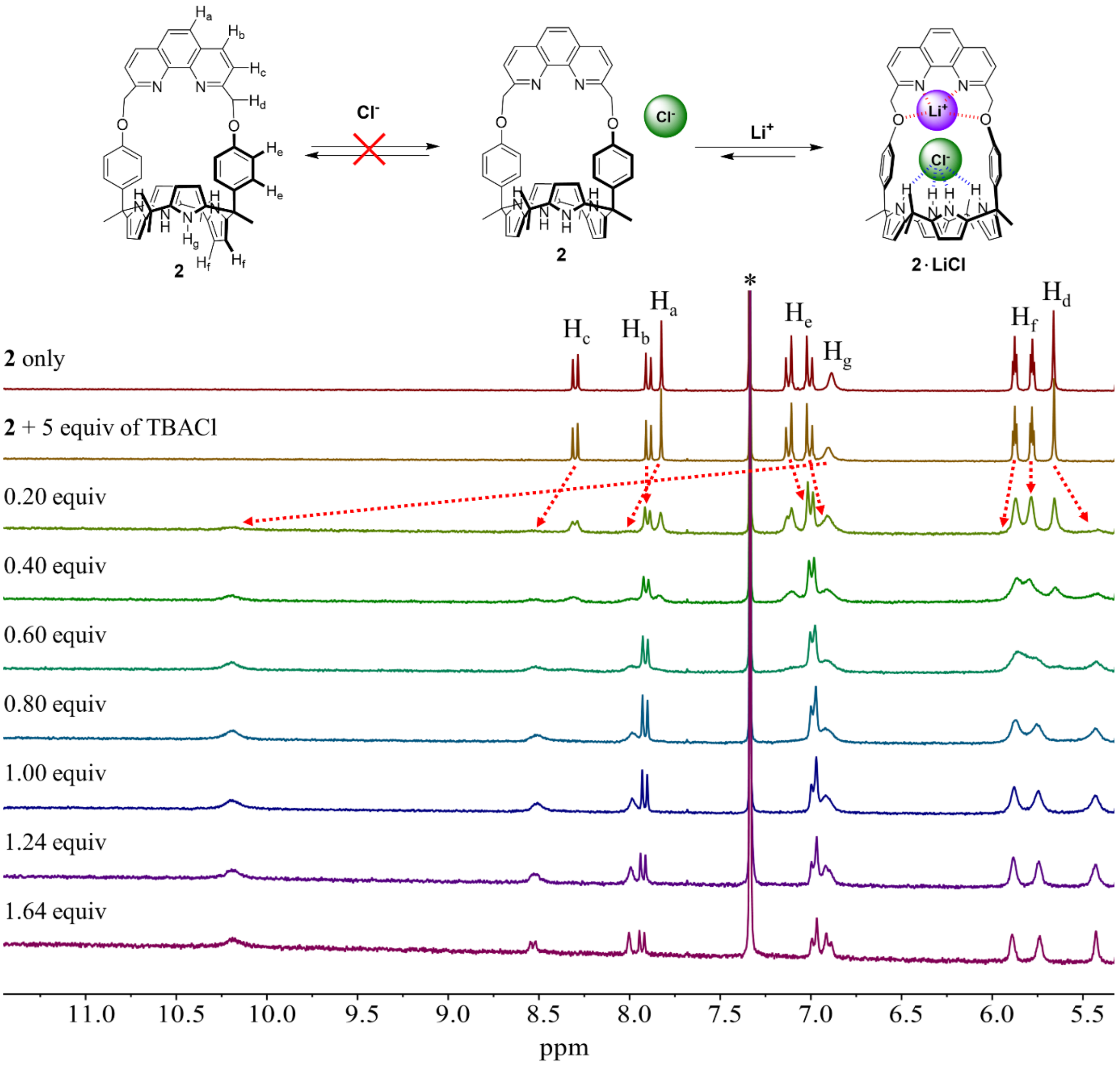

Figure S15. ^{1}H NMR spectra for the titration of **2** with $LiClO_4$ in the presence of 5.0 equiv of TBACl in $CH_3OH/CDCl_3$ (1:9, v/v). The asterisk (*) denotes the residual $CHCl_3$ peak in the NMR solvent.

During the titration of receptor **2** with Li^+ in a solution of $CH_3OH/CDCl_3$ (1/9, v/v) containing 5.0 equiv of Cl^-, two sets of proton signals appeared upon the addition of 0.20 - 0.8 equiv of Cl^-. However, upon the addition of ≥1.00 equiv of Cl^- only one set of proton signals is seen. On the basis of this ^{1}H NMR spectral titration, the association constant of receptor **2** for Li^+ was estimated to be $>10^5$ M^{-1}.

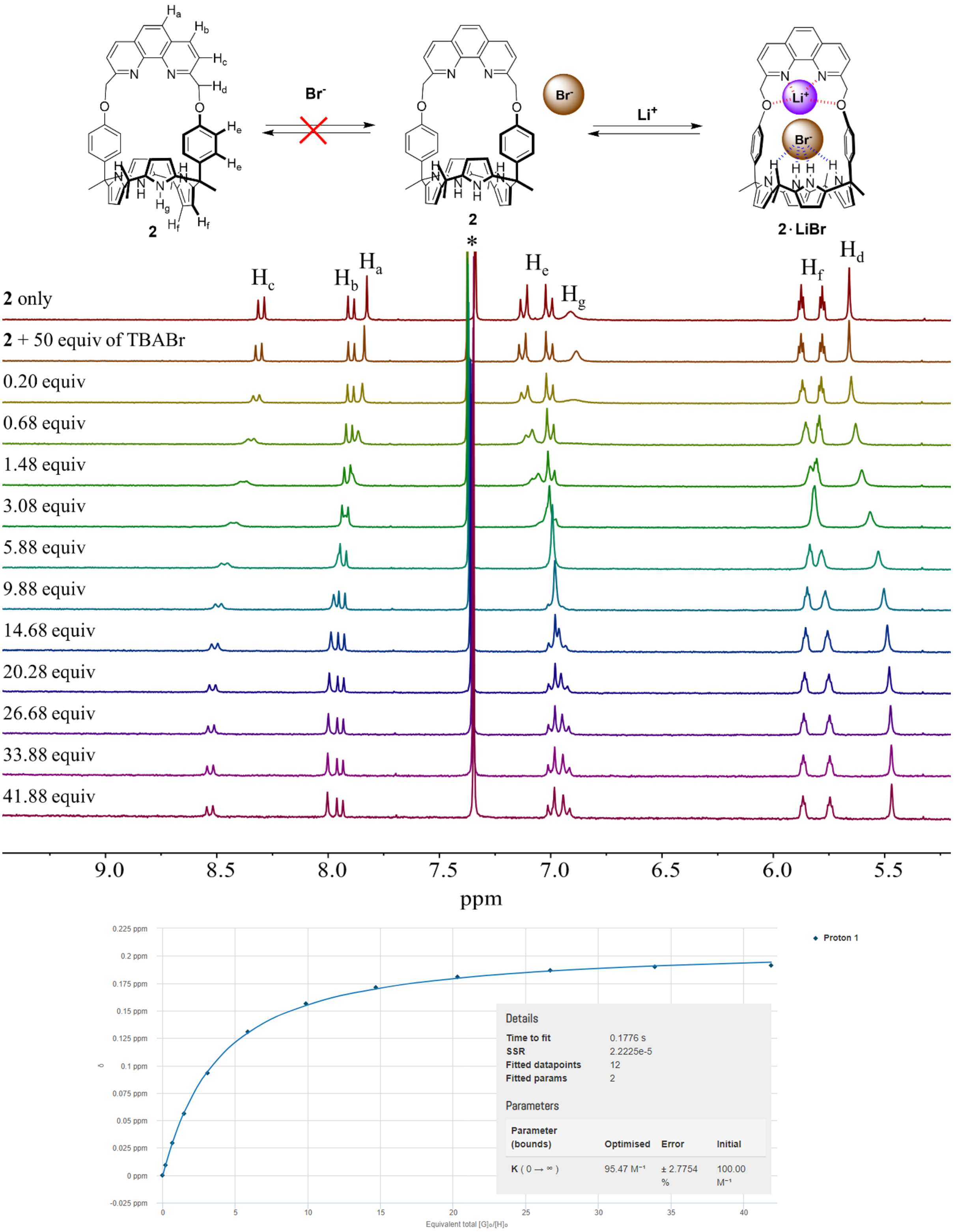

Figure S16. ^{1}H NMR spectra recorded during the titration of **2** with $LiClO_4$ in the presence of 50 equiv of TBABr in $CH_3OH/CDCl_3$ (1:9, v/v). The asterisk (*) denotes the residual $CHCl_3$ peak in the NMR solvent.

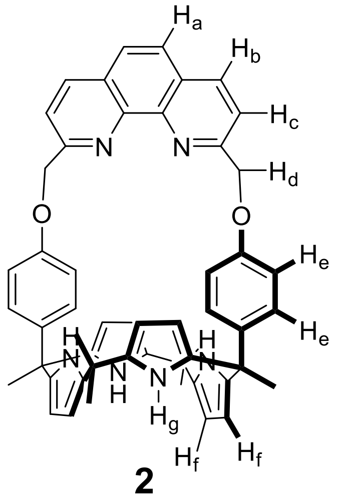

2

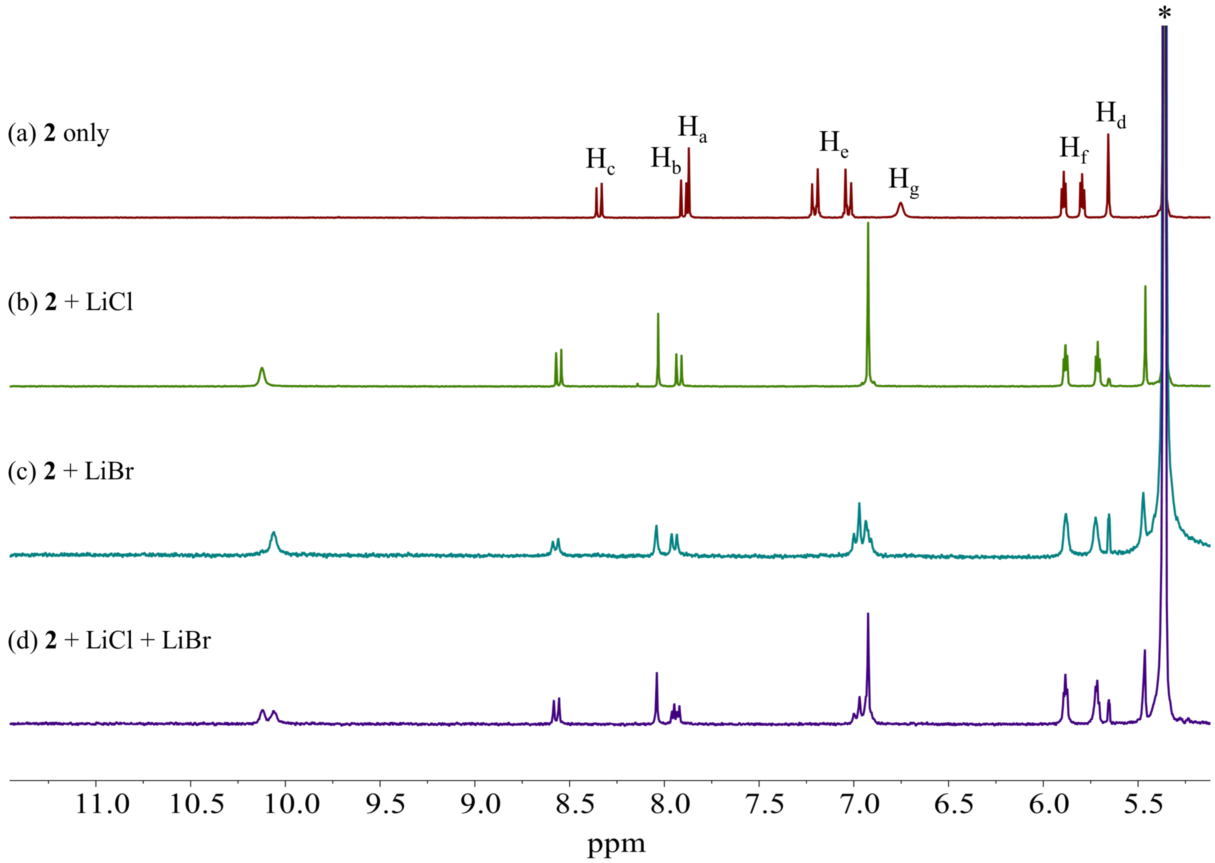

Figure S17. Partial ^{1}H NMR spectra of CD_2Cl_2 solutions of (a) **2** (3 mM) only, (b) **2** + LiCl (100 equiv), (c) **2** + LiBr (100 equiv), and (d) **2** + LiCl (100 equiv) + LiBr (100 equiv). The asterisk (*) denotes the residual CH_2Cl_2 peak in the NMR solvent.

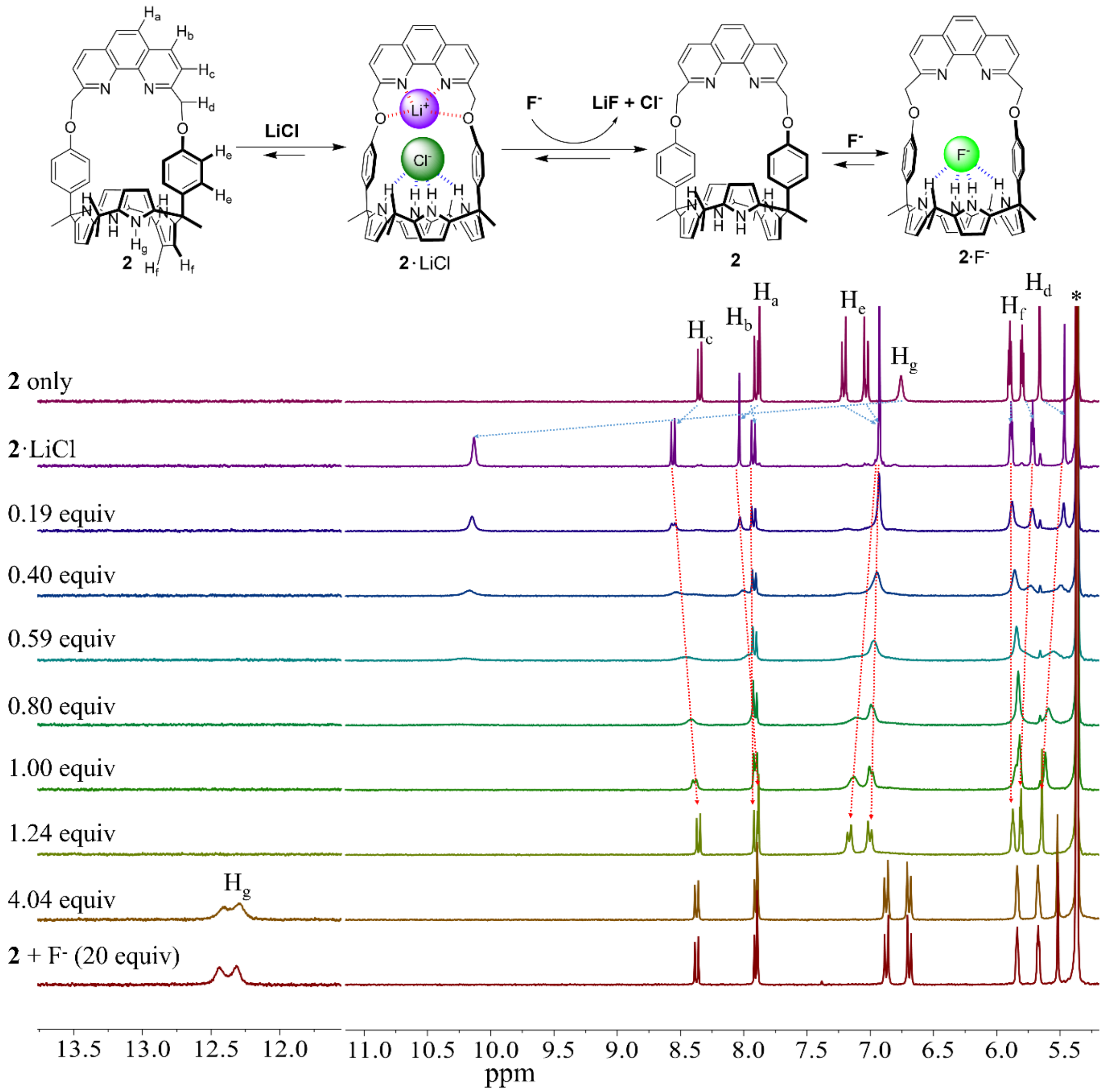

Figure S18. Top: Ion binding behavior of receptor **2** that is proposed to occur when receptor **2** iss titrated with TBAF in the presence of LiCl. Bottom: ^{1}H NMR spectra recorded during the titration of **2**•LiCl (3 mM) with TBAF in CD_2Cl_2 and ^{1}H NMR spectrum of receptor **2** recorded in the presence of 20 equiv of TBAF without LiCl. The asterisk (*) denotes the residual CH_2Cl_2 peak in the NMR solvent.

When TBAF was gradually added to a CD_2Cl_2 solution containing **2**•LiCl, a new set of proton signals completely emerged and, after the addition of 1.2 equiv of F^-, the spectrum evolved to one nearly identical to that of the ion-free receptor **2**. Further addition of F^- led to formation of the fluoride anion complex of **2** featuring a doublet for the NH signal at δ = 12.4 ppm.

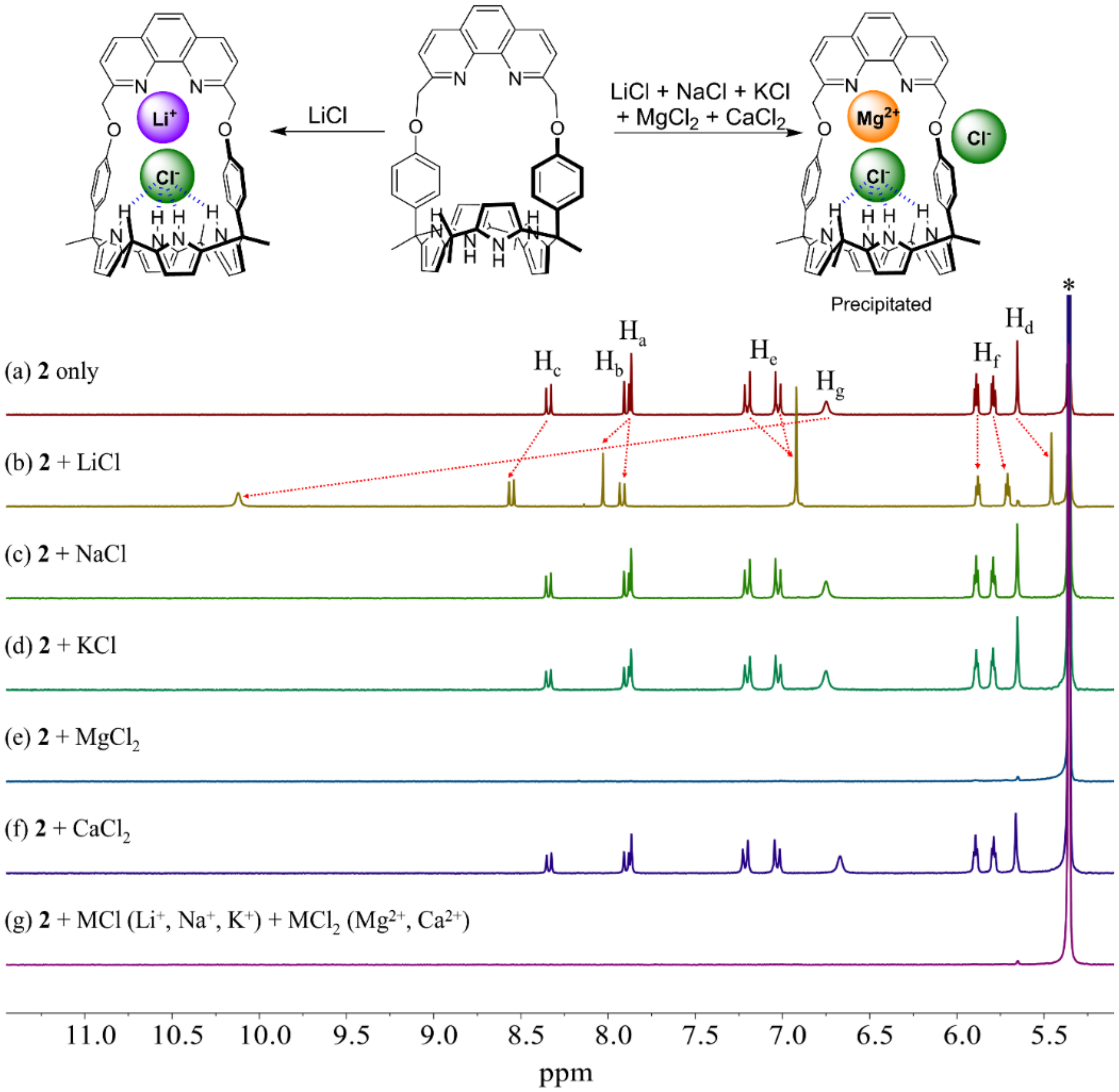

Figure S19. Partial ^{1}H NMR spectra of CD_2Cl_2 solutions of (a) **2** (3 mM) only, (b) **2** + LiCl (100 equiv), (c) **2** + NaCl (100 equiv), (d) **2** + KCl (100 equiv), (e) **2** + $MgCl_2$ (100 equiv), (f) **2** + $CaCl_2$ (100 equiv), and (g) **2** + LiCl + NaCl + KCl + $MgCl_2$ + $CaCl_2$ (100 equiv each). The asterisk (*) denotes the residual CH_2Cl_2 peak in the NMR solvent.

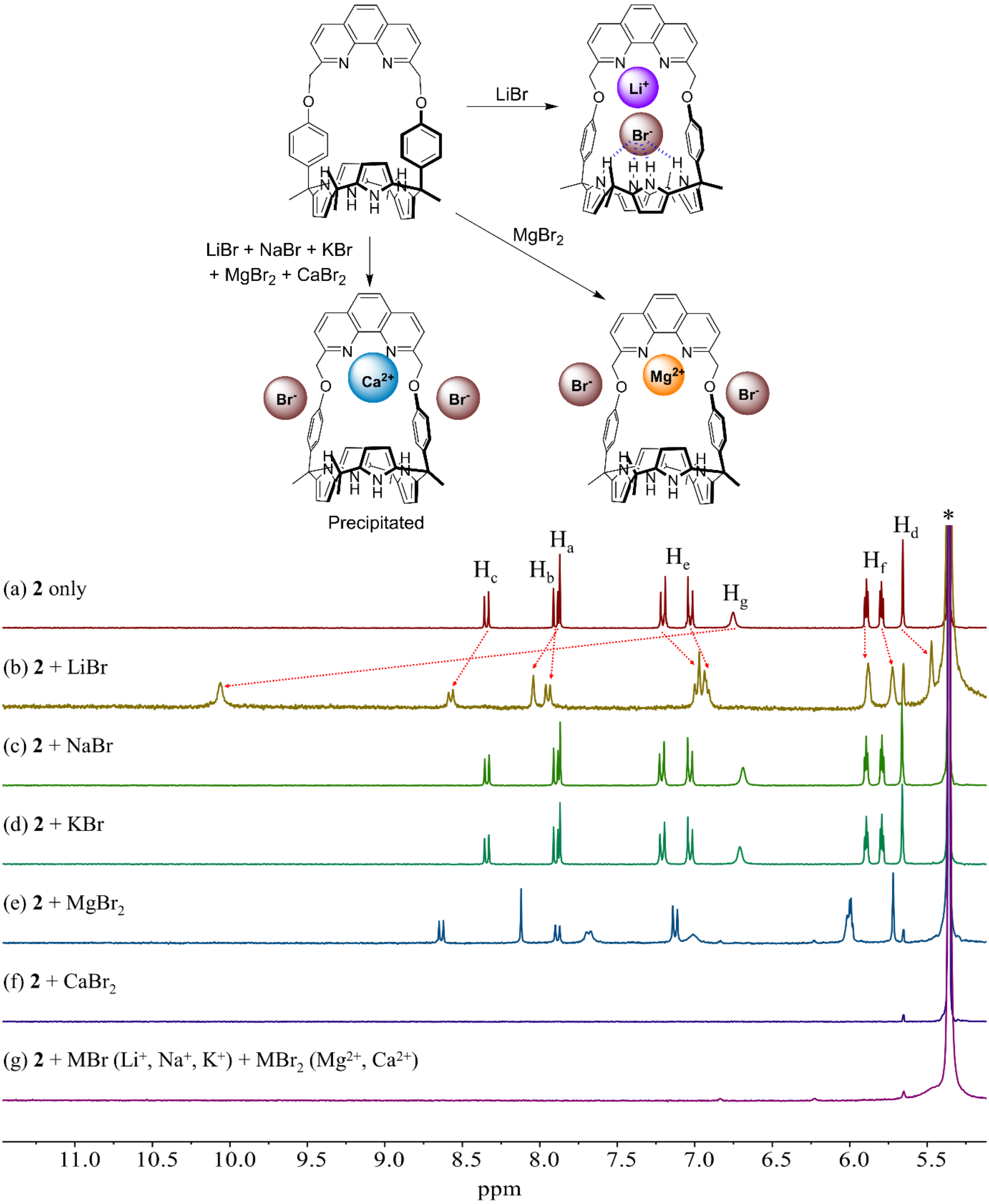

Figure S20. Partial ^{1}H NMR spectra of CD_2Cl_2 solutions of (a) **2** (3 mM) only, (b) **2** + LiBr (100 equiv), (c) **2** + NaBr (100 equiv), (d) **2** + KBr (100 equiv), (e) **2** + $MgBr_2$ (100 equiv), (f) **2** + $CaBr_2$ (100 equiv), and (g) **2** + LiBr + NaBr + KBr + $MgBr_2$ + $CaBr_2$ (100 equiv each). The asterisk (*) denotes the residual CH_2Cl_2 peak in the NMR solvent.

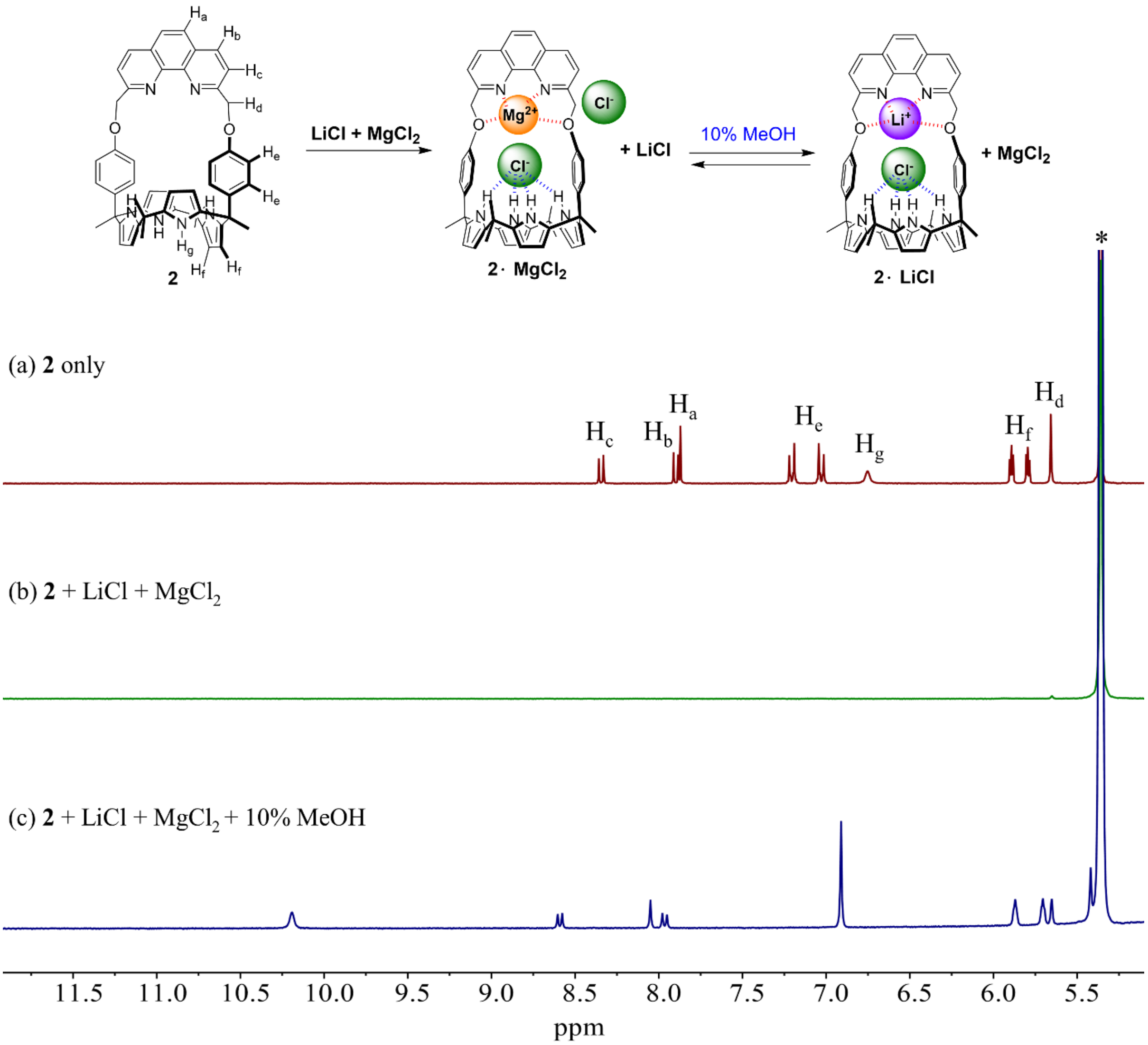

Figure S21. Partial ^{1}H NMR spectra of CD_2Cl_2 solutions of (a) **2** (3 mM) only, (b) **2** + LiCl (100 equiv) + $MgCl_2$ (100 equiv), and (c) after adding 10% MeOH to (b). The asterisk (*) denotes the residual CH_2Cl_2 peak in the NMR solvent.

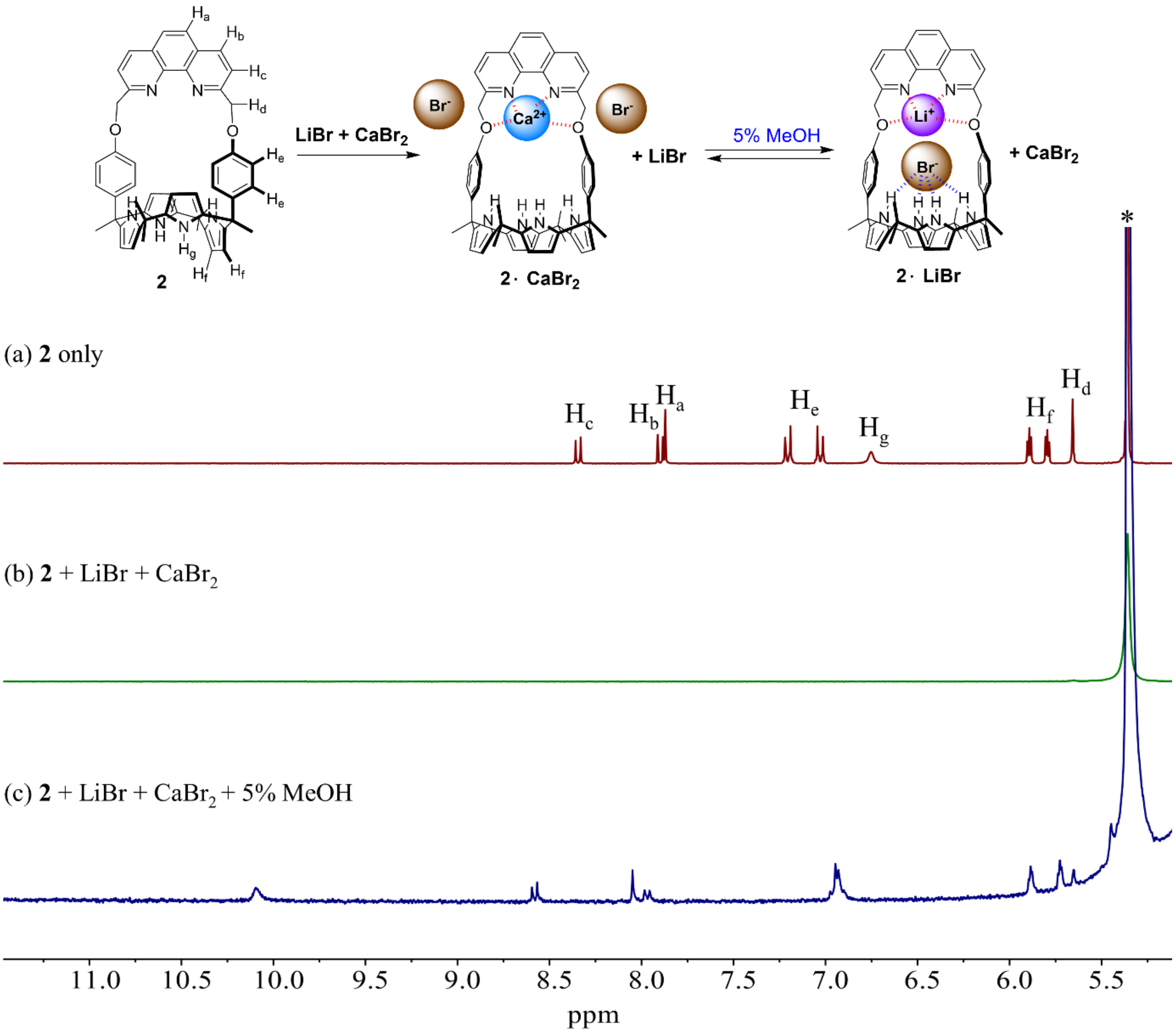

Figure S22. Partial ^{1}H NMR spectra of CD_2Cl_2 solutions of (a) **2** (3 mM) only, (b) **2** + LiBr (100 equiv) + $CaBr_2$ (100 equiv), and (c) after adding 5% MeOH to (b). The asterisk (*) denotes the residual CH_2Cl_2 peak in the NMR solvent.

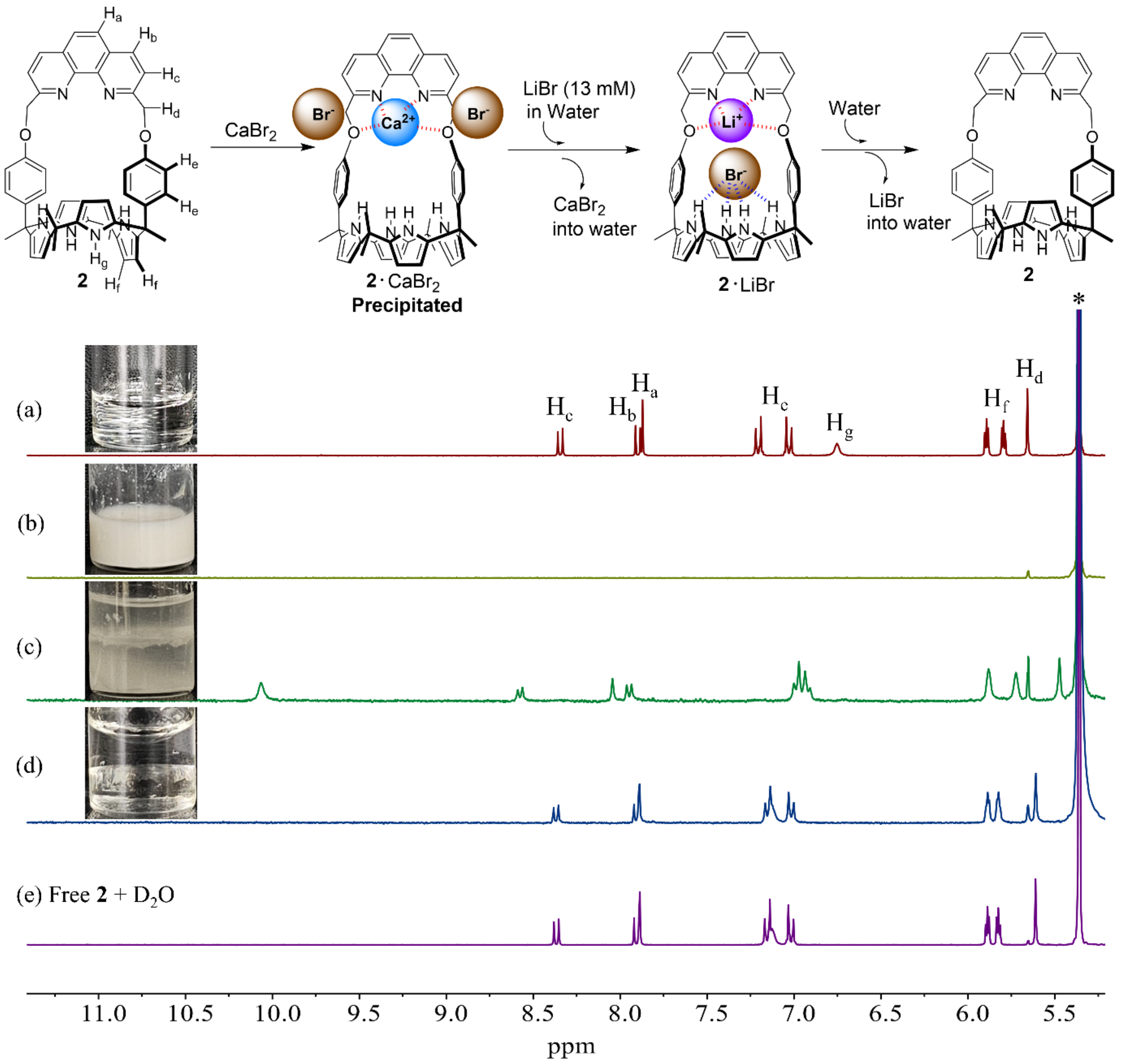

Figure S23. Partial ^{1}H NMR spectra of CD_2Cl_2 solutions of (a) **2** (3 mM) only, (b) **2** + $CaBr_2$ (500 equiv), (c) solution (b) after contacting with a 13 M aqueous D_2O LiBr solution, (d) solution (c) after removing of an aqueous source phase and contacting with an ion-free aqueous D_2O solution, and (e) solution of free **2** after contacting with an ion-free aqueous D_2O solution. The asterisk (*) denotes the residual CH_2Cl_2 peak in the NMR solvent.

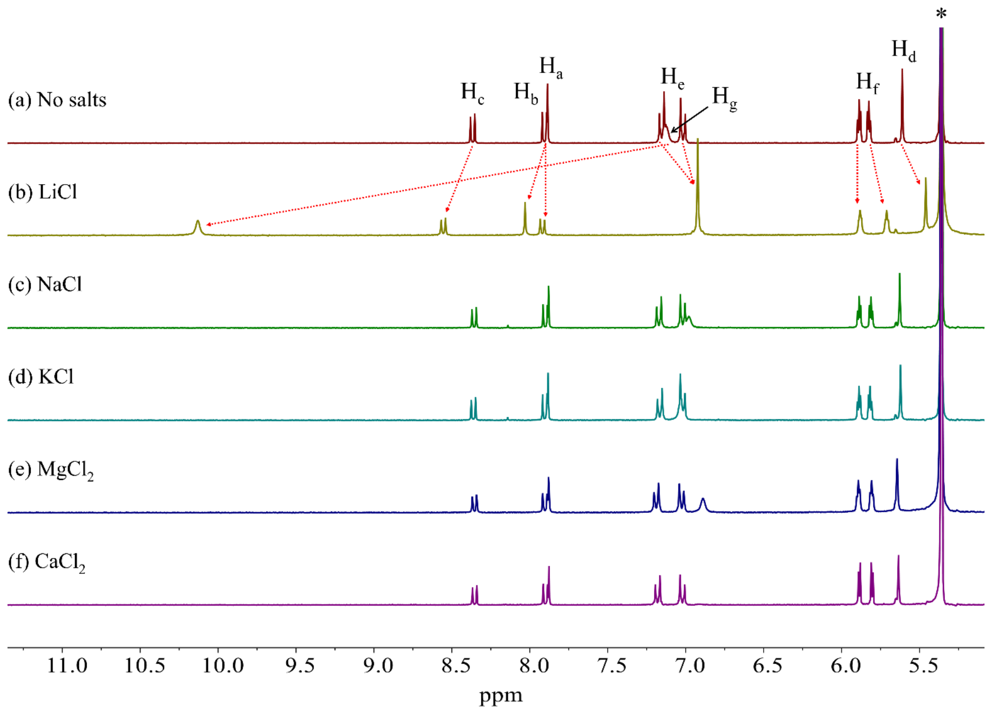

Figure S24. Partial ^{1}H NMR spectra of CD_2Cl_2 organic phases of receptor **2** (3 mM) after contacting with (a) an ion-free aqueous D_2O solution, (b) an aqueous D_2O solution containing LiCl (excess), (c) an aqueous D_2O solution containing NaCl (excess) (d) an aqueous D_2O solution containing KCl (excess), (e) an aqueous D_2O solution containing $MgCl_2$ (excess), and (f) an aqueous D_2O solution containing $CaCl_2$ (excess). The asterisk (*) denotes the residual CH_2Cl_2 peak in the NMR solvent.

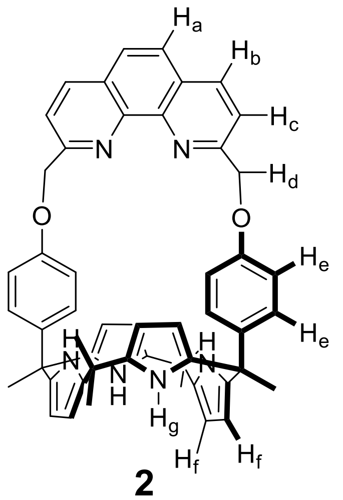

2

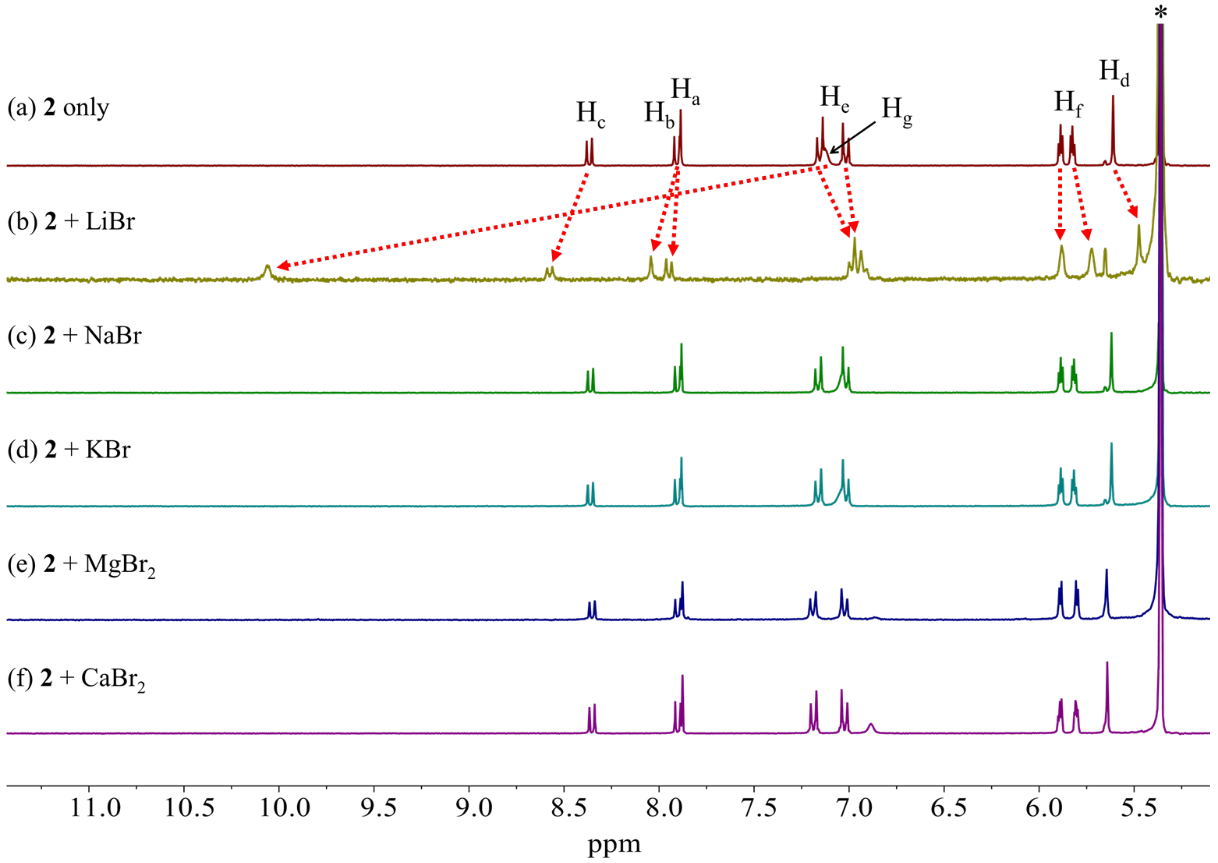

Figure S25. Partial ^{1}H NMR spectra of the CD_2Cl_2 organic phases of **2** (3 mM) after contacting with (a) an ion-free aqueous D_2O solution, (b) an aqueous D_2O solution containing LiBr (excess), (c) an aqueous D_2O solution containing NaBr (excess) (d) an aqueous D_2O solution containing KBr (excess), (e) an aqueous D_2O solution containing $MgBr_2$ (excess), and (f) an aqueous D_2O solution containing $CaBr_2$ (excess). The asterisk (*) denotes the residual CH_2Cl_2 peak in the NMR solvent.

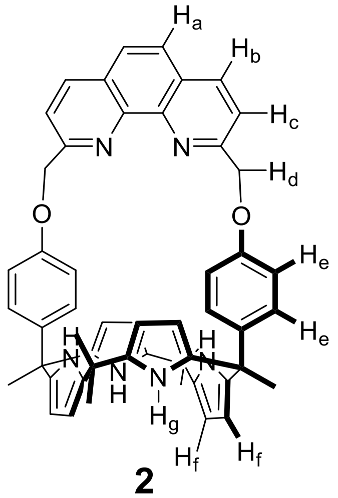

2

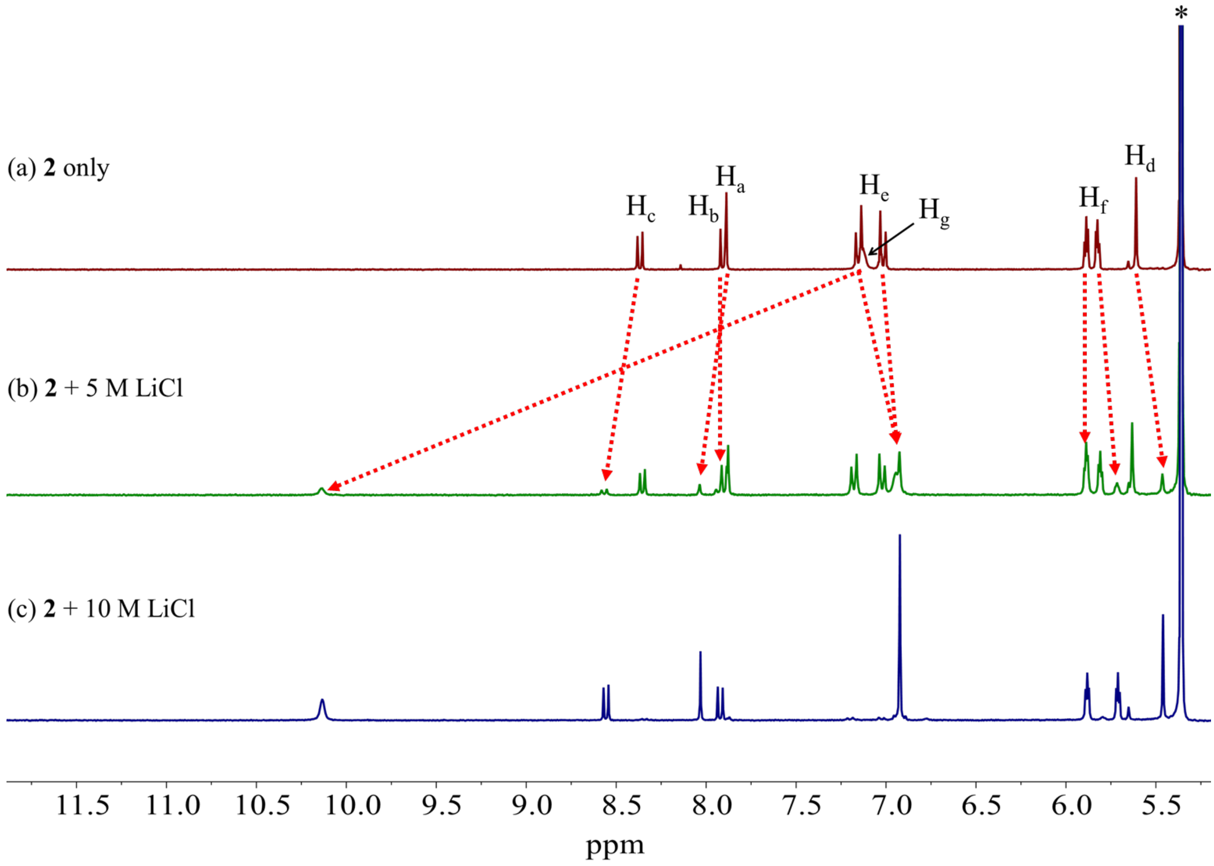

Figure S26. Partial ^{1}H NMR spectra of CD_2Cl_2 solutions of **2** (3 mM) after contacting with (a) an ion-free aqueous D_2O solution, (b) a 5 M LiCl aqueous D_2O solution, and (c) a 10 M LiCl aqueous D_2O solution. Percentages of the receptor bound to LiCl are estimated to be 24% and ≈ 100%, respectively. The asterisk (*) denotes the residual CH_2Cl_2 peak in the NMR solvent.

Single Mass Analysis
Tolerance = 30.0 PPM / DBE: min = -10.0, max = 40.0
Element prediction: Off
Monoisotopic Mass, Odd and Even Electron Ions
131 formula(e) evaluated with 5 results within limits (up to 5 closest results for each mass)
Elements Used:
C: 1-100 H: 1-1000 Li: 1-1 N: 1-6 O: 1-2

Mass	Calc. Mass	mDa	PPM	DBE	Formula	C	H	Li	N	O
795.4105	795.4250	-14.5	-18.2	30.5	C54 H52 Li N4 O2	54	52	1	4	2
	795.4125	-2.0	-2.5	31.0	C53 H50 Li N5 O2	53	50	1	5	2
	795.3999	10.6	13.3	31.5	C52 H48 Li N6 O2	52	48	1	6	2
	795.4052	5.3	6.7	35.0	C59 H50 Li N O	59	50	1	1	1
	795.3927	17.8	22.4	35.5	C58 H48 Li N2 O	58	48	1	2	1

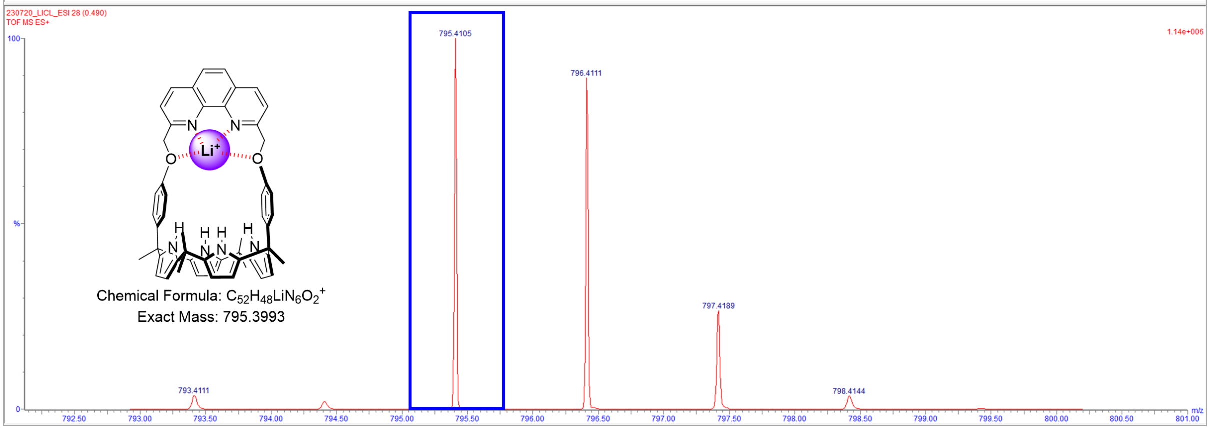

Figure S27. HRMS spectrum of the organic phase of receptor **2** after contacting with an aqueous D_2O solution containing 10 M of LiCl.

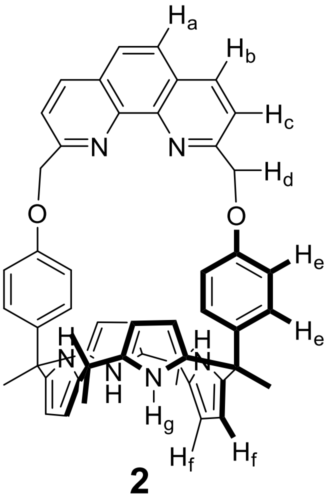

2

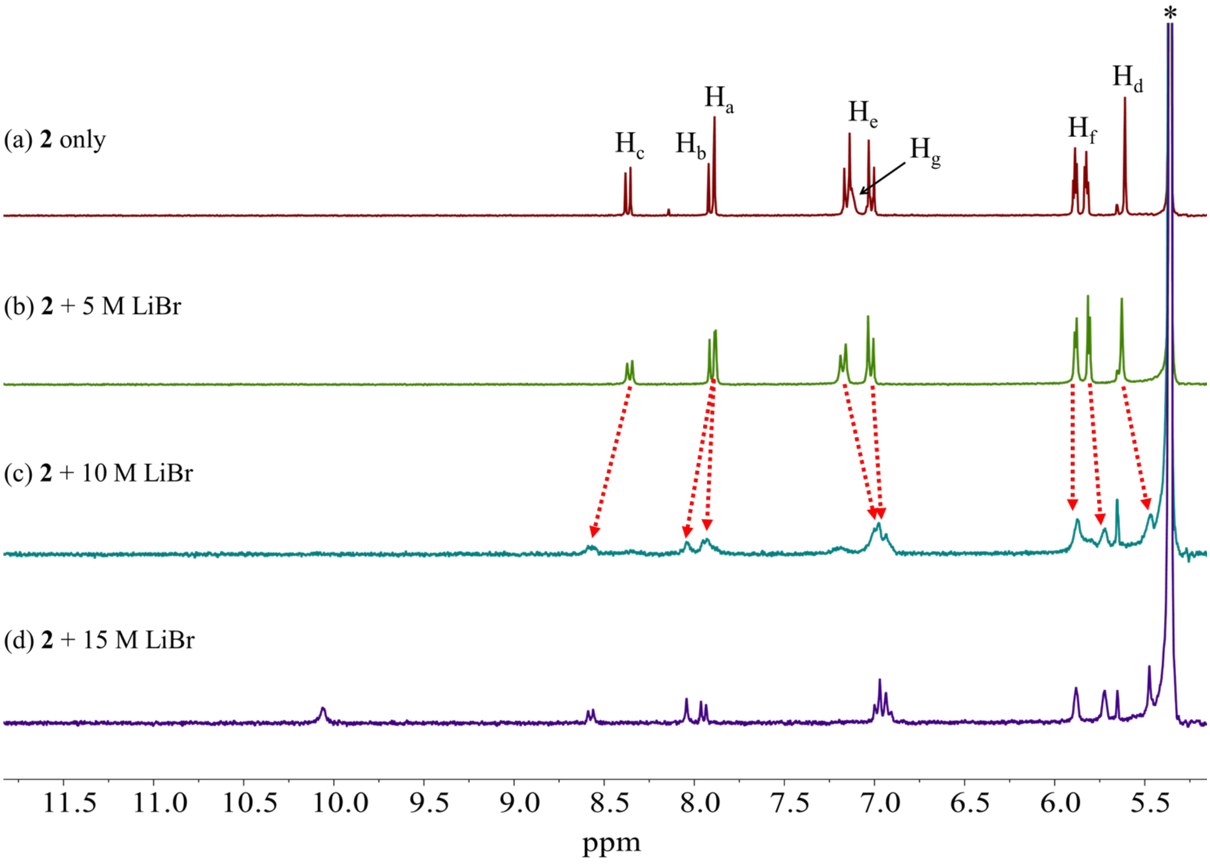

Figure S28. Partial ^{1}H NMR spectra of CD_2Cl_2 solutions of **2** (3 mM) after contacting with (a) ion-free D_2O, (b) a 5 M LiBr aqueous D_2O solution, (c) a 10 M LiBr aqueous D_2O solution, and (d) a 15 m LiBr aqueous D_2O solution. The asterisk (*) denotes the residual CH_2Cl_2 peak in the NMR solvent.

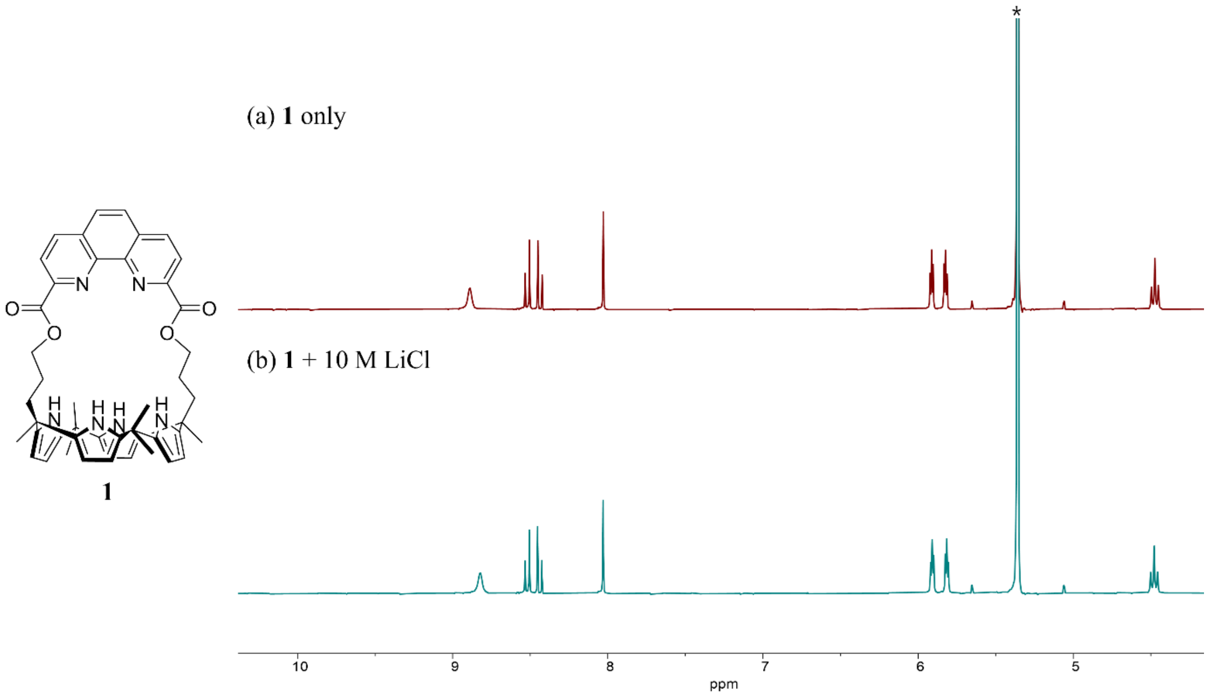

Figure S29. Partial ^{1}H NMR spectra of CD_2Cl_2 solutions of **1** (3 mM) after contacting with (a) ion-free D_2O, and (b) a 10 M LiCl aqueous D_2O solution. The asterisk (*) denotes the residual CH_2Cl_2 peak in the NMR solvent.

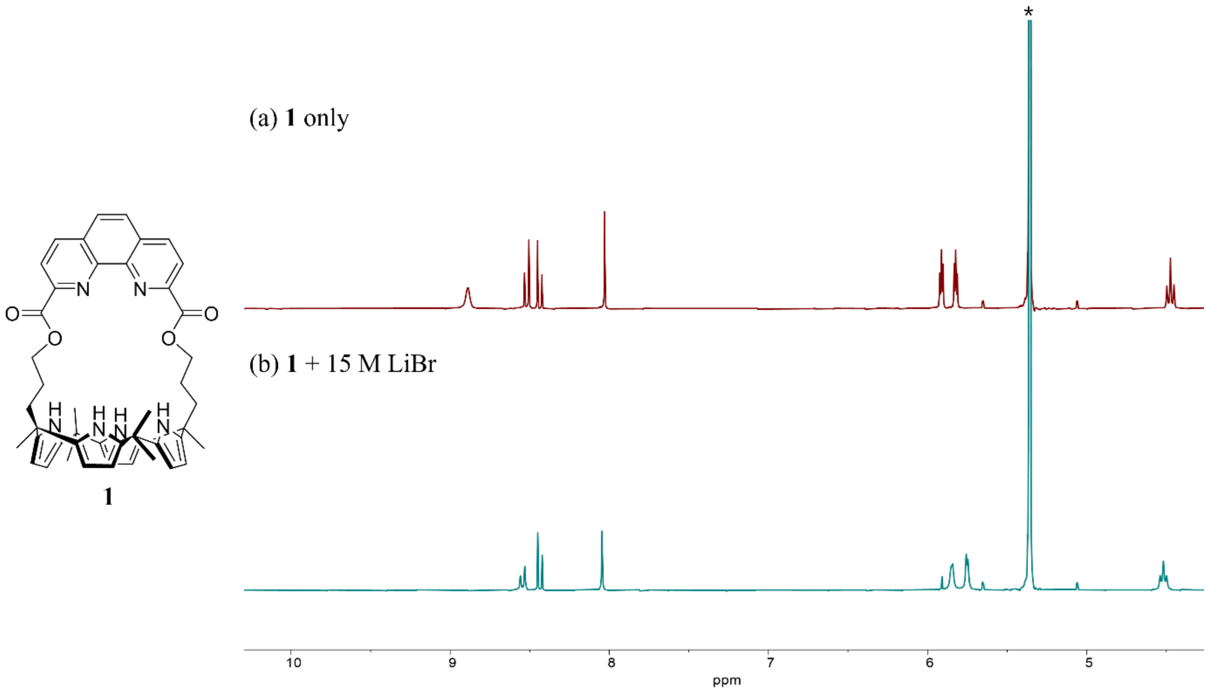

Figure S30. Partial ^{1}H NMR spectra of CD_2Cl_2 solutions of **1** (3 mM) after contacting with (a) ion-free D_2O, and (b) a 15 M LiBr aqueous D_2O solution. The asterisk (*) denotes the residual CH_2Cl_2 peak in the NMR solvent.

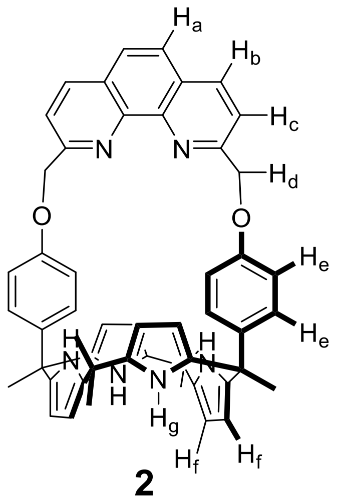

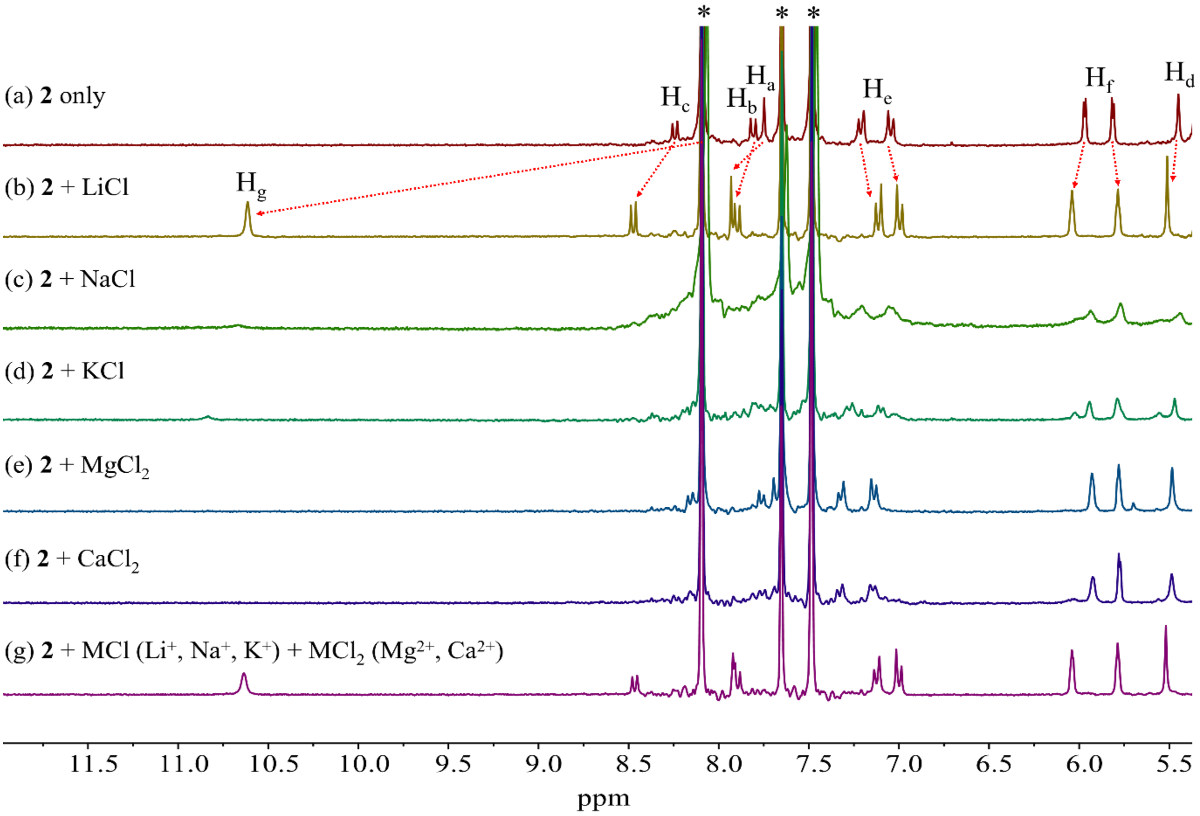

Figure S31. Partial ^{1}H NMR spectra of nitrobenzene-*d*$_5$ solutions of **2** (3 mM) after contacting with (a) ion-free D_2O, (b) an aqueous D_2O solution containing LiCl (excess), (c) an aqueous D_2O solution containing NaCl (excess) (d) an aqueous D_2O solution containing KCl (excess), (e) an aqueous D_2O solution containing $MgCl_2$ (excess), (f) an aqueous D_2O solution containing $CaCl_2$ (excess), and an aqueous D_2O solution containing all the chloride salts (5 M each). The asterisks (*) denote the residual nitrobenzene peaks in the NMR solvent.

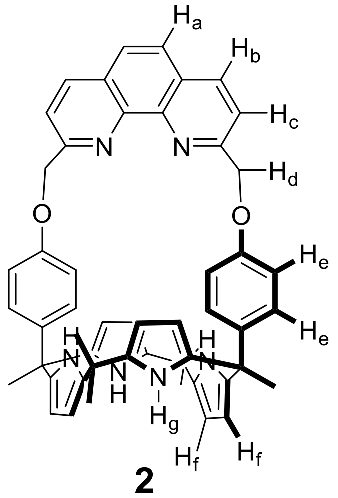

2

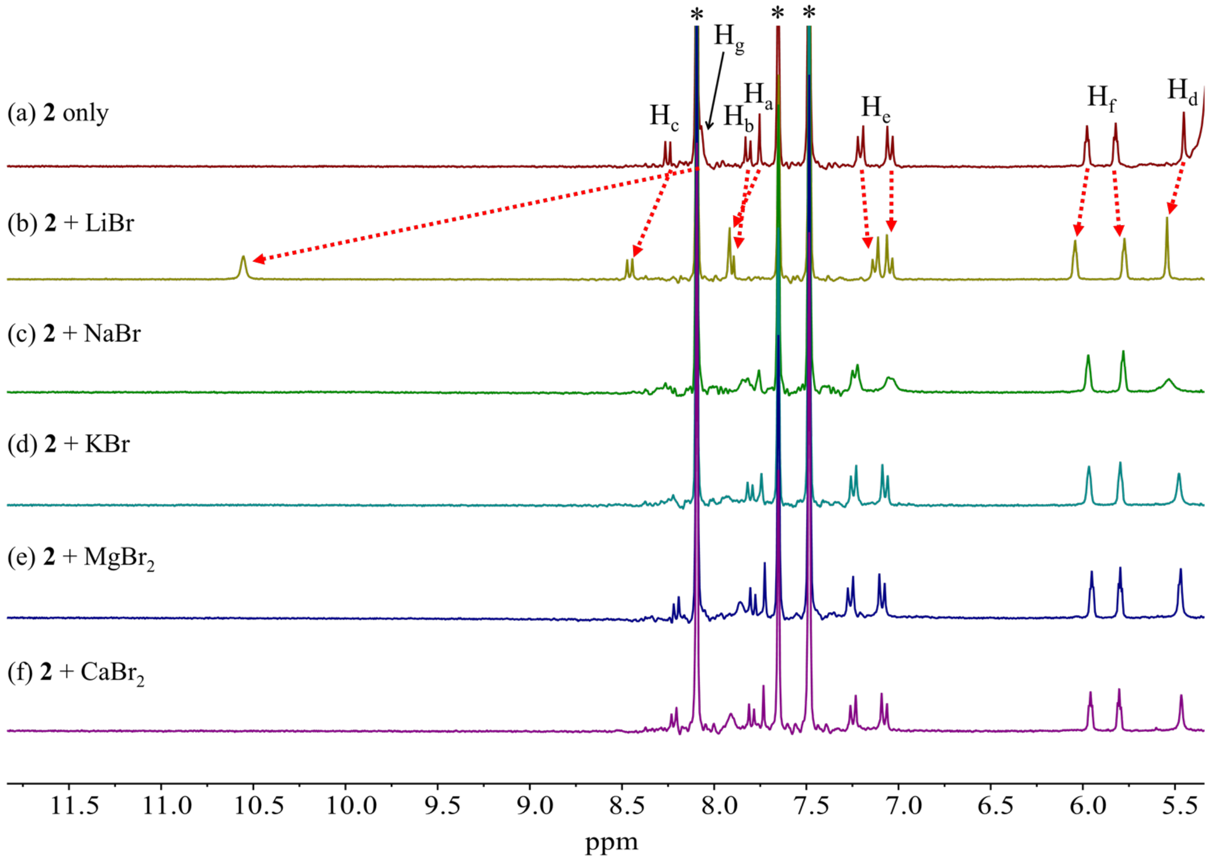

Figure S32. Partial ^{1}H NMR spectra of nitrobenzene-d_5 solutions of **2** (3 mM) after contacting with (a) ion-free H_2O, (b) an aqueous H_2O solution containing LiBr (excess), (c) an aqueous H_2O solution containing NaBr (excess) (d) an aqueous H_2O solution containing KBr (excess), (e) an aqueous H_2O solution containing $MgBr_2$ (excess), and (f) an aqueous H_2O solution containing $CaBr_2$ (excess). The asterisks (*) denote the residual nitrobenzene peaks in the NMR solvent.

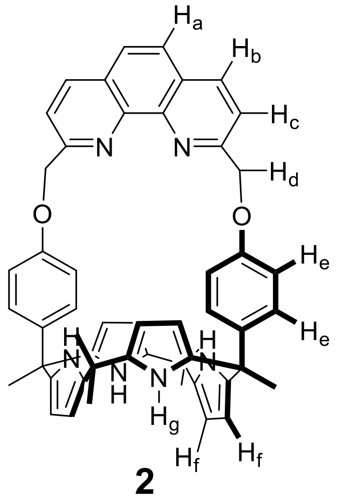

2

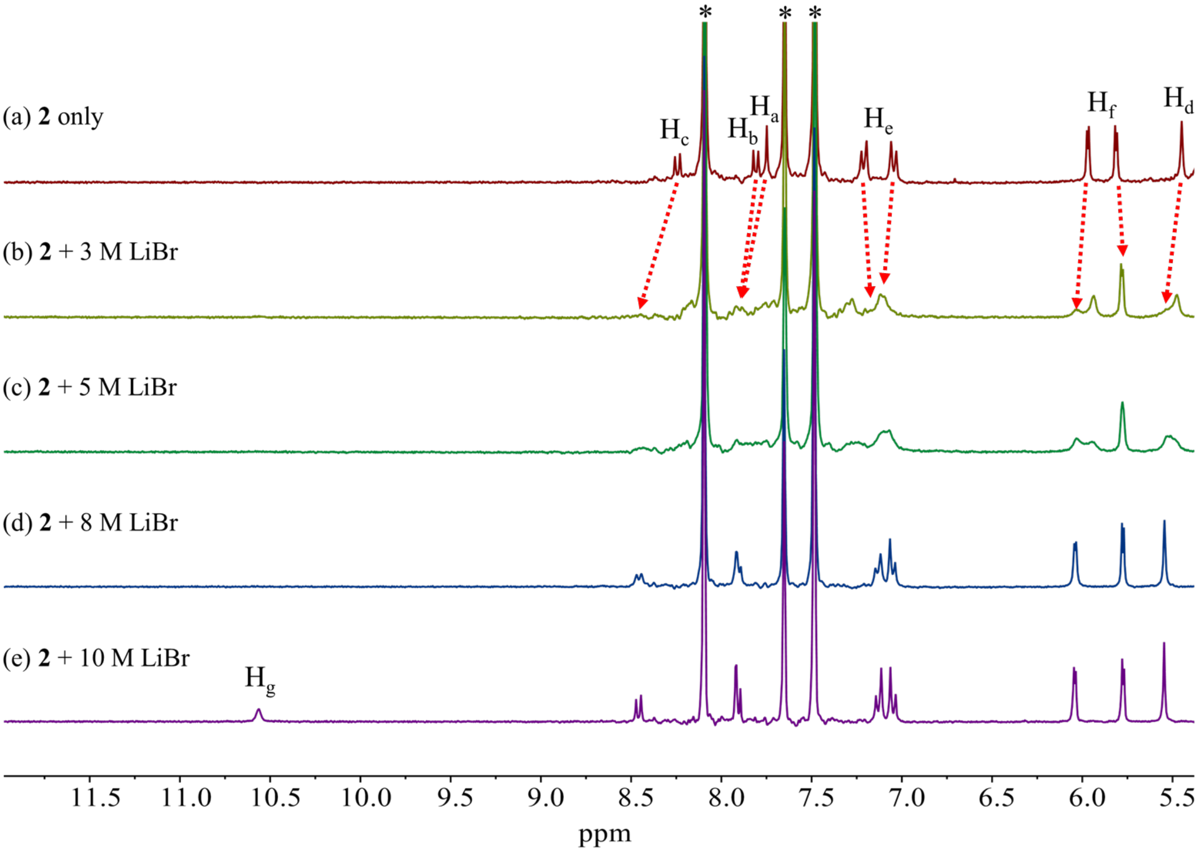

Figure S33. Partial ^{1}H NMR spectra of nitrobenzene-d_5 layers of **2** (3 mM) after contacting with (a) ion-free D_2O, (b) an aqueous D_2O solution containing 3 M LiBr, (c) an aqueous D_2O solution containing 5 M LiBr, (d) an aqueous D_2O solution containing 8 M LiBr, and (e) an aqueous D_2O solution containing 10 M LiBr. The asterisks denote the residual nitrobenzene peaks in the NMR solvent.

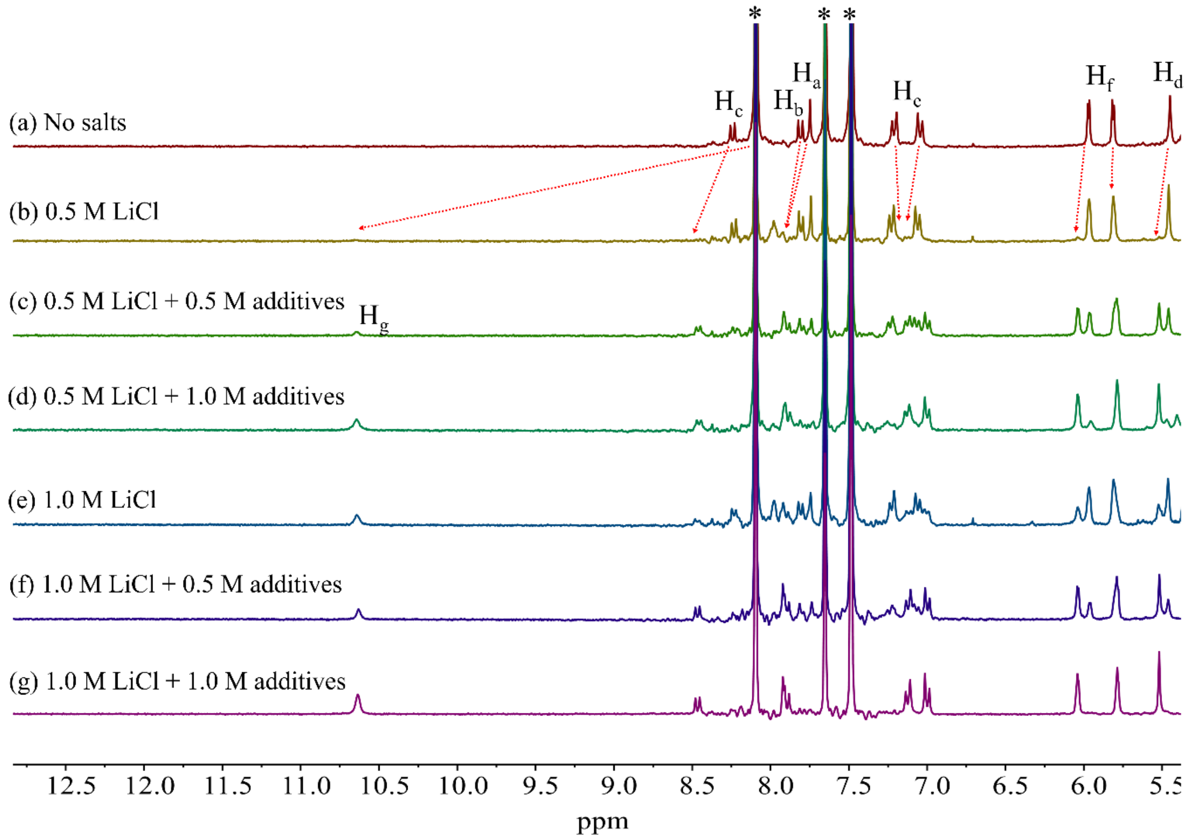

Figure S34. Partial ^{1}H NMR spectra of nitrobenzene-d_5 phases of **2** (3 mM) after contacting with (a) ion-free D_2O, (b) an aqueous D_2O solution containing 0.5 M LiCl, (c) an aqueous D_2O solution containing 0.5 M LiCl + 0.5 M NaCl + 0.5 M KCl + 0.5 M $MgCl_2$ + 0.5 M $CaCl_2$, (d) an aqueous D_2O solution containing 0.5 M LiCl + 1.0 M NaCl + 1.0 M KCl + 1.0 M $MgCl_2$ + 1.0 M $CaCl_2$, (e) an aqueous D_2O solution containing 1.0 M LiCl, (f) an aqueous D_2O solution containing 1.0 M LiCl + 0.5 M NaCl + 0.5 M KCl + 0.5 M $MgCl_2$ + 0.5 M $CaCl_2$, and (g) an aqueous D_2O solution containing 1.0 M LiCl + 1.0 M NaCl + 1.0 M KCl + 1.0 M $MgCl_2$ + 1.0 M $CaCl_2$.

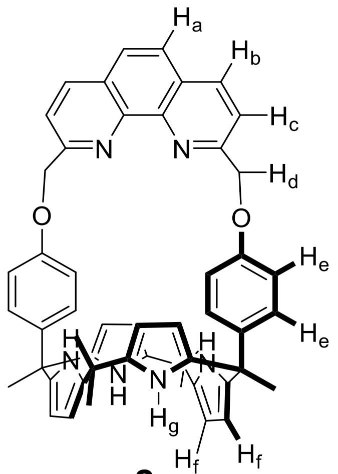

2

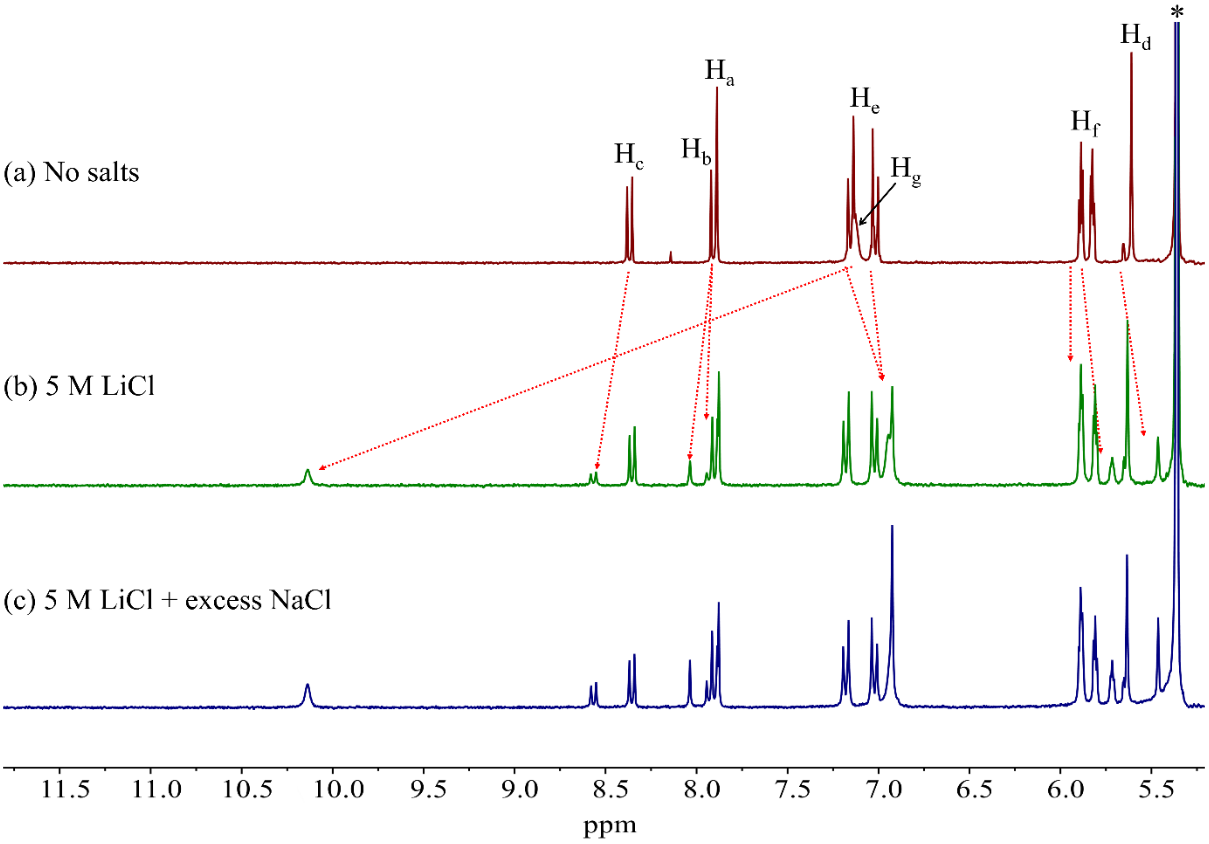

Figure S35. Partial ^{1}H NMR spectra of CD_2Cl_2 solutions of **2** (3 mM) after contacting with (a) ion-free D_2O, (b) an aqueous D_2O solution containing 5 M LiCl, and (c) an aqueous D_2O solution containing 5 M LiCl + Sat. NaCl. Percentages of the receptor extracting LiCl are estimated to be 24% and 36% respectively. The asterisk (*) denotes the residual CH_2Cl_2 peak in the NMR solvent.

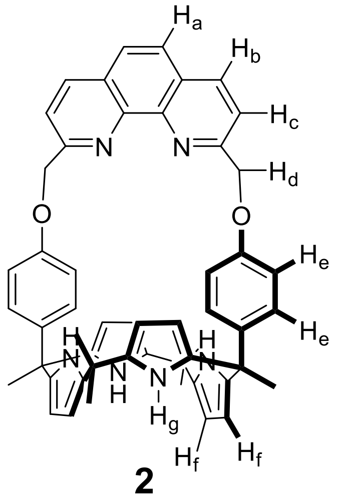

2

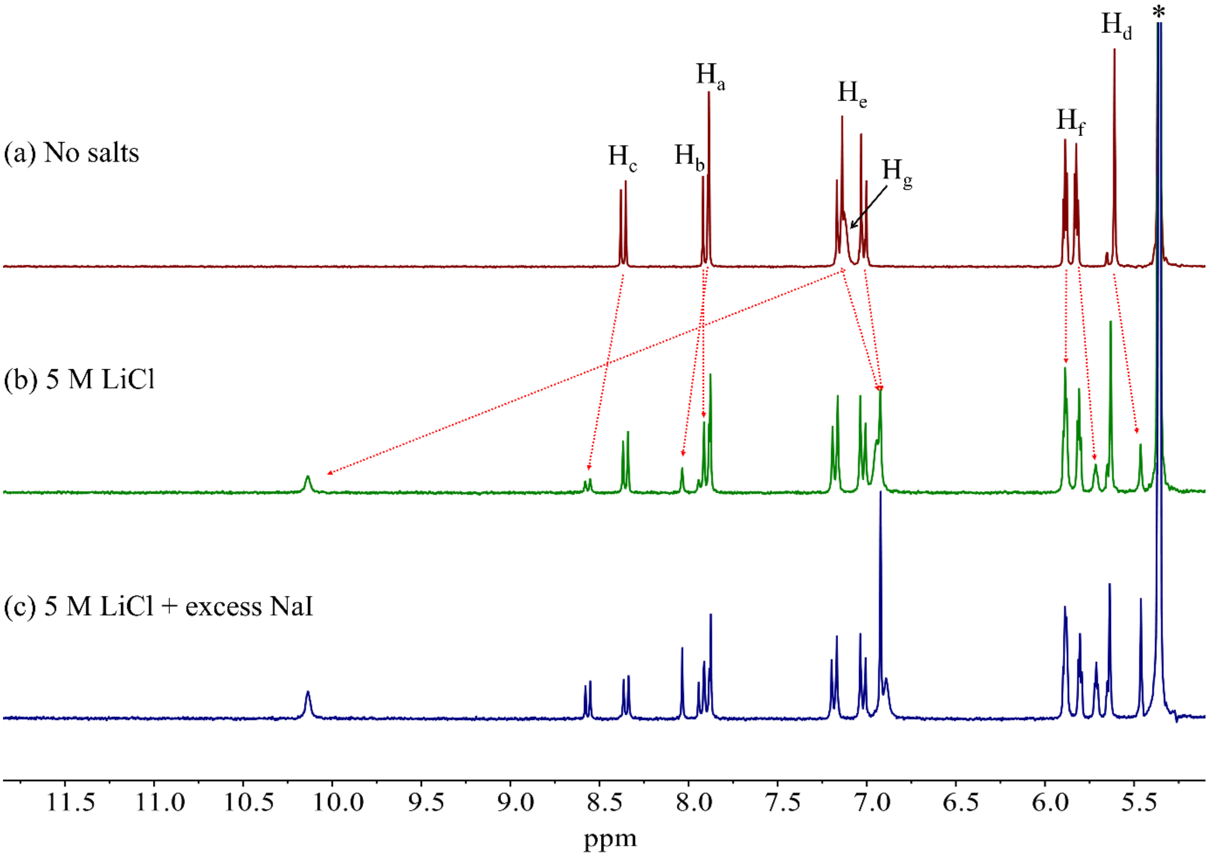

Figure S36. Partial ^{1}H NMR spectra of CD_2Cl_2 solutions of **2** (3 mM) after contacting with (a) ion-free D_2O, (b) an aqueous D_2O solution containing 5 M LiCl, and (c) an aqueous D_2O solution containing 5 M LiCl + Sat. NaI. Percentages of the receptor extracting LiCl are estimated to be 24% and 42% respectively. The asterisk (*) denotes the residual CH_2Cl_2 peak in the NMR solvent.

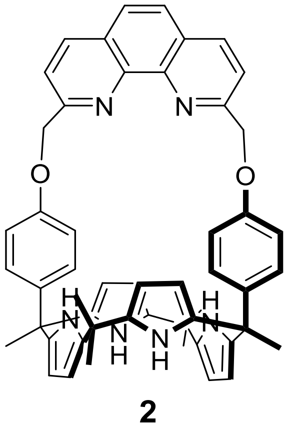

2

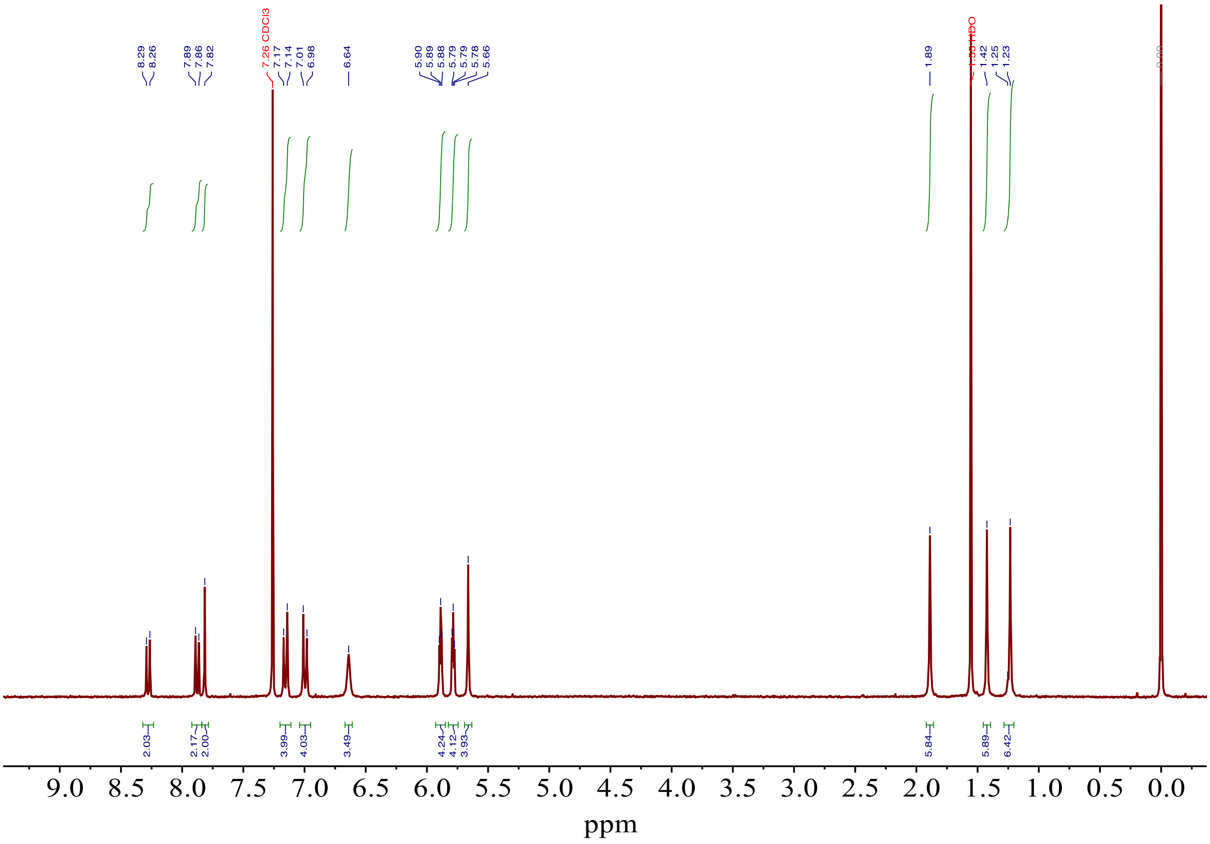

Figure S37. ^{1}H NMR spectrum of **2** recorded in $CDCl_3$.

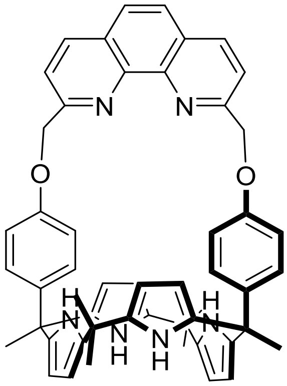

2

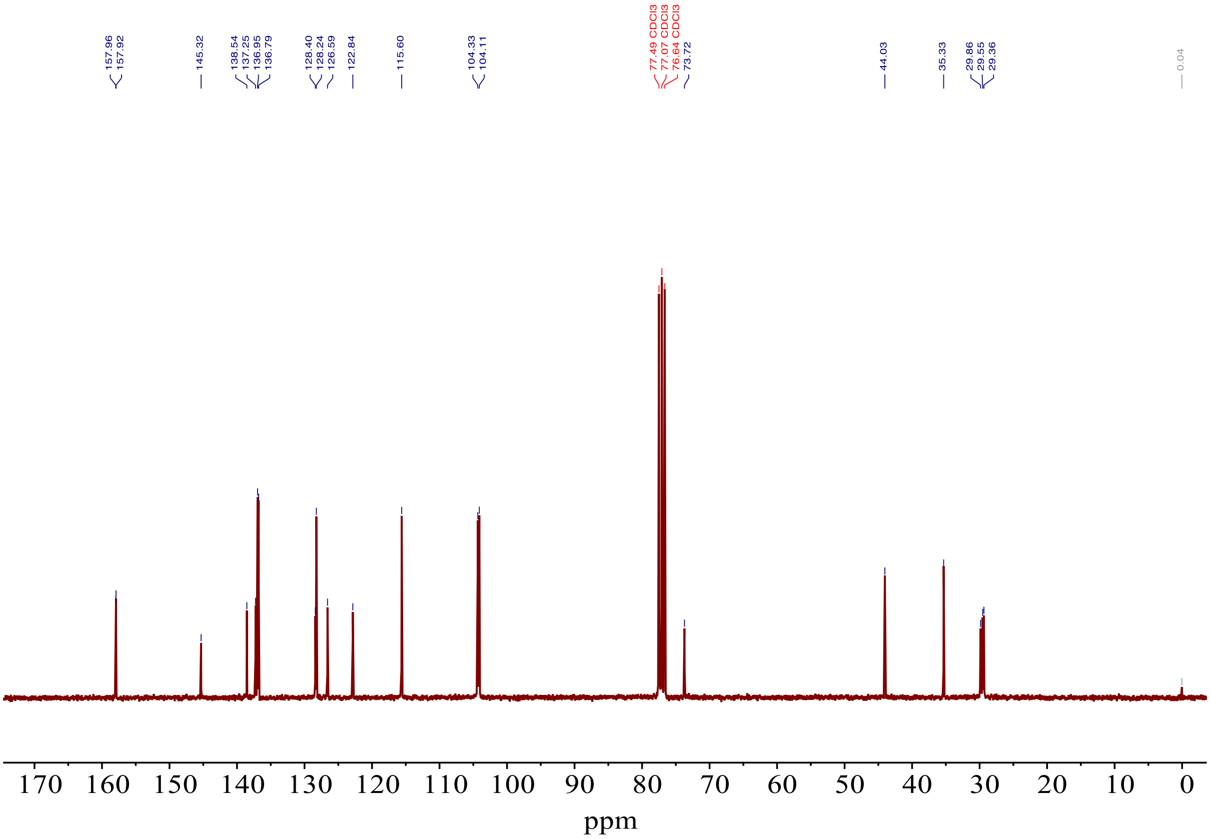

Figure S36. ^{13}C NMR spectrum of **2** recorded in $CDCl_3$.

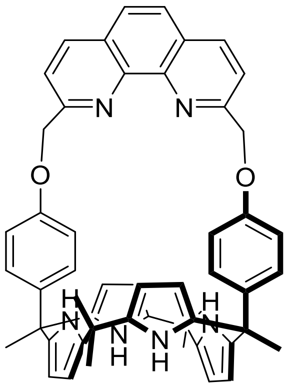

2

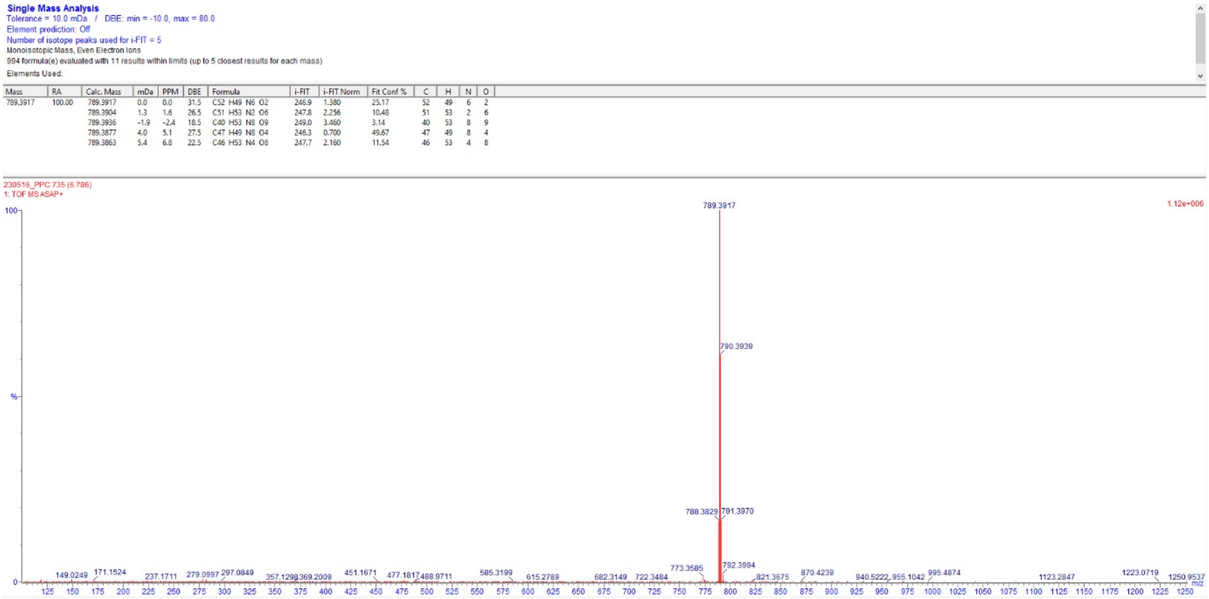

Mass	RA	Calc. Mass	mDa	PPM	DBE	Formula	i-FIT	i-FIT Norm	Fit Conf %	C	H	N	O
789.3917	100.00	789.3917	0.0	0.0	31.5	C52 H49 N6 O2	246.9	1.380	25.17	52	49	6	2
		789.3904	1.3	1.6	26.5	C51 H53 N2 O6	247.8	2.256	10.48	51	53	2	6
		789.3936	-1.9	-2.4	18.5	C40 H53 N8 O9	249.0	3.460	3.14	40	53	8	9
		789.3877	4.0	5.1	27.5	C47 H49 N8 O4	246.3	0.700	49.67	47	49	8	4
		789.3863	5.4	6.8	22.5	C46 H53 N4 O8	247.7	2.160	11.54	46	53	4	8

Figure S39. High resolution QTOF mass spectrum of receptor **2**.

Table S1. Crystal data and structure refinement for **2**•LiBr.

Empirical formula	C56 H58 Br Cl6 Li N6 O4	
Formula weight	1178.63	
Temperature	123.15	
Wavelength	0.71073	
Crystal system	orthorhombic	
Space group	P m n 21	
Unit cell dimensions	a = 13.9123(13)Å	α = 90°.
	b = 14.8656(13)Å	β = 90°.
	c = 13.0840(10)Å	γ = 90°.
Volume	2706.0(4)$Å^3$	
Z	2	
Density (calculated)	1.447Mg/m^3	
Absorption coefficient	1.109mm^{-1}	
F(000)	1216	
Crystal size	0.318 x 0.291 x 0.041 mm^3	
Theta range for data collection	2.538 to 21.494°.	
Index ranges	-18<=h<=7, -18<=k<=18, -16<=l<=16	
Reflections collected	5505	
Independent reflections	4305 [R(int) = 0.0536]	
Completeness to theta = 25.25°	99.8 %	
Absorption correction	multi-scan	
Refinement method	Full-matrix least-squares on F^2	
Data / restraints / parameters	5505 / 10 / 385	
Goodness-of-fit on F^2	1.027	
Final R indices [I>2sigma(I)]	R1 = 0.0681, wR2 = 0.1710	
R indices (all data)	R1 = 0.0909, wR2 = 0.1889	
Extinction coefficient	-	
Largest diff. peak and hole	1.523 and -0.738 e.$Å^{-3}$	
CCDC no.	2356046	

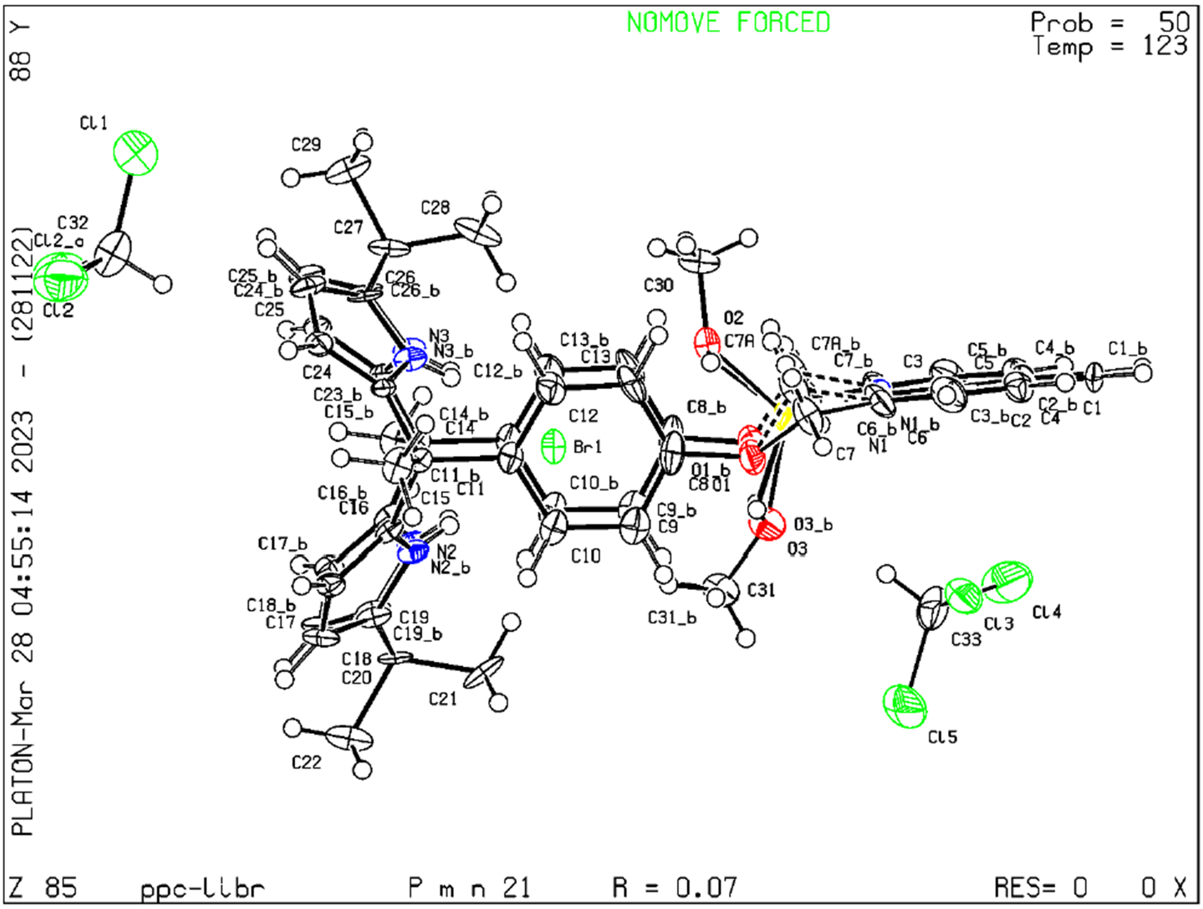

Figure S40. View of the lithium bromide complex of **2** showing a partial atom labeling scheme. Displacement ellipsoids are scaled to the 50% probability level. Most hydrogen atoms have been removed for clarity. Dashed lines are indicative of H-bonding interactions.

Geometrical coordinates of the optimized structures

Ion-free 2

Cartesian coordinates:

Symbol	X	Y	Z
N	8.899	4.948	8.547
H	9.208	4.022	8.765
O	9.989	10.988	5.162
N	8.973	5.315	4.268
H	8.93	6.271	3.999
C	9.414	5.774	7.603
C	10.725	6.998	6.038
C	10.866	7.371	4.697
H	11.066	6.643	3.937
C	9.747	4.76	5.38
C	8.31	4.297	3.707
C	8.82	6.978	7.711
H	9.055	7.851	7.138
C	10.449	5.563	6.494
C	7.454	5.393	1.507
H	6.549	5.9	1.242
H	8.186	6.11	1.815
C	9.674	3.417	5.281
H	10.2	2.706	5.886
C	7.855	5.617	9.11
C	7.863	6.886	8.678
H	7.225	7.676	9.017
C	10.615	9.282	6.697
H	10.62	10.017	7.474
C	7.18	4.433	2.643

C	8.756	3.124	4.256
H	8.452	2.137	3.958
C	10.449	9.656	5.413
C	11.712	4.808	6.95
H	12.185	5.349	7.743
H	12.39	4.717	6.127
H	11.44	3.833	7.297
C	6.731	5.145	10.033
C	6.273	6.322	10.913
H	5.971	7.137	10.29
H	5.449	6.013	11.521
C	6.968	3.106	1.955
H	6.266	3.226	1.156
C	10.697	8.732	4.374
H	10.739	9.059	3.357
C	10.753	7.935	7.012
H	10.857	7.636	8.035
C	7.154	3.983	10.952
H	7.482	3.158	10.355
H	7.953	4.304	11.587
N	4.901	5.591	8.319
H	4.92	6.587	8.408
O	3.529	11.116	6.385
N	5.047	4.422	4.189
H	4.776	3.462	4.15
C	4.194	4.898	7.414
C	3.117	6.963	6.341
C	3.098	7.685	5.147
H	3.07	7.171	4.21
C	4.473	5.486	5.037

C	6.042	5.018	3.501
C	4.284	3.582	7.725
H	3.753	2.78	7.256
C	3.456	5.469	6.183
C	5.048	6.641	4.67
H	4.812	7.609	5.063
C	5.615	4.722	9.062
C	5.217	3.467	8.757
H	5.556	2.56	9.213
C	2.966	9.02	7.555
H	2.844	9.541	8.482
C	5.997	6.366	3.707
H	6.609	7.098	3.221
C	3.198	9.72	6.399
C	2.213	4.603	5.901
H	1.545	4.653	6.735
H	1.715	4.965	5.024
H	2.515	3.588	5.744
H	7.082	6.633	11.541
H	6.589	2.395	2.659
C	3.144	9.067	5.175
H	3.156	9.626	4.263
C	2.91	7.599	7.524
H	2.733	7.042	8.421
H	6.321	3.681	11.552
N	8.438	13.179	6.199
C	7.721	14.316	6.369
C	9.769	15.598	6.178
H	10.276	16.539	6.134
C	8.379	15.554	6.302

C	7.658	16.752	6.368
H	8.17	17.686	6.263
C	9.796	13.222	6.117
C	10.469	14.448	6.12
H	11.537	14.472	6.072
N	5.63	13.149	6.785
C	6.322	14.302	6.626
C	4.274	15.541	7.02
H	3.746	16.47	7.071
C	5.641	15.525	6.735
C	6.321	16.738	6.568
H	5.78	17.66	6.606
C	4.317	13.165	7.142
C	3.625	14.378	7.238
H	2.584	14.384	7.485
C	10.628	11.919	6.033
H	11.604	12.126	5.645
H	10.714	11.509	7.018
C	3.575	11.841	7.456
H	4.107	11.322	8.225
H	2.577	12.043	7.787
H	7.819	4.85	0.661
H	7.899	2.755	1.561

2·LiCl

Cartesian coordinates:

Symbol	X	Y	Z
N	2.93158	-1.9923	-0.96238
H	2.15674	-1.3585	-0.81394
O	-3.09737	-3.17171	-2.26207

N	1.19872	-1.84963	1.89373
H	0.62408	-1.42719	1.17147
C	2.99749	-3.27032	-0.44569
C	0.53885	-3.77034	-0.27984
C	-0.65496	-3.80385	0.44599
H	-0.62085	-3.90669	1.52616
C	1.83847	-3.07009	1.76142
C	1.31065	-1.38214	3.19046
C	4.15332	-3.84063	-0.93897
H	4.50931	-4.83876	-0.72711
C	1.90175	-3.83473	0.44371
C	2.38759	-3.36806	2.99157
H	2.97015	-4.24661	3.22987
C	4.01036	-1.74114	-1.78398
C	4.78958	-2.88128	-1.77592
H	5.71848	-3.01779	-2.31078
C	-0.77043	-3.52376	-2.31494
H	-0.83294	-3.41571	-3.39317
C	0.68155	-0.07909	3.66615
C	2.05913	-2.31261	3.88407
H	2.34287	-2.24821	4.92477
C	-1.93957	-3.46431	-1.55914
C	2.21577	-5.32	0.73966
H	2.24052	-5.89137	-0.19246
H	1.44721	-5.7449	1.39075
H	3.18588	-5.42479	1.23379
C	4.1745	-0.4247	-2.52637
C	2.92034	-0.15818	-3.39966
H	1.99362	-0.10852	-2.81731
H	3.02904	0.78684	-3.94411

C	0.52853	-0.13943	5.19865
H	0.0523	0.77477	5.56673
C	-1.89344	-3.64958	-0.17731
H	-2.7935	-3.63204	0.42779
C	0.4532	-3.67752	-1.67605
H	1.36037	-3.69454	-2.26975
C	5.38944	-0.53261	-3.4676
H	6.30945	-0.73887	-2.91351
H	5.23912	-1.34339	-4.18732
N	3.37044	1.38407	-0.9196
H	2.3885	1.23791	-1.11504
O	-1.78588	4.20451	-2.43793
N	1.76407	1.52299	2.00897
H	1.3463	1.08701	1.19672
C	3.85667	2.42365	-0.14647
C	1.69051	3.70587	-0.15262
C	0.45579	3.91052	0.47359
H	0.3905	3.85247	1.55503
C	2.55283	2.65691	1.9715
C	1.54428	1.12346	3.3115
C	5.22988	2.40319	-0.27325
H	5.92172	3.07328	0.217
C	2.94658	3.33504	0.66685
C	2.84766	2.97908	3.28066
H	3.45383	3.81176	3.6077
C	4.40445	0.73067	-1.56348
C	5.57254	1.34379	-1.15761
H	6.57164	1.06706	-1.4626
C	0.60253	4.05807	-2.3012
H	0.64327	4.11111	-3.38486

C	2.21808	2.0181	4.11964
H	2.26103	1.9896	5.19889
C	-0.63019	4.14679	-1.66051
C	3.70714	4.64193	0.99145
H	4.00656	5.14104	0.06614
H	3.0653	5.31849	1.56254
H	4.60485	4.43736	1.58184
H	2.80027	-0.9671	-4.12837
H	-0.09049	-0.99626	5.48089
C	-0.70068	4.13453	-0.26956
H	-1.65688	4.25317	0.22866
C	1.75404	3.84976	-1.54614
H	2.70899	3.74758	-2.05179
H	5.52425	0.40389	-4.01677
N	-4.46569	-1.2032	-0.82088
C	-5.11732	-0.23473	-0.12133
C	-6.57914	-1.87133	0.88997
H	-7.38469	-2.13468	1.57016
C	-6.1886	-0.52027	0.76393
C	-6.84833	0.5408	1.46273
H	-7.6476	0.2893	2.15391
C	-4.89923	-2.4535	-0.73143
C	-5.95427	-2.83151	0.13041
H	-6.25698	-3.87284	0.18458
N	-3.74033	1.40567	-1.23101
C	-4.74198	1.14545	-0.34894
C	-5.12334	3.50589	-0.00151
H	-5.6558	4.32054	0.48233
C	-5.46024	2.17084	0.31598
C	-6.4984	1.83772	1.24402

H	-7.01243	2.64508	1.75775
C	-3.46027	2.67058	-1.52547
C	-4.14363	3.75682	-0.93124
H	-3.86402	4.76411	-1.21547
C	-4.32342	-3.50706	-1.66277
H	-4.25663	-4.47175	-1.14245
H	-5.0318	-3.63157	-2.49173
C	-2.40929	2.93151	-2.58644
H	-1.66738	2.12467	-2.57512
H	-2.90057	2.94105	-3.56845
H	1.49693	-0.23954	5.69644
C	-0.73581	0.09371	3.06012
H	-1.36838	-0.76033	3.33067
H	-1.19553	1.0064	3.45562
H	-0.72622	0.17625	1.96866
Cl	-0.35668	-0.00041	-0.83783
Li	-2.54946	-0.36299	-1.35167

2·$MgCl_2$

Cartesian coordinates:

Symbol	X	Y	Z
N	2.56901	1.49006	2.13097
H	2.51235	0.86192	1.34293
O	-2.13003	3.96025	-1.86486
N	3.34781	1.75857	-1.12873
H	2.37408	1.75098	-1.39845
C	2.88989	2.82786	2.03374
C	1.59876	3.72943	0.05363
C	1.4805	4.18935	-1.26914
H	2.37618	4.42999	-1.83558

C	3.87297	2.68713	-0.2435
C	4.3614	1.13841	-1.84344
C	2.99937	3.30307	3.32491
H	3.23372	4.31914	3.60937
C	2.99676	3.52264	0.68698
C	5.24524	2.60097	-0.3496
H	5.96063	3.16053	0.23691
C	2.48232	1.09716	3.44805
C	2.74514	2.21798	4.21213
H	2.75362	2.26272	5.29192
C	-0.82806	3.56604	0.14584
H	-1.72502	3.30817	0.69892
C	4.0877	0.13777	-2.95745
C	5.54975	1.63421	-1.3475
H	6.53909	1.33721	-1.66665
C	-0.91545	3.94527	-1.19049
C	3.65178	4.9098	0.89153
H	3.0255	5.53087	1.53856
H	3.76955	5.42123	-0.06709
H	4.63683	4.81137	1.35625
C	2.05098	-0.30642	3.84381
C	0.51229	-0.42492	3.67792
H	0.17837	-0.21096	2.65725
H	0.16883	-1.42989	3.95226
C	5.16479	0.29781	-4.04882
H	4.96806	-0.3841	-4.88245
C	0.23872	4.29257	-1.89295
H	0.15386	4.60558	-2.92885
C	0.42055	3.45629	0.75596
H	0.47405	3.12561	1.78646

C	2.38786	-0.53074	5.33214
H	3.45724	-0.40711	5.52462
H	1.84814	0.18878	5.95596
N	2.2527	-1.8614	1.81921
H	1.32625	-1.65726	1.46444
O	-2.83496	-2.9612	-0.86528
N	3.4118	-1.65135	-1.30608
H	3.00205	-0.99572	-0.65688
C	3.03313	-2.91423	1.35863
C	1.22663	-3.67591	-0.22757
C	0.71682	-3.39612	-1.50059
H	1.39917	-3.23348	-2.32715
C	3.49907	-3.00909	-1.08389
C	4.10747	-1.29076	-2.43856
C	4.0806	-3.04732	2.24499
H	4.89596	-3.75172	2.15803
C	2.74716	-3.66663	0.05992
C	4.26199	-3.53016	-2.10934
H	4.51498	-4.57209	-2.24662
C	2.75458	-1.37622	3.01927
C	3.90715	-2.08853	3.27965
H	4.55794	-1.94579	4.13068
C	-1.06148	-3.78509	0.61154
H	-1.73532	-3.88544	1.45667
C	4.64515	-2.45152	-2.95909
H	5.24377	-2.52768	-3.85569
C	-1.5227	-3.40342	-0.64451
C	3.22621	-5.1307	0.20382
H	2.71783	-5.6165	1.04087
H	3.00266	-5.69119	-0.70859

H	4.30484	-5.17433	0.3783
H	0.01972	0.30009	4.33387
H	5.16229	1.32196	-4.43334
C	-0.65306	-3.26938	-1.71889
H	-1.04608	-2.98145	-2.68889
C	0.31072	-3.93516	0.80293
H	0.67583	-4.1882	1.79405
H	2.09403	-1.53926	5.63779
N	-4.17183	1.19682	-0.72988
C	-5.2568	0.96103	0.05011
C	-5.76403	3.31281	0.16678
H	-6.37958	4.13757	0.51598
C	-6.10457	1.98798	0.52509
C	-7.24213	1.65166	1.3305
H	-7.88115	2.45647	1.68263
C	-3.88429	2.44908	-1.07296
C	-4.66143	3.54248	-0.62312
H	-4.36285	4.54223	-0.91514
N	-4.70134	-1.3661	-0.03808
C	-5.54804	-0.40624	0.40599
C	-6.91038	-2.07351	1.48716
H	-7.77178	-2.35363	2.08732
C	-6.683	-0.70903	1.19329
C	-7.52587	0.35658	1.64851
H	-8.39318	0.11284	2.25504
C	-4.92461	-2.6371	0.25627
C	-6.04155	-3.03639	1.02021
H	-6.2009	-4.0874	1.24018
C	-2.72373	2.67012	-2.02019
H	-1.98006	1.88061	-1.88652

H	-3.09903	2.60388	-3.049
C	-3.93436	-3.64638	-0.28549
H	-3.60534	-4.31863	0.51257
H	-4.41601	-4.25917	-1.06002
H	6.16301	0.07724	-3.6604
C	2.70217	0.41088	-3.60104
H	2.63505	1.44099	-3.97032
H	2.54839	-0.2692	-4.44412
H	1.87765	0.23588	-2.90015
Cl	-1.09762	-0.10631	0.06023
Cl	-3.14544	-0.73264	-3.4754
Mg	-2.97509	-0.69773	-1.18749

2·LiBr

Cartesian coordinates:

Symbol	X	Y	Z
N	2.83505	2.07058	0.95043
H	2.01183	1.4794	0.92618
O	-3.15588	3.07119	2.29177
N	1.16001	1.86882	-1.93406
H	0.71001	1.37445	-1.1704
C	2.90652	3.31829	0.36211
C	0.43143	3.77279	0.25311
C	-0.77232	3.75234	-0.45642
H	-0.75512	3.84174	-1.53801
C	1.6968	3.13745	-1.82381
C	1.20088	1.43446	-3.24424
C	4.11849	3.86501	0.73157
H	4.49531	4.8335	0.43504
C	1.77964	3.87691	-0.49348

C	2.10149	3.51095	-3.0894
H	2.57339	4.44622	-3.35514
C	3.96481	1.82138	1.70274
C	4.78242	2.92649	1.56908
H	5.75551	3.05341	2.02129
C	-0.84017	3.50206	2.30975
H	-0.88367	3.40154	3.3896
C	0.65867	0.08134	-3.68692
C	1.79308	2.44459	-3.97768
H	1.98769	2.42593	-5.04042
C	-2.01688	3.39369	1.57139
C	2.05827	5.37293	-0.76851
H	2.10751	5.92517	0.17394
H	1.26082	5.79809	-1.38395
H	3.00796	5.50324	-1.29515
C	4.15209	0.53797	2.49799
C	2.90204	0.26719	3.37627
H	1.98143	0.14426	2.7965
H	3.04847	-0.64509	3.96562
C	0.48528	0.10132	-5.21822
H	0.08128	-0.85501	-5.56429
C	-1.99581	3.56408	0.18644
H	-2.9036	3.51227	-0.4051
C	0.36799	3.69479	1.65152
H	1.2813	3.75485	2.23289
C	5.35582	0.71488	3.44339
H	6.27687	0.91505	2.88889
H	5.18058	1.55397	4.12364
N	3.42658	-1.28353	0.86835
H	2.43784	-1.07987	0.94792

O	-1.6827	-4.26446	2.3229
N	1.77286	-1.5072	-2.0269
H	1.23299	-1.18987	-1.22984
C	3.94277	-2.34911	0.15455
C	1.8155	-3.68288	0.09911
C	0.60593	-3.95983	-0.54836
H	0.56118	-3.92752	-1.63216
C	2.67354	-2.55592	-1.99062
C	1.60672	-1.05301	-3.3211
C	5.29947	-2.3747	0.40296
H	6.00664	-3.08065	-0.00866
C	3.06823	-3.25859	-0.69787
C	3.09763	-2.76147	-3.28761
H	3.81455	-3.50267	-3.61066
C	4.42242	-0.64859	1.58429
C	5.60001	-1.30949	1.29575
H	6.57742	-1.06049	1.68331
C	0.70196	-4.04301	2.23137
H	0.722	-4.07001	3.31659
C	2.43191	-1.82041	-4.1195
H	2.55138	-1.72017	-5.18869
C	-0.51227	-4.19451	1.56827
C	3.8662	-4.53626	-1.04731
H	4.15627	-5.05914	-0.1321
H	3.25346	-5.20706	-1.65572
H	4.77276	-4.29174	-1.60821
H	2.74503	1.10419	4.06595
H	-0.20326	0.90084	-5.5083
C	-0.55598	-4.2163	0.17646
H	-1.49752	-4.38293	-0.33626

C	1.85763	-3.79739	1.49571
H	2.79607	-3.63882	2.01675
H	5.50587	-0.19284	4.03552
N	-4.4892	1.11218	0.81704
C	-5.12618	0.1439	0.10288
C	-6.66818	1.75813	-0.82013
H	-7.49926	2.01426	-1.47174
C	-6.22696	0.41865	-0.74836
C	-6.86465	-0.6435	-1.46585
H	-7.68666	-0.39959	-2.1326
C	-4.96835	2.34836	0.77775
C	-6.05905	2.71487	-0.04371
H	-6.39968	3.74577	-0.05559
N	-3.67856	-1.47735	1.1435
C	-4.70166	-1.22815	0.28334
C	-5.0195	-3.58956	-0.11254
H	-5.53428	-4.40874	-0.60773
C	-5.40074	-2.25837	-0.3939
C	-6.46677	-1.93366	-1.29292
H	-6.9647	-2.74274	-1.81961
C	-3.36382	-2.74026	1.41238
C	-4.02332	-3.83214	0.80205
H	-3.71496	-4.8366	1.06517
C	-4.39726	3.39572	1.71822
H	-4.35368	4.36954	1.21236
H	-5.09434	3.49506	2.55998
C	-2.30312	-2.9894	2.46562
H	-1.55975	-2.18469	2.43193
H	-2.78429	-2.98181	3.45274
H	1.43685	0.27148	-5.7292

C	-0.73879	-0.1708	-3.06248
H	-1.42754	0.63254	-3.35011
H	-1.14184	-1.12283	-3.42669
H	-0.71468	-0.21684	-1.96961
Li	-2.54091	0.33042	1.25633
Br	-0.18315	-0.06194	0.82796

2·MgBr$_2$

Cartesian coordinates:

Symbol	X	Y	Z
N	4.05143	1.34992	1.96117
H	4.94005	1.57316	1.5392
O	-2.68743	2.71559	-0.41449
N	4.0082	1.92842	-0.97465
H	3.54248	1.06929	-0.72076
C	2.88397	2.04523	1.70524
C	1.3786	3.39748	0.33412
C	0.9222	2.54659	-0.68457
H	1.61992	1.94756	-1.25551
C	3.81179	3.14214	-0.3491
C	4.80322	2.05953	-2.08874
C	1.87851	1.40987	2.41025
H	0.82918	1.66698	2.41032
C	2.84738	3.29166	0.8209
C	5.21116	1.27134	-4.4345
H	5.46832	0.41193	-5.06233
H	4.25515	1.67502	-4.77866
C	4.54573	4.06955	-1.06664
H	4.61573	5.12658	-0.84995
C	3.8118	0.28041	2.80085

C	2.46089	0.3114	3.09788
H	1.93149	-0.3954	3.72162
C	-0.94695	4.04278	0.72855
H	-1.65514	4.62897	1.3035
C	5.13517	0.84559	-2.95254
C	5.1654	3.39265	-2.15652
H	5.80545	3.84052	-2.90455
C	-1.36031	3.10209	-0.20592
C	3.27555	4.52648	1.65015
H	2.68204	4.60775	2.56369
H	3.16307	5.45087	1.07339
H	4.32499	4.43039	1.94214
C	4.84164	-0.79867	3.07137
C	4.55645	-1.44424	4.44862
H	3.5631	-1.90107	4.47373
H	5.28852	-2.23219	4.65202
C	6.51012	0.2727	-2.53223
H	6.76226	-0.62182	-3.11471
C	-0.42643	2.3964	-0.96169
H	-0.76948	1.67204	-1.69238
C	0.42361	4.17645	0.99172
H	0.72127	4.87291	1.76738
C	6.27096	-0.21165	3.09506
H	6.55856	0.20818	2.1244
H	6.3543	0.5723	3.85566
N	3.70143	-1.9289	1.09727
H	2.93297	-1.26997	1.09979
O	-2.6986	-3.7723	0.17695
N	4.17361	-1.30572	-1.97941
H	5.02892	-1.61179	-1.5382

C	3.82512	-3.01011	0.24637
C	1.42384	-3.47935	-0.56343
C	0.49346	-3.80622	-1.56639
H	0.82266	-3.92717	-2.59287
C	2.96259	-1.94416	-1.79645
C	4.03141	-0.1865	-2.77246
C	4.99929	-3.65094	0.60421
H	5.40623	-4.53513	0.1344
C	2.89797	-3.21708	-0.94582
C	2.01926	-1.21296	-2.49709
H	0.95674	-1.40589	-2.53548
C	4.75215	-1.86974	1.98465
C	5.57826	-2.93926	1.69302
H	6.49629	-3.19189	2.20643
C	-0.42885	-3.45478	1.0262
H	-0.76884	-3.32134	2.04791
C	2.69193	-0.12157	-3.11422
H	2.23105	0.65602	-3.70893
C	-1.33491	-3.70611	-0.00182
C	3.44089	-4.41783	-1.76698
H	3.41486	-5.33504	-1.16965
H	2.84243	-4.57357	-2.66618
H	4.47224	-4.23473	-2.08317
H	4.61209	-0.69524	5.24616
H	6.53428	0.01649	-1.46655
C	-0.86401	-3.91694	-1.29873
H	-1.57828	-4.11074	-2.09123
C	0.93474	-3.34458	0.73812
H	1.6144	-3.14126	1.55719
H	6.99948	-0.99369	3.33008

N	-4.9664	1.48421	-0.03475
C	-6.07706	0.72737	-0.16761
C	-7.38342	2.69036	-0.65475
H	-8.32628	3.16559	-0.91146
C	-7.33405	1.28769	-0.49516
C	-8.46735	0.4216	-0.6436
H	-9.42563	0.8583	-0.90944
C	-5.03044	2.80076	-0.1781
C	-6.24143	3.44914	-0.48815
H	-6.26407	4.5272	-0.6082
N	-4.73942	-1.16573	0.44112
C	-5.9517	-0.69254	0.0612
C	-6.91858	-2.89461	0.16274
H	-7.76119	-3.57139	0.04972
C	-7.09214	-1.51421	-0.08551
C	-8.34682	-0.92293	-0.45379
H	-9.20969	-1.57287	-0.56825
C	-4.60705	-2.46034	0.71208
C	-5.68747	-3.36089	0.565
H	-5.52638	-4.41295	0.77427
C	-3.73621	3.54336	0.06317
H	-3.72744	4.51291	-0.44949
H	-3.62717	3.71649	1.14344
C	-3.2613	-2.95103	1.19674
H	-2.62376	-2.09464	1.4311
H	-3.39722	-3.53616	2.11853
H	5.9755	2.04299	-4.57781
H	7.28982	1.02148	-2.70023
Mg	-3.09004	0.41527	0.11467
Br	-2.0707	0.5587	2.30864

Br	-2.3453	-0.57239	-1.97651

2·$CaBr_2$

Cartesian coordinates:

Symbol	X	Y	Z
N	4.14811	0.73962	2.26012
H	5.01818	1.08937	1.88821
O	-2.61545	2.49854	0.36873
N	4.03369	2.04638	-0.43368
H	3.47505	1.20648	-0.4858
C	2.96185	1.44737	2.21699
C	1.43083	3.09803	1.23648
C	1.01995	2.86978	-0.08343
H	1.75357	2.71328	-0.86457
C	3.88733	3.02852	0.52487
C	4.93691	2.41937	-1.40138
C	1.98827	0.62181	2.75307
H	0.93344	0.8337	2.84856
C	2.90243	2.88025	1.67625
C	5.40343	2.30706	-3.86505
H	5.66763	1.65217	-4.70191
H	4.46619	2.81488	-4.10819
C	4.75396	4.04683	0.16954
H	4.89903	4.97221	0.70932
C	3.95536	-0.51652	2.79766
C	2.61281	-0.5999	3.1219
H	2.11251	-1.45792	3.54925
C	-0.91892	3.23555	1.87542
H	-1.68223	3.33466	2.63985
C	5.27301	1.48654	-2.56372

C	5.40973	3.66705	-1.03665
H	6.15052	4.24718	-1.57012
C	-1.28614	2.89177	0.57895
C	3.31306	3.87917	2.78831
H	2.71453	3.73456	3.68967
H	3.1914	4.91343	2.44965
H	4.36133	3.72427	3.05901
C	5.00344	-1.61062	2.72732
C	4.82756	-2.56917	3.92859
H	3.83378	-3.02556	3.92926
H	5.56406	-3.37751	3.87728
C	6.62388	0.78492	-2.28437
H	6.87667	0.08048	-3.08591
C	-0.32861	2.75588	-0.42257
H	-0.62312	2.45632	-1.42441
C	0.43376	3.33917	2.19388
H	0.70116	3.53751	3.22545
C	6.43221	-1.02304	2.77532
H	6.64136	-0.38224	1.91098
H	6.5816	-0.43927	3.69038
N	3.76167	-2.15919	0.57993
H	3.0376	-1.48017	0.77623
O	-2.71781	-2.54607	-0.44318
N	4.21289	-0.79819	-2.18171
H	5.03559	-1.23398	-1.79039
C	3.79224	-3.00552	-0.51078
C	1.34212	-3.05346	-1.30078
C	0.36527	-2.99944	-2.30833
H	0.65896	-2.94328	-3.35034
C	2.97697	-1.41428	-2.21031

C	4.14762	0.47523	-2.70722
C	4.92156	-3.79219	-0.3564
H	5.25719	-4.55921	-1.04007
C	2.82791	-2.84957	-1.67946
C	2.09748	-0.50517	-2.77734
H	1.03417	-0.62738	-2.92268
C	4.82162	-2.39166	1.42462
C	5.56463	-3.41024	0.85561
H	6.4689	-3.84199	1.26323
C	-0.46062	-3.09395	0.35093
H	-0.76426	-3.0634	1.39113
C	2.83405	0.66777	-3.09502
H	2.42624	1.57509	-3.5202
C	-1.38871	-2.91717	-0.67075
C	3.23709	-3.8625	-2.7807
H	3.14896	-4.88974	-2.41176
H	2.60322	-3.75467	-3.66272
H	4.27107	-3.68971	-3.09326
H	4.96024	-2.03122	4.87351
H	6.61066	0.24289	-1.33147
C	-0.9891	-2.92649	-2.00532
H	-1.7298	-2.7864	-2.78411
C	0.89616	-3.16943	0.02111
H	1.61002	-3.27248	0.82919
H	7.17329	-1.82803	2.75897
N	-4.89896	1.36571	-0.18555
C	-6.09779	0.75224	-0.08438
C	-7.24065	2.84084	-0.44887
H	-8.15412	3.42123	-0.54724
C	-7.32242	1.45208	-0.2002

C	-8.55915	0.73895	-0.05306
H	-9.49036	1.29134	-0.14215
C	-4.8418	2.67225	-0.40206
C	-6.00812	3.45287	-0.54889
H	-5.92834	4.52075	-0.7255
N	-4.92712	-1.31816	0.22875
C	-6.11221	-0.67511	0.15999
C	-7.29603	-2.73394	0.56193
H	-8.22051	-3.29137	0.68671
C	-7.35032	-1.34425	0.31213
C	-8.57246	-0.60048	0.19891
H	-9.51437	-1.12936	0.31428
C	-4.89612	-2.62557	0.4486
C	-6.07649	-3.37636	0.63038
H	-6.01836	-4.44541	0.80791
C	-3.45281	3.26838	-0.50028
H	-3.09083	3.18984	-1.53512
H	-3.45203	4.32014	-0.19118
C	-3.51737	-3.24892	0.51299
H	-3.10668	-3.11137	1.52271
H	-3.54922	-4.31678	0.26755
H	6.18305	3.06981	-3.76453
H	7.4225	1.52966	-2.21791
Br	-1.99521	-0.6045	2.58675
Br	-2.18381	0.52826	-2.73951
Ca	-2.72856	-0.00828	-0.0443

References

1. R. E. Gaussian 09, Frisch, M. J.; Trucks, G. W.; Schlegel, H. B.; Scuseria, G. E.; Robb, M. A.; Cheeseman, J. R.; Scalmani, G.; Barone, V.; Mennucci, B.; Petersson, G. A.; Nakatsuji, H.; Caricato, M.; Li, X.; Hratchian, H. P.; Izmaylov, A. F.; Bloino, J.; Zheng, G.; Sonnenberg, J. L.; Hada, M.; Ehara, M.; Toyota, K.; Fukuda, R.; Hasegawa, J.; Ishida, M.; Nakajima, T.; Honda, Y.; Kitao, O.; Nakai, H.; Vreven, T.; Montgomery, Jr., J. A.; Peralta, J. E.; Ogliaro, F.; Bearpark, M.; Heyd, J. J.; Brothers, E.; Kudin, K. N.; Staroverov, V. N.; Kobayashi, R.; Normand, J.; Raghavachari, K.; Rendell, A.; Burant, J. C.; Iyengar, S. S.; Tomasi, J.; Cossi, M.; Rega, N.; Millam, J. M.; Klene, M.; Knox, J. E.; Cross, J. B.; Bakken, V.; Adamo, C.; Jaramillo, J.; Gomperts, R.; Stratmann, R. E.; Yazyev, O.; Austin, A. J.; Cammi, R.; Pomelli, C.; Ochterski, J. W.; Martin, R. L.; Morokuma, K.; Zakrzewski, V. G.; Voth, G. A.; Salvador, P.; Dannenberg, J. J.; Dapprich, S.; Daniels, A. D.; Farkas, Ö.; Foresman, J. B.; Ortiz, J. V.; Cioslowski, J.; Fox, D. J., Gaussian, Inc., Wallingford CT, 2009.

2. X. Xu and W. A. Goddard, The X3LYP extended density functional for accurate descriptions of nonbond interactions, spin states, and thermochemical properties, *P. Natl. Acad. Sci.* USA, 2004, ***101***, 2673-2677.

3. S. F. Boys and F. Bernardi, The calculation of small molecular interactions by the differences of separate total energies. Some procedures with reduced errors (Reprinted from Molecular Physics, vol 19, pp 553-566, 1970), *Mol. Phys.*, 2002, ***100***, 65-73.

4. F. B. van Duijneveldt, J. G. C. M. van Duijneveldt-van de Rijdt and J. H. van Lenthe, State of the Art in Counterpoise Theory, *Chem. Rev.*, 1994, ***94***, 1873-1885.

5. K. Wang, C.-C. Yee and H. Y. Au-Yeung, *Chem. Sci.* 2016, *7*, 2787-2792.

6. J. Yoo, I.-W. Park, T.-Y. Kim and C.-H. Lee, *Bull. Korean Chem. Soc.* 2010, ***31***, 630-634.
